# Gut microbiome-derived propionate reprograms alveolar macrophages metabolically and regulates lung injury responses in mice

**DOI:** 10.1080/19490976.2025.2606486

**Published:** 2025-12-30

**Authors:** Daisuke Maruyama, Xiaoli Tian, Thien N. M. Doan, Wen-I Liao, Tomohiro Chaki, Hiroki Taenaka, Mazharul Maishan, Michael A. Matthay, Arun Prakash

**Affiliations:** a Department of Anesthesia and Perioperative Care, University of California San Francisco and San Francisco General Hospital, San Francisco, CA, USA; b Department of Emergency Medicine, Tri-Service General Hospital, National Defense Medical Center, Taipei, Taiwan; c Department of Anesthesiology, Sapporo Medical University School of Medicine, Sapporo, Japan; d Departments of Medicine and Anesthesia, University of California San Francisco, San Francisco, CA, USA

**Keywords:** Gut-lung axis, SCFA, propionate, FFAR, gut microbiome, lung immune tone, lung metabolic tone, immunometabolic tone, lung injury, metabolites

## Abstract

Responses to lung injury can vary between individuals with the diet and gut microbiome representing two underappreciated sources for this variability. The gut microbiome can influence lung injury outcomes through the gut‒lung axis, but exactly how diet and its effects on the microbiota are involved remains unclear. We hypothesized that dietary fiber interventions would favor the presence of short-chain fatty acid (SCFA)-producing fermentative bacteria presence in the gut microbiome, thereby influencing the resting lung immunometabolic tone as well as influencing downstream responses to lung injury and infection. To test this hypothesis, we fed mice fiber-rich (FR) and fiber-free (FF) diets, and observed changes in the steady-state transcriptional programming of alveolar macrophages (AM). Next, we examined the effects of the FR and FF diets on murine responses to sterile and infectious lung injury *in vivo* while simultaneously profiling the gut microbiota and SCFA levels transmitted along the gut‒lung axis. Finally, we validated our *in vivo* observations with mechanistic studies of the metabolic, signaling, and chromatin-modifying effects of specific SCFAs on lung AM *ex vivo* and *in vitro*. Overall, our fiber-rich diet reprogrammed AMs and attenuated lung inflammation after sterile injury while exacerbating lung infection. This effect of FR diets could be transferred to germ-free (GF) mice by fecal microbiome transplantation (FMT) and depended on the ability of the microbiota to produce propionate. Mechanistically, SCFAs altered the metabolic programming of AMs and lung tissue *ex vivo* without a clear role for free fatty acid receptors (FFAR) or chromatin remodeling. These findings demonstrate that the gut‒lung axis can regulate resting lung metabolic tone through dietary fiber intake and the enrichment of SCFA-producing gut bacteria, as well as influence sterile and non-sterile lung injury responses. These results provide evidence to support the development of therapeutic dietary interventions to preserve or enhance specific aspects of host pulmonary immunity.

## Graphical abstract

**Figure:**
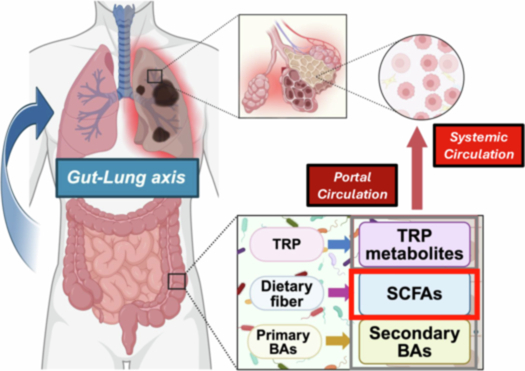

## Introduction

The influence of the gut microbiome on human health and disease has been progressively elucidated over the past decade,^1-4^ but how the gut microbiota control lung immune injury responses via the gut‒lung axis is not well understood.^2^^,^^5^^,^^6^ High-fiber diets have been shown to promote the expansion of fermenting gut bacteria, resulting in the production of important metabolites, including SCFAs – those with acyl chains up to 6 carbons in length. SCFA metabolites are now well recognized as immune and metabolic regulators of gut health and permeability and have also been implicated in modulating health and disease states throughout the body.^1^^,^^6-10^ However, the key mechanisms by which these changes in the diet and gut microbiome tune lung metabolic and immune states remain undefined, as do the specific effects of key bacterial species and metabolites. The emerging intersection and interactions between metabolism and immunity have been termed immunometabolism and can be studied both at the cellular and organismal levels, with key roles for the diet and the gut microbiome (reviewed in^11-17^). Therefore, the identification of dietary factors, specific gut bacteria and bioactive metabolites that influence lung immune and injury responses could lead to a better understanding of inter-individual immune variability and could be exploited for personalized therapeutic approaches and applications.

The immunometabolic tone of the lung at the tissue level refers to the level of immune priming and the baseline metabolic set-point present in the lung, which in turn may dictate the degree of inflammatory response following injury. Two key inflammasome-dependent cytokines, IL-1β and IL-18, may contribute to this tone and have been implicated in a variety of lung pathologies, including sterile injury,^18-21^ infection,^22^^,^^23^ and asthma.^24-26^ We previously reported that SCFA metabolites derived from the gut microbiome can translocate from the gut to the lung in mice and can regulate the IL-1β-dependent inflammatory output of alveolar macrophages (AM) *in vitro*.^21^^,^^27^

SCFA (specifically acetate/C2, propionate/C3, and butryate/C4) are primarily produced by fiber fermentation by gut bacteria and are thought to exert their biological effects on the host through three primary mechanisms (reviewed in^1^^,^^7^^,^^28^^,^^29^). First, SCFAs can serve as local energy sources for colonic epithelia and potentially other cell types. Second, propionate and butyrate can act as histone deacetylase inhibitors (HDACi) targeting specific gene loci. Finally, all three SCFAs can signal via free fatty acid receptors (FFAR – primarily FFAR2 and FFAR3), which are G-protein coupled receptors that can regulate gene transcription in the affected cell and influence inflammatory output.

In this study, we used pectin-based FR and FF diets to investigate the effects of fiber on lung immunometabolic tone at steady-state, specifically in alveolar macrophages (AM). We observed that dietary fiber could regulate lung inflammatory responses after sterile ischemia reperfusion (IR) injury, and correlated changes in lung inflammatory markers with specific gut bacterial taxa and SCFAs. We then investigated whether the diet-induced lung IR injury responses could be transferred to GF mice via fecal microbiome transplantation (FMT) and how dietary fiber might affect the course of bacterial pneumonia in mice. Finally, using *in vitro* and *ex vivo* cellular and tissue approaches, we mechanistically studied how C3 was able to alter the inflammatory responses and metabolic activity of AMs and whether these effects depended on FFAR signaling and/or changes in chromatin accessibility.

## Materials and methods

### Animal care

All studies were approved by the institutional animal care and use committee at the University of California, San Francisco (IACUC protocol# AN197325-01). All the mice were purchased from the Jackson Laboratory (Bar Harbor, ME) or bred in the animal facilities at the University of California, San Francisco. Wild-type (WT) C57BL/6 and GF (aka gnotobiotic) mice were used in this study (10–15-week-old GF or WT C57BL/6J adult male mice). Commercially purchased mice were allowed to acclimate to their new housing for at least 1 week before any experiments were performed. FFAR2 and FFAR3KO mice were generated as reported previously^30^^,^^31^ and generously provided by B. Layden (University of Illinois, Chicago).

### Fiber diet interventions

Purchased mice received standard animal facility chow (normal diet, i.e. ND) for at least 1 week or more after arriving at UCSF, after which they received either 2 weeks of 0% fiber (FF) or 2 weeks of 35% pectin fiber (FR 2wk or FR) or 1 week of FF followed by 1 week of FR (FR 1wk or FF-> FR) or continued on ND. Diets were purchased from Newco Distributors, Inc. FF: modified AIN-93G/no cellulose (Cat. no. 5GCX); FR diet: modified TestDiet® 57W5 with pectin (Cat. no. 5Z6R). The normal diet (ND) consisted of standard UCSF mouse chow (2.4% dietary fiber). Mouse fecal quality and intestinal morphology were observed (Figure S1B) after IR surgery, as well as weight changes over the course of the dietary intervention with no significant weight loss observed (Figure S1C). While these three diets were not isocaloric, the mice had *ad libitum* access to food and the choice of FF and FR diet allowed us to evaluate the role of fiber without the potentially confounding effects of making the diets isocaloric by adding fat or other calorie sources (Figures S1B, C). Additionally, energy sources generated from fiber fermentation and other bacterial breakdown were not accounted for in the diet kcal energy calculations.

### Left lung IR surgery

A mouse model of unilateral left pulmonary artery occlusion was used, as previously described.^32^ Briefly, anesthetized mice (using IP tribromoethanol (Avertin®); Sigma–Aldrich) were orally intubated, given buprenorphine (IP; Harry Schein, Melville, NY), and placed on a rodent ventilator, using tidal volumes of 225 µL (7.5 cc/kg) and a respiratory rate of 180 breaths/min (assuming an average mouse weight of 30 g). A left thoracotomy was performed via the intercostal space between the 2nd and 3rd ribs, and the left pulmonary artery (PA) was identified and ligated with a slip knot suture using 7-0 or 8-0 prolene monofilament suture. The end of the suture was externalized to the anterior chest wall through a narrow bore (27 g) needle. Before closing the chest cavity, the left lung was reinflated with positive end pressure. Local anesthesia (3–4 drops of 0.25% bupivacaine) was applied topically prior to skin closure. The total duration of mechanical ventilation and surgery was approximately 20–25 min. After skin closure, the mice were extubated and allowed to recover from anesthesia. After 60 min of ischemia, the pulmonary artery ligature was released, and left lung reperfusion was initiated. At the experimental end point (1 h post reperfusion), mice were euthanized, and the blood, feces, and lungs were collected.

Blood was collected from anesthetized mice through the inferior vena cava (IVC) and portal vein puncture with a heparinized syringe, centrifuged (14,000 × g, 5 min), and the plasma was separated, snap frozen in liquid nitrogen and stored at −80°C. The lower portions of the left lungs were excised and divided into two parts: one part was placed in either TRIzol® (Life Technologies, Carlsbad, CA) for RNA preparation and the second part was frozen at −80°C for homogenization for ELISA. Cytokines and chemokine levels were quantified in plasma or homogenized lung tissue by ELISA or in lung tissue by qPCR.

All the mice received equivalent durations of mechanical ventilation (~40–45 min) and were allowed to breathe spontaneously during their recovery from anesthesia and the remainder of the ischemia and subsequent reperfusion periods. Pre- and postoperative care was performed according to the ARRIVE guidelines.

The success rate for lung IR surgery is approximately 80%–90%. Mice that did not survive the IR surgery or the reperfusion period because of technical complications in the surgical procedure (predominantly, left bronchus or left PA injury) were mostly excluded from the study, except for the analysis of effects of the dietary fiber on 16S rRNA sequence microbiome composition. In the case of experiments involving limited availability of mice, namely, experiments involving the GF mice given FMTs, both the mice that survived IR surgery and those that did not were included (and the injury termed “lung injury”) with the rationale that the entry into the left thorax, left lung collapse, and left lung manipulation–all generated sterile lung injury and based on our prior work with human lung tissue, the affected lung tissue and cells would still be alive and undergoing inflammatory responses 2 h following the start of the surgical procedure.^21^

### Bulk RNAseq analysis of bronchoalveolar lavage (BAL) cells

Two groups of WT C57BL/6 mice (*n* = 10 each) were fed FR and FF diets for 2 weeks. BAL was performed as previously described^21^ and RNA quantity and quality were measured. Briefly, the mice were euthanized under anesthesia (Avertin plus buprenorphine), and their tracheas were surgically exposed. Alveolar lavage was performed by injecting and withdrawing 10  mL of ice-cold phosphate-buffered saline (PBS; Thermo Fisher Scientific, Waltham, MA) containing 2 mM EDTA (Corning costar, Corning, NY), into the trachea at a volume of 1  mL. The cells were pelleted at 300 × g for 4  min at 4°C and then resuspended in TRIzol.

A total of 100 ng of RNA from each sample was submitted to UCSF ImmunoX Genomics CoLabs for bulk RNAseq. RIN scores were all between 9 and 10. Illumina compatible RNA-sequencing libraries were generated from purified RNA using the Tecan Universal mRNA Plus kit (9156-A01) by the UCSF Genomics CoLabs. Libraries were sequenced on an Illumina NovaSeq X using paired end 50bp reads at the UCSF Center for Advanced Technology (https://cat.ucsf.edu). Sequencing reads were aligned to the mouse reference genome (GRCm38) and reads per gene matrix were counted using the Ensemble annotation build version 96 with STAR v2.7.5c.^33^ Read counts per gene were used as input to DESeq2^34^ to test for differential gene expression between conditions using a Wald test, while possible covariates were corrected. Genes passing a multiple testing correction with a *p*-value of 0.1 (FDR method) were considered significant. Differential gene expression was performed and heat maps were generated as shown. Gene enrichment analysis was also performed using MSigDB, KEGG, and GO databases.

### Reagents and cell lines

Propionate (catalog number 18108), acetate (catalog number 241245), and lipopolysaccharide (LPS, catalog number L4391, from *Escherichia coli* O111:B4) were purchased from Sigma−Aldrich (St. Louis, MO). Butyrate (catalog number AAA1107906) was purchased from Alfa Aesar (Haverhill, MA). Seahorse XF calibrant solution (part number 100840-000), Seahorse XF DMEM medium, pH7.4 (catalog number 103575-100), Seahorse XF RPMI medium, pH 7.4 (catalog number 103576-100), Seahorse XF1.0 M glucose solution (catalog number 103577-100), Seahorse XF 100 mM pyruvate solution (catalog number 103578-100), Seahorse XF 200 mM glutamine solution (catalog number 103579-100), and Seahorse XF Cell Mito Stress Test Kit (catalog number 103015-100, contains 1 each of oligomycin, FCCP, and rotenone/antimycin A.) were all purchased from Agilent Technologies, Inc. (Santa Clara, CA).

The cell lines used in this study were MLE-12 (WT FVB/N murine lung epithelial AT2 cell, catalog number CRL-2110) and MH-S (wild-type BALB/c alveolar macrophage, catalog number CRL-2019). Both were purchased from the ATCC (Manassas, VA); all the cells were incubated at 37°C under humidified 5% CO_2_ and then treated with 10–200 ng/mL LPS and/or SCFAs (concentrations as indicated) for 24 h (or other time points as indicated).

### Collection of murine primary alveolar macrophages by BAL

Alveolar macrophages were isolated via BAL from WT, FFAR2 KO, and FFAR3 KO mice that had not undergone surgery. BAL was performed and cells from 2 to 4 mice were pooled and pelleted at 300 × g for 4 min at 4 C. The pellets were resuspended in Roswell Park Memorial Institute (RPMI) 1640 medium (Thermo Fisher Scientific, Waltham, MA) supplemented with 10% fetal bovine serum (FBS) and 1% penicillin–streptomycin (P/S; Thermo Fisher Scientific, Waltham, MA), plated in a 48-well plate at 200,000 cells per well, and were incubated overnight at 37 C under humidified 5% CO_2_.

### *In vitro* and *ex vivo* nutritional IR injury

Primary alveolar macrophages (200,000 cells/well in RPMI1640) or MH-S (200,000 cells/well in RPMI1640) were seeded on a 48-well plate supplemented with 10% FBS and 1% P/S. All the cells were incubated at 37 C in humidified 5% CO_2_, and all the treatments were given 12–24 h after cell seeding. All the cells were exposed to LPS (200  ng/mL) overnight, and SCFAs were administered 4 h before LPS stimulation.

*In vitro* and *ex vivo* nutritional IR conditions were performed as previously described.^32^ Briefly, nutritional “ischemia” was established by first washing the respective cells twice with PBS and then replacing the medium with PBS for 1 h. We did not create hypoxia for the period of nutritional IR injury because our *in vivo* ventilated IR model experiences minimal periods of hypoxia (as described later). After the *in vitro* “ischemia” period (1 h), medium supplemented with 10% FBS was added for the “reperfusion” period, and the supernatants were collected at the designated times for enzyme-linked immunosorbent assay (ELISA) analysis.

### Fecal microbiome transplant (FMT) in germ-free (GF) mice

Two groups of GF mice (*n* = 10 each) were gavaged with homogenized colonic contents collected from mice on FR or FF diets (2 weeks). The GF mice received the FMT by gavage 2‒3x over the course of 2 weeks and were maintained on their respective FR or FF diets. Both groups were subjected to lung injury (described above), after which the left and right lungs were harvested, homogenized and analyzed by ELISA for markers of lung injury as shown. Colonic contents were collected and sent for 16S rRNA sequencing along with gavaged FR and FF FMT inputs. GF mice (on a normal diet/ND) were also gavaged 3X for 3 weeks with *B. theta* WT or propionate mutant *B. theta* prior to lung IR surgery (*B. theta* WT and mutant strains were generously provided by Eric Martens and described elsewhere),^35^ after which the lungs were harvested, plasma was collected, and colonic stool, and other samples were collected and analyzed as described.

### Bacterial lung infections with *Streptococcus pneumoniae*

Three groups of WT C57BL/6 mice (*n* = 10 each) were fed normal diets (ND), FR or FF diets for 2 weeks. These mice had baseline measurements of body weight, rectal temperature, and oxygen saturation by pulse oximetry. Pulse oximetry was measured using the Mouse Ox+ cervical collar system (Starr Life Sciences, Oakmont, PA) by monitoring the mice for 5 min for each measurement and taking the mean SpO_2_ for 10 consecutive seconds when the mice were not active, as calculated by the system software. The mice were inoculated intranasally (IN) with *S. pneumoniae* (1 × 10^8^ CFU) as previously reported^36^^,^^37^ without the use of any antibiotics. Body weight and temperature were also measured at 12 h, 24 h, and 36 h post infection, and oxygen saturation was measured at 36 h prior to the collection of blood and BAL. The total protein content in the BAL fluid was measured by bicinchoninic acid (BCA) protein assay (Thermo Fisher Scientific), and cell and differential counts were performed. Postmortem bacterial titers of BAL were measured by serial dilution and counting colony-forming units on sheep blood agar plates.

### Precision cut lung slices (PCLS) preparation and culture

Preparation of PCLS was based on previous reports in the literature,^38^^,^^39^ with some modifications as described here. Twelve-week-old male C57BL/6 mice were used to prepare precision-cut lung slices (PCLS). Anesthesia and analgesia were induced via intraperitoneal (IP) injection of avertin (2,2,2-tribromoethanol, Sigma–Aldrich, U.S.A.) at a dose of 250 mg/kg body weight, supplemented with IP buprenorphine (0.1 mg/kg body weight), to minimize pain and distress. A midline incision was then made to expose the thoracic cavity, and blood was thoroughly flushed from the vasculature by trans-cardiac perfusion with ice-cold phosphate-buffered saline (PBS) to ensure the complete removal of blood from the pulmonary vasculature. The trachea was carefully exposed and securely tied off with a suture, and the mouse lung was inflated with 1.5 mL of pre-warmed 2% low melting point agarose solution (Sigma–Aldrich, U.S.A.) via intratracheal injection. After inflation, the inflated lungs were covered with chilled gauze and kept at 4°C for 10 min to allow the agarose to solidify, thereby preserving the lung morphology before the lungs were dissected out.

The isolated lung tissue was processed using a compresstome VF-310-0Z (Precisionary, Natick, MA, U.S.A.) vibrating microtome. The lung tissue was affixed to the piston with cyanoacrylate adhesive, embedded in 4% low melting point agarose, and sliced into sections of 300 μm thickness. All PCLS were collected in chilled Hanks’ balanced salt solution (HBSS) (Life Technologies) and transferred to a pre-warmed 24-well plate at a rate of 2 slices per well. Shortly thereafter, all PCLS were standardized to 4 mm diameter round discs using a Biopunch, ID 4.0 mm (TED PELLA, INC., Redding, CA). To remove the agarose, the sections were washed three times in pre-warmed DMEM/F12 medium (Thermo Fisher Scientific) supplemented with 10% FBS, 100 U/mL penicillin, 100  μg/mL streptomycin, and 1.5  μg/mL amphotericin B (Gibco® by Life Technologies). Each wash cycle involved incubation of the slices in 500  μL of medium per well at 37°C and 5% CO_2_ for 1 h. After the final wash, all the discs were maintained in culture at 37°C and 5% CO_2_, with the culture medium was replenished for the first 24 h. The medium was then changed to DMEM/F12 without FBS supplemented only with 100 U/mL penicillin, 100 μg/mL streptomycin, and 1.5 μg/mL amphotericin B for continued culture.

The AlamarBlue cell viability assay (Thermo-Fisher Scientific) was used to confirm PCLS viability per the manufacturer’s instructions. Viability assessments were repeated at intervals throughout the experiment.

### Quantification of SCFAs

SCFA in mouse lung samples were quantified at the Metabolomics Core Facility at the University of Michigan. Sample extraction was performed using an aqueous extraction solvent containing 3% 1 M HCl (v/v) and isotope-labeled internal standards (d7-buytric acid and d11-hexanoic acid). The samples were then homogenized and centrifuged. The supernatants were transferred to new Eppendorf tubes for extraction with diethyl ether. After phase separation, the upper layer was transferred to an autosampler vial for gas chromatography‒mass spectrometry (GC‒MS) analysis. GC (Agilent 6890, Wilmington, DE) separation was performed using a ZB-Wax plus column, 0.25 μm × 0.25 mm × 30 m (Phenomenex, Torrance, CA). A single quadrupole mass spectrometer (Agilent, 5973 inert MSD) was used to identify and quantify SCFA using Agilent MassHunter software, version B.06 [14]. The absolute quantities of SCFA were normalized to the sample mass.

### Seahorse^TM^ extracellular flux metabolism analysis

Seahorse^TM^ Extracellular Flux Metabolism Analysis was used to investigate the metabolic responses of mouse precision-cut lung slices (PCLS) upon exposure to lipopolysaccharide (LPS) and SCFAs. In brief, PCLSs were individually placed into the XFe24 cell culture plate, with one PCLS per well. The culture medium comprised DMEM/F12-conditioned medium supplemented with 1% penicillin/streptomycin (P/S) and 1.5 μg/mL amphotericin B. All the treatments were administered 1 d after the preparation of PCLS, including LPS at a concentration of 100 ng/mL and propionate/C3 at concentrations of 0.1 mM or 5  mM.

For Seahorse^TM^ Extracellular Flux Metabolism Analysis on cell lines – MH-S or MLE-12 cells were cultured at 37°C under humidified 5% CO_2_ in RPMI-1640 growth medium (Gibco, 72400047) or Dulbecco's modified Eagle medium (DMEM; Thermo Fisher Scientific, Waltham, MA), respectively, supplemented with 10% FBS (Gibco, 10438026) plus 1% penicillin/streptomycin (Gibco, 15140122). To examine the metabolic response in cell lines exposed to LPS and SCFAs overnight prior to metabolic analysis, we used the Agilent Seahorse XFe24 analyzer platform (Agilent Technologies, Inc.) according to the manufacturer's instructions. The Seahorse XF Cell Mito Stress Test was used to measure mitochondrial function by analyzing the oxygen consumption rate (OCR) of the cells. This plate-based live cell assay allows real-time monitoring of the OCR.

On the day before the assay, cultured cells were seeded onto the Seahorse XFe24 assay plate at an optimized density of 40,000 cells per well. After the cells had fully attached to the bottom of the plate, they were treated with LPS (10 ng/mL) and the SCFA propionate (C3, 0.1  mM or 5  mM) in RPMI 1640 complete culture medium and were left overnight. A sensor cartridge was hydrated with XF calibrant at 37°C in a non-CO_2_ incubator overnight.

On the day of the assay, an assay medium was prepared by supplementing XF RPMI medium with 1 mM pyruvate, 2 mM glutamine, and 10 mM glucose, ensuring a strict pH of 7.4. All the compounds used for the Mito Stress Test (Oligomycin, FCCP, and Rot/AA) were resuspended and diluted into appropriate working concentrations for the assay, and optimized concentrations of 1.5  μM for Oligomycin, 1  μM for FCCP, and 0.5  μM for Rot/AA were applied. The assay plate containing plated cells was carefully washed with fresh and warm assay medium, and then placed in a non-CO_2_ incubator for 1 h before the assay. Before beginning the assay, the Seahorse XF assay plate was removed from the 37°C non-CO_2_ incubator, the cells were examined under a microscope to confirm confluence, and then the cell assay plate was loaded to initiate the assay.

### Quantitative real-time reverse transcription polymerase chain reaction (RT-qPCR)

TaqMan-specific inventoried gene primers for glyceraldehyde 3-phosphate dehydrogenase (GAPDH), beta actin (Actb), ribosomal protein L19 (Rpl19), interleukin (IL)-6, IL-1β, IL-10, IL-18, NOD-like receptor family, pyrin domain containing (NLRP) 3, chemokine (C-X-C motif) ligand (CXCL) 1, and CXCL2 (Life Technologies, Carlsbad, CA) were used to measure the mRNA levels of these human or mouse genes in cells or lung tissue.

Lung tissue was homogenized (Tissue-Tearor – Biospec Products, Bartlesville, OK), and total RNA was isolated using TRIzol^®^ (Invitrogen – Thermo Fisher Scientific, Waltham MA) and RNAeasy Mini Kit (Qiagen) for RNA purification. A high-capacity RNA-to-cDNA reverse transcription kit (Life Technologies) was used with 1 μg messenger RNA per reaction. Quantitative real-time polymerase chain reaction was performed using TaqMan fast advance master mix and TaqMan gene expression assay (reagents) and QuantStudio^TM^ 6 and 7 Flex real-time PCR systems. Run method: polymerase chain reaction activation at 95°C for 20 s was followed by 40 cycles of 1 s at 95°C and 20 s at 60°C.

The average threshold cycle count (Ct) value of 2–3 technical replicates was used in all calculations. The average Ct values of the internal controls (GAPDH, beta actin, L19) were used to calculate the ΔCt values for the array samples. Data analysis was performed using the 2^−ΔΔCt^ method, and the data are presented as relative quantification (RQ).^40^^,^^41^

### SnATACseq on whole lung digests

Two groups of WT C57BL/6 mice (*n* = 4 each) were fed FR and FF diets for 2 weeks. Their lungs were harvested and subjected to digestion and isolation of single nuclei, followed by ATACseq performed by the UCSF ImmunoX Genomic CoLabs according to the manufacturer’s specifications (Single Cell ATAC, 10x Genomics, Pleasanton, CA).

### Sandwich enzyme-linked immunosorbant assay (ELISA)

Cytokines and chemokine concentrations in the cell culture supernatant, plasma and lung homogenate were determined using the mouse DuoSet ELISA kits for *in vitro* cell culture SNs and Quantikine ELISA kits for *in vivo* mouse plasma or lung homogenates (both R&D Systems, Minneapolis, MN). All the assays were performed according to the protocol supplied by the manufacturer. Standard curves were generated and used to determine the concentrations of cytokines and chemokines in the samples. The results are presented as mean ± SD.

### Correlation analyses (16S rRNA sequencing data, metabolomic data, immune/inflammatory markers)

The 16S rRNA sequencing and analyses were done by the UCSF BCMM Microbiome Core facility; the size of the sequenced paired-end libraries ranged from 5,976 bp to 285,86 4 bp, representing a total of over 15 million 151 bp reads. The reads were processed through Qiime2 (version 2020.8.0)^42^: low-quality reads and sequencing adaptors were removed using Cutadapt,^43^ and sequencing errors were corrected using Dada2^44^ using custom parameters (--*p*-trunc-len-f 150 --*p*-trunc-len-r 140). Taxonomic assignment of resulted ASVs was done using SILVA trained database (version 138-99)^45^ based on Scikit-learn’s naïve Bayes algorithm.^46^ For the subsequent analyses (except for alpha-diversity calculations), the abundance of each taxon present in a sample was normalized using the relative method to allow sample-to-sample comparison. The results were deep analyzed using the Phyloseq package (version 1.34.0)^47^ for the taxonomic and alpha diversity analyses. Statistical analyses were performed using rstatix (version 0.7),^48^ and figures were plotted using the ggplot2 package (version 3.3.5).^49^ Principal coordinate analyses (PCoA) were performed with the Vegan package (version 2.5–7)^50^ on the Bray‒Curtis dissimilarity matrices constructed from the relative abundance of ASVs. The communities that emerged were verified using a PERMANOVA test and the confidence intervals were plotted with 95% and 97% confidence limits, using the standard deviation method. Spearman correlation analyses were performed to determine associations between metabolites and microbiota species using the R package energy (version 1.7-8).

The Procrustes analysis is used to determine the strength of relationships between multivariate datasets. Procrustes analysis is based on matching corresponding data points from each of the two datasets. In particular, the Procrustes analysis was performed to determine the relationship between the Euclidean distances of protein/transcipt/M_data and the Bray‒Curtis distances of the microbiome. The length of the lines on the Procrustes plots indicates dissimilarity within-subject of the microbiome and metabolome. The goodness-of-fit is quantified by the Procrustes sum of squares (M-square), which represents the residual sum of squared distances between corresponding points in the two configurations. Lower M-square values indicate greater similarity between the matrices, whereas higher values reflect greater dissimilarity. Corr_Procrustes_rotation is a correlation value of the Procrustes rotation.

### Statistical analyses

Data in the figures are expressed as mean +/– SD. Data from *in vivo* studies comparing two conditions were analyzed using 2-tailed nonparametric Mann–Whitney analyses (data in Figures 2e, 2f, 4b, 4e, 4f, 4g, 6b, 6f, S1C, S2B, and S3C). Aseesa’s Stars analysis tool (www.aseesa.com) was used to analyze and generate principal analysis donut and scatter plots, and correlations.^51^ Additionally, linear trendlines, or an nth-degree polynomial trendline is shown if its goodness-of-fit is either 50% greater than or if it explains at least half of the variance not explained by the (*n*−1)th-degree polynomial. R^2^, r and *p* denote goodness-of-fit, Pearson’s correlation coefficient and significance of the correlation, respectively (data in Figures 3a, 3c, 3d, S4). Data from *in vitro* studies comparing two conditions were analyzed using standard Student’s t-test with equal SD to generate *P-*values (data in Figures 5a, 5b, 5c, 6e, 6h, 6i, and 6j). GraphPad Prism was used for statistical analysis (GraphPad Software, La Jolla, CA). *p-*values < 0.05 were considered significant. *P-*values are presented in the figures as follows: *< 0.05; **< 0.01; ***< 0.001; ****< 0.0001. *In vitro* experiments were repeated 2 or more times, and representative data are shown. The number of animals used in the *in vivo* experiments is described elsewhere within the methods section. One cage from the FF- > FR group (aka FR 1wk) with lung IR surgery was excluded because of the presence of *E. coli/Shigella* in the fecal microbiome composition indicating potential GI infection in this cage.

## Results

### FR diet results in metabolically reprogrammed BAL alveolar macrophages in uninjured lungs

We previously reported that (i) direct pulmonary administration of SCFA diminished lung immunity, (ii) that altering the gut microbiome with antibiotics enriched with SCFA-producing *Lachnospiraceae* species and reduced sterile lung injury,^27^ and (iii) that alveolar macrophages mediated this injury.^32^ Therefore, we investigated the impact of an FR diet, which is expected to naturally generate SCFAs after fiber fermentation by gut microbiota, on the transcriptional programming of alveolar macrophages (AM). We fed mice an FR diet containing 35% pectin for 2 weeks and used an FF diet (0% fiber) as our control (schematized in Figure 1a and detailed in Figure S1A). We chose pectin over inulin and other fiber-containing prebiotics for its known ability to reliably generate fecal SCFAs and based on previously published work.^9^^,^^24^^,^^52^ The higher fiber diets resulted in differences in stool and intestinal morphology but did not result in any differences in weights between the two groups (Figure S1B, C).

Figure 1.FR diet changes the gut microbiome composition and reprograms BAL AMs towards oxidative phosphorylation (pre-injury). (A) Experimental outline for FF (0% fiber) and FR (35% fiber) dietary intervention, followed by bronchoalveolar lavage and bulk RNAseq analysis. (B) Family-level changes in the gut microbiome comparing mice on FR and FF groups (after 2 weeks of dietary intervention) (left); weighted UniFrac beta diversity analysis (right). (C) Heat map of the differentially expressed genes between these two groups (left); volcano plot of the differentially expressed genes (FR/FF) (right). (D) Differential gene expression analysis using the MSigDB Hallmark reference databases shows enrichment of key pathways (OX-PHOS highlighted in white text) in the FR group compared to the FF group. *n* = 10 mice in each group. (E) IL-6 response in BAL AMs from mice on FR and FF diets after being challenged with LPS (10  and 100  ng/mL, left). *p-*values are represented comparing IL-6 in AM cells between FF and FR diet groups: *< 0.05; microscopic images (right) of cytospin preparations from AMs from FR (red box) and FF (blue box) mice. These images provide a visual representation of the AM morphology. ND: normal diet; FR: fiber rich; FF: fiber free; BAL: bronchoalveolar lavage; OX-PHOS: oxidative phosphorylation; AM: alveolar macrophage; LPS: lipopolysaccharide; IL-6: interleukin-6.

**Figure:**
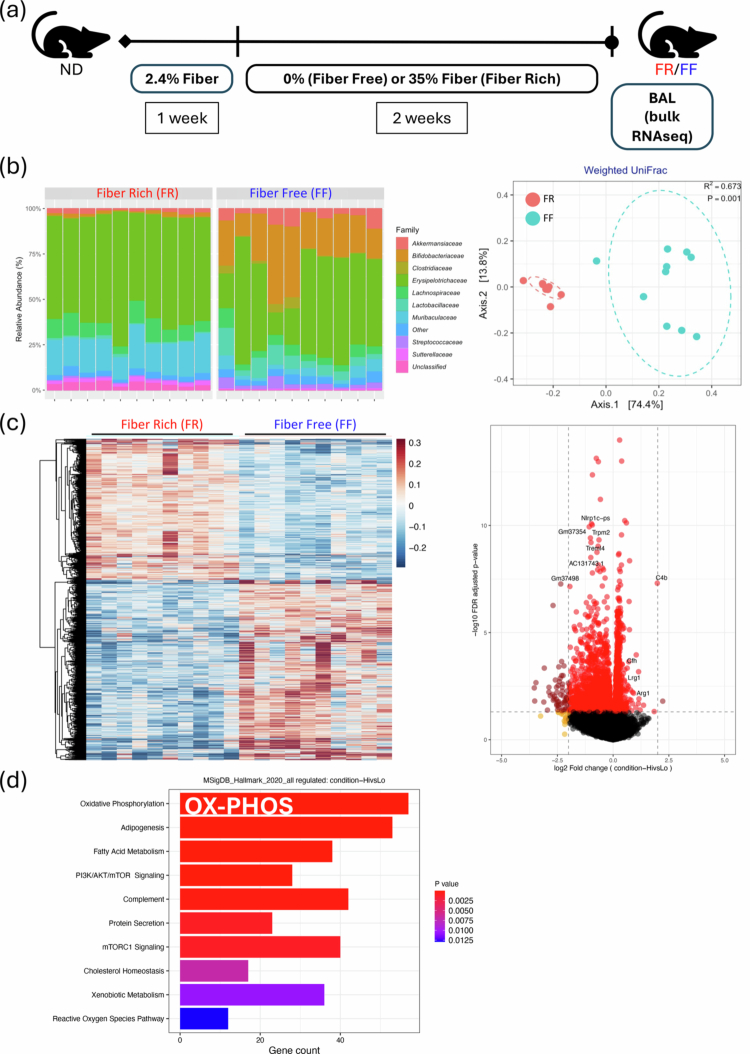

**Figure:**
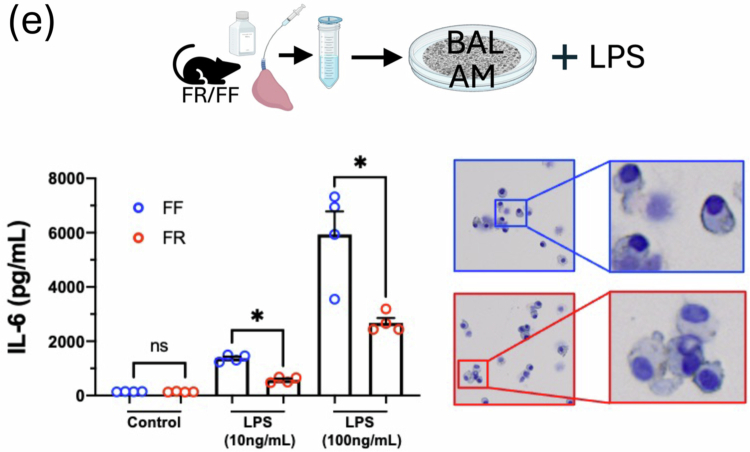

To confirm that our dietary intervention resulted in a change in the resident gut microbiota, we performed 16S rRNA sequencing of fecal contents and observed distinct taxa profiles between the two groups, which were clustered separately by beta diversity analyses, and had different enriched taxa (Figure 1b, left and right, and Figure S2A). After performing bulk RNAseq of BAL cells from these mice, we observed that airway cells (which were predominantly AMs) from mice that were exposed to FR diets for 2 weeks were strikingly different from those obtained from FF diet-fed mice (Figure 1c, left). Notably, our analyses did not reveal any meaningful differences in the transcript levels of AM (CD11c and SiglecF) or neutrophil markers (Ly6G and CD11b) expressed, alleviating concerns that these fiber diets might affect the number of AMs present in the mouse lungs or that the FF diet might cause baseline lung inflammation and accumulation of neutrophils (data not shown). In addition, we observed no evidence of differences in gut leakiness between these two groups (plasma LPB levels, Figure S2B). Gene transcript enrichment analysis (GSEA) revealed a handful of differentially expressed genes, most notably Arg1, that were enriched in the FR group (Figure 1c, right), suggesting that FR AMs may be skewed toward M2 (anti-inflammatory) and away from M1 (inflammatory) macrophage phenotypes. Pathway enrichment analyses also showed that oxidative phosphorylation (OX-PHOS) was the #1 (MSigDB) and #4 (KEGG) most highly enriched pathway in the FR group (Figure 1d and Table S1), which is consistent with M2 programming.^13^ GSEA also showed that the inflammatory response, complement, interferon alpha response, and interferon gamma response pathways were negatively enriched (Table S1).

### FR diet mice BAL alveolar macrophages are less responsive to LPS injury

To investigate the effect of dietary fiber content on alveolar macrophage (AM) responses to sterile LPS injury, BAL AMs (enriched by adherence plating) were stimulated with two doses of LPS (10 and 100 ng/mL). AMs from FF mice produced significantly higher IL-6 levels compared to FR AMs (Figure 1e, left). Cytospins were performed to image the BAL populations and the results did not show any morphological differences between the AMs (Figure 1e, right).

### FR diets result in distinct gut microbiota profiles with taxa enriched for SCFA production and increased transmission of SCFAs from gut to lung

Next, we subjected the mice that were exposed to FR (1 or 2 weeks) or FF (2 weeks) diets to lung IR injury (1 h left lung ischemia, 1 h reperfusion) (schematized in Figure 2a). As shown in Figure 2b, the gut microbiota profile was strongly influenced by the dietary change, with the FF diet resulting in a very distinct microbiota taxa profile from both 1- and 2-week-old FR diet-fed mice (Figure 2c, left) and both distinct from the baseline (*t* = 0, normal diet (ND) – 2.4% fiber) (Figure 2c, right), with the FR and FF groups well separated in beta-diversity analyses. Interestingly, the FF mice clustered closer to the baseline (t = 0) profile. We observed an expansion of the *Bacteriodetes* phyla and a strong reduction in the *Firmicutes:Bacterioides* ratio in the FR groups (Figure 2b and data not shown). The FF diet resulted in an enrichment of *Verrucomicrobiota* (including *Akkermansia*), in agreement with other reports.^53^

Figure 2.FR diet increases SCFA levels and blunts lung IR injury-related inflammatory responses with distinct microbiome regulation of IL-1β and IL-18. (A) Experimental outline for FF and FR dietary intervention, followed two weeks later by lung ischemia reperfusion (IR) injury. Different measurements at the start of the fiber diet intervention and after the IR injury are described in the table. (B) Family-level changes in the gut microbiome comparing mice on normal (2.4% fiber) chow (*t* = 0, prior to dietary intervention; *n* = 40), FF, and FR groups. (C) Beta diversity clustering of mouse groups showing FF vs. FR 1 week and FR 2 weeks (left) and comparing all three with starting (*t* = 0) microbiome on a normal diet (ND) (right). (D) Positively enriched taxa in FR group vs. baseline (*t* = 0 (ND)) and FF vs. baseline (left); taxa highlighted in yellow are common to both the FR 2wk and FF diets vs. baseline. The top 6 taxa uniquely enriched in the FR 2wk group and the major metabolites produced are listed with SCFAs and metabolites that can be easily converted to SCFAs highlighted in bold (right). (E) Heatmaps showing changes in SCFA levels in feces among the three dietary groups (left top). Comparison between dietary groups in portal blood, plasma, and right lung tissue (top right and bottom). *p*-values are represented by comparing FF vs FR 1wk and FF vs FR 2wk as follows: *< 0.05; **< 0.01; ***< 0.001; ****< 0.0001. (F) Post-IR injury inflammation: plasma IL-6 and lung tissue inflammatory markers measured in the normal diet (ND), FF, FR 1wk, and FR 2wk groups (top and middle left 6 panels). IL-6, CXCL-1, and CXCL-2 mRNA levels post-IR injury in the ND, FF and FR 2wk (bottom left three panels) groups. Summary of all plasma and lung tissue analytes measured and the level of significance for values different between FR and FF groups (right table). *p*-values: * < 0.05; ** < 0.01; *** < 0.001. (G) Volcano plot showing increased and decreased metabolites, and cytokine/chemokine in all tissue and fluid samples examined in the FR group versus FF. C2 in portal blood was the most increased measure and isovalerate in feces was the most decreased measure in the FR diet group. Other key significantly changed factors are highlighted in black boxes. The green lines denote significance cut-off levels (lighter to darker, *p* < 0.05, *p* < 0.01, and *p* < 0.001). ND: normal diet; FR: fiber rich; FF: fiber free; wk: week; AM: alveolar macrophage; LPS: lipopolysaccharide.

**Figure:**
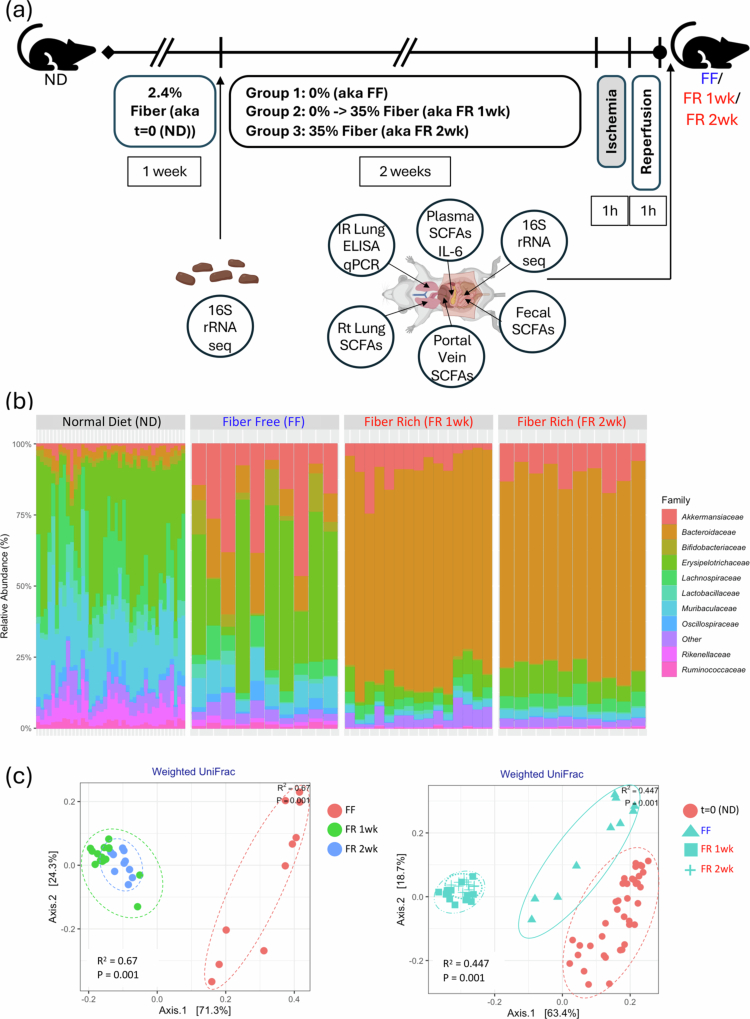

**Figure:**
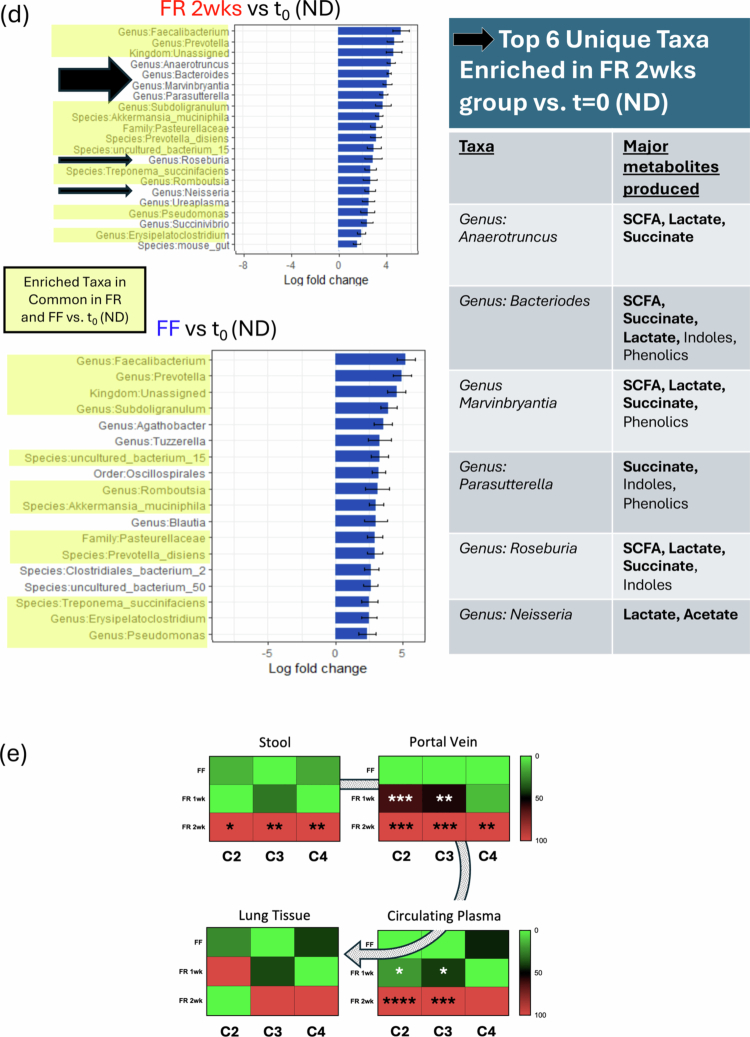

**Figure:**
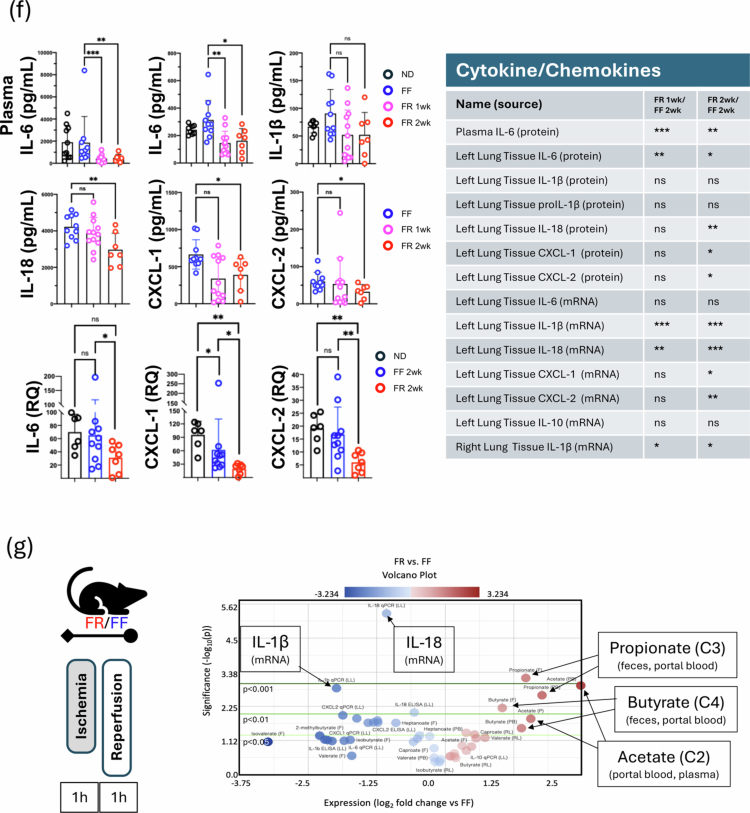

Analysis of the enriched bacteria in the FR 2wk group over baseline (*t* = 0) ND microbiota revealed unique taxa present at > 3-fold magnitude increase including members of the genera *Anaerotruncus, Bacteriodes, Marvinbryantia, Parasutterella, Roseburia, and Neisseria* (Figure 2d, left and also for all three diet groups vs ND, Figure S3A and compared to each other, Figure S3B). The top 6 enriched taxa were mostly strongly SCFA producers^1^^,^^8^^,^^29^^,^^54-56^ as might have been expected given the composition of the FR diet (Figure 2d, right). Other metabolites produced by these taxa included lactate and succinate, which are also known to be converted into SCFAs (butyrate and propionate, respectively) through bacterial cross-feeding.^57-61^

To confirm the presence of dietary fiber-dependent SCFAs after gut microbiota fermentation, we examined the fecal contents for SCFA levels and observed the expected increase in C2, C3, and C4 in the FR groups, especially in the 2 weeks FR diet group (Figure 2e, top left). We then followed the transit of SCFAs from the colonic contents (stool) to the portal vein, to the circulating plasma, and finally to the lung tissue (Figure 2e, clockwise from top left). The levels of SCFAs in lung tissue were elevated in the FR groups but not significantly different, and we hypothesized that this was due to SCFA consumption by injured lung tissue.

### FR diet results in reduced sterile lung IR injury with distinct regulation of IL-1β and IL-18 responses

We examined these mice on FF and FR diets for immediate early lung injury responses to lung IR injury. This early time point after IR injury (1** **h post-reperfusion) has been validated by our previous work and focuses on the first wave cytokine and chemokine release *prior* to the histopathological signs of lung injury (which peak at 3 h post-reperfusion and as we have previously reported, include edema, hyaline membrane casts, and large neutrophilic infiltration).^32^

We observed that FR diet mice had significantly reduced lung IR injury as measured by lower systemic and local IL-6 levels (compared to both FF and ND control mice). The levels of IL-1β and IL-18 in the lung tissue were also reduced. These findings were confirmed at the transcript level, with reduced levels of IL-6, CXCL-1 and CXCL-2 mRNA levels in injured lung tissue from the FR diet mice, particularly in the 2 weeks FR diet group (Figure 2f, left). A summary of all the metabolites and inflammatory markers that were differentially expressed in the FR groups (compared to the FF group) is shown in a table (Figure 2f, right) as well as in the volcano plot representation is shown in Figure 2g. Overall, C2 and C3 were the factors most elevated in the portal blood, feces and plasma, while lung IL-18 and IL-1β mRNA were the most significantly decreased (all measurements included in Figure S3C).

### Association of SCFA levels and gut microbiota with IL-1β and IL-18 injury responses after lung IR

Next, we performed multi-omic analyses of our combined multivariate datasets consisting of multiple measurements (gut bacterial 16S rRNA gene sequences, SCFA metabolomics, targeted transcriptomics, and targeted proteomics) from individual mice (on the 3 different diet regimens: FF, FR 1 week, FR 2 weeks) with the goal of uncovering correlations of interest between our measured factors.

We first examined the individual datasets from the three dietary intervention groups and the combined data from all three groups and despite the relatively small number of mice (*n* < 30) and the targeted nature of our inflammatory gene measurements, we observed that IL-1β mRNA levels negatively correlated at the phylum level with fecal Proteobacteria levels (*p* = 10^−6.4^) and IL-18 mRNA positively correlated with fecal Firmicutes levels (*p* = 10^−10.1^) (Figure 3a, top). In addition, lung IL-18 mRNA levels were negatively correlated with fecal C3 (propionate) levels (*p* = 10^−7^) (Figure 3a, bottom). Other significant correlations are shown in Table S2 and Figure S4.

Figure 3.Multi-omic analyses of inflammatory markers, metabolites, and the gut microbiota in the FR and FF diet groups post lung IR injury. (A) IL-1β levels in the left lung measured by qPCR correlated with fecal Proteobacteria levels, and IL-18 levels correlated with Firmicutes and propionate levels (clockwise from left). All three groups are included separately (FF - orange, FR 1wk - purple, and FR 2wk—blue lines) and combined (gray line). Individual r and *p* values for each group are shown with the gray line (all groups combined) *r-* and *p*-values magnified and reproduced in each graph as a gray inset box. (B) Procrustes test analyses results correlating gut microbiome 16S rRNA sequencing dataset with inflammatory transcriptomic/proteomic and SCFA metabolomic datasets. Significant data set correlations’ *p*-values are shown in bold (top left). A visual representation of Procrustes analyses is shown at top right (16S rRNA sequence dataset vs. lung tissue injury transcript qPCR dataset) and below with dotted black boxes denoting the combined FR 1wk and FR 2wk data showing the strongest correlations. Bottom row (left to right) – 16S rRNA sequence dataset vs. lung tissue injury protein ELISA dataset; 16S rRNA sequence dataset vs. stool SCFA metabolome dataset; 16S rRNA sequence dataset vs. portal blood SCFA metabolome dataset; 16S rRNA sequence dataset vs. plasma SCFA metabolome dataset; 16S rRNA sequence dataset vs. right lung SCFA metabolome dataset. (C) PCA Donut chart showing contributions of the five principal components accounting for 90+ % of FR group’s datasets variance and the 9 most correlated measures for each of the first 4 principal components are listed (top). The measures in red/blue text are positively/negatively correlated with the respective component. PC1 and PC2 are shown in a 2D PCA plot (bottom). FR: fiber rich; FF: fiber free; wk: week; PC: principal component; GF: germ-free; FMT: fecal microbiota transplant.

**Figure:**
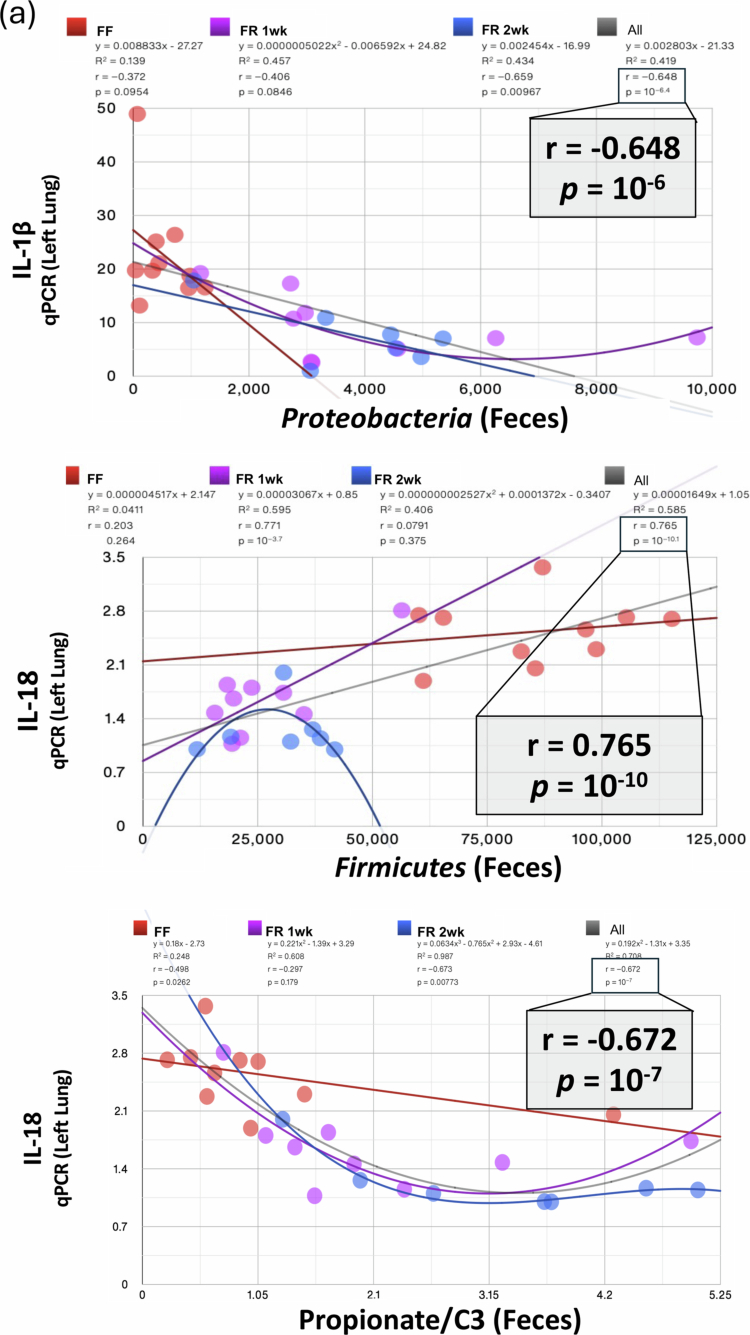

**Figure:**
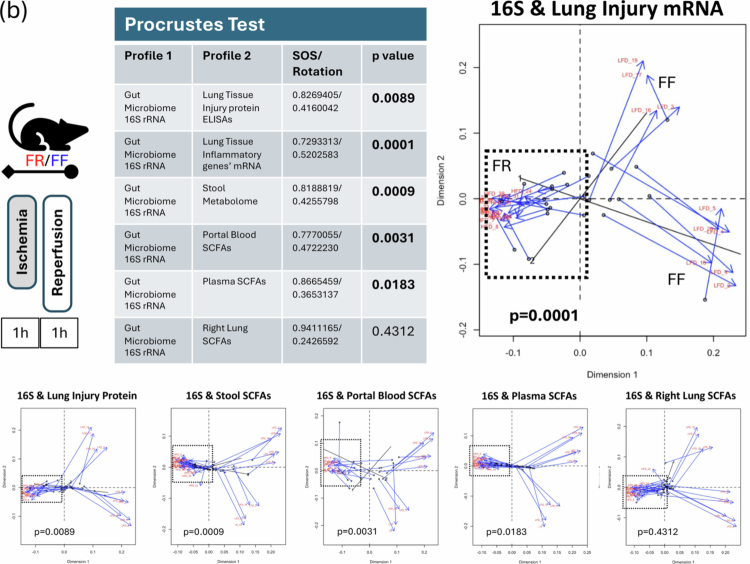

**Figure:**
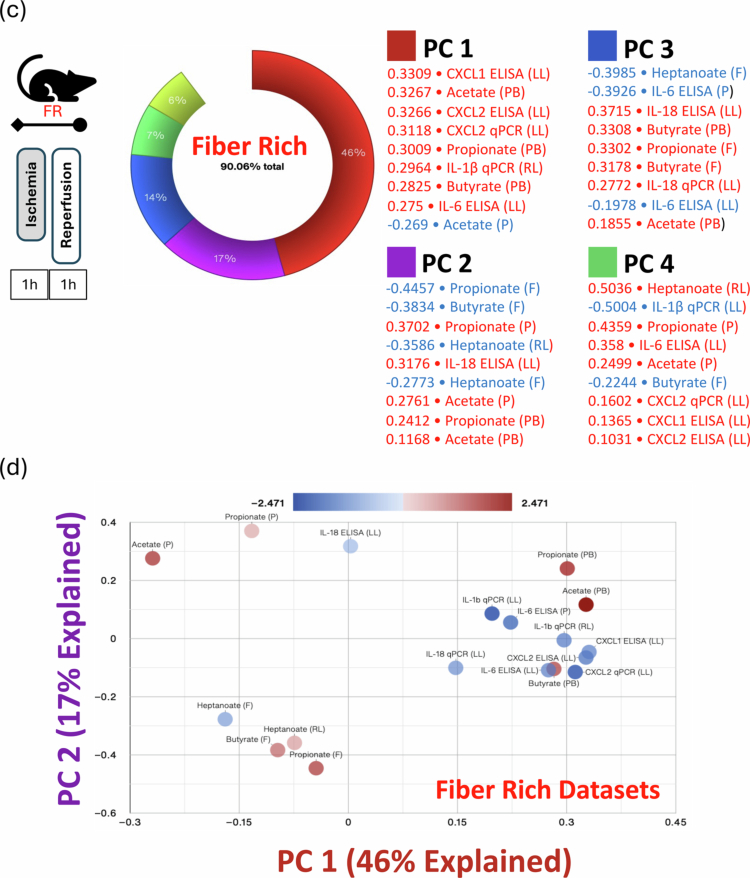

To further elucidate the relationships among these datasets, we applied Procrustes and Mantel tests (Figure 3b, top left and not shown). These analyses showed expected and significant correlations between the gut microbiome 16S rRNA gene sequenced dataset and the SCFA metabolomic datasets (consistent with gut microbiota being the primary source of SCFA production) (Figure 3b, bottom). However, we also detected highly significant correlations between the 16S rRNA sequencing dataset and the lung inflammatory marker transcriptomic dataset, as well as with the lung injury protein dataset (Figure 3b, top right and bottom left panels). The visual representation of the Procrustes analysis highlighted close relationships between the 16S rRNA dataset and the transcript dataset, as well as between the 16S rRNA dataset and the other datasets. These close relationships were particularly evident for the FR group datasets (shorter line distances on the Procrustes plots in Figure 3b and denoted by the dotted black boxes).

Principal component analysis (PCA) performed on the 2-week FR dataset focusing on metabolites (from all sample sources), protein and transcript data (from lung and plasma) revealed five modules or principal components that explained 90% of the variation observed in this dataset (Figure 3c). Modules combining SCFAs and inflammatory markers were observed, suggesting potential relationships between these factors within the gut‒lung axis (as shown in the PC1 vs PC2 plot, Figure 3d).

### Transfer of the fiber-driven lung injury phenotype with fecal microbiota transplantation (FMT) and the ability of the gut microbiota to produce propionate

Next, we wanted to determine the importance of the gut microbiome in driving the impact of dietary fiber on lung immunometabolic programming and injury responses. We performed FMTs in GF mice using colon contents from SPF mice fed FR or FF diets for 2 weeks. After gavaging the mice with these microbial communities and allowing the gut microbiome to colonize the intestinal systems of the GF mice, we subjected the two groups to lung injury (Figure 4a). We observed that the FMT from the FR group was able to suppress the inflammatory responses we observed compared to the FMT from the FF group in the directly injured left lung (Figure 4b). To confirm that the gavage FMT input matched the gut microbiota present 2 weeks later, we performed 16S sequencing (Figure 4c) and beta diversity analyses, which showed that the FR and FF FMT inputs clustered closely with the GF colonized with FR and FF FMTs (Figure 4d).

Figure 4.FR and FR phenotypes can be transferred with FMT and differentially affect the bacterial pneumonia course. (A) GF mice received FMT using FF and FR homogenized fecal content prior to lung injury. (B) Levels of inflammatory markers in the left lungs shown as measured by qPCR. (C) Family-level composition of the input FMT inoculum (left side for each of FR and FF) as well as the colonized gut microbiota after FMT (right side for each of FR and FF) by 16S rRNA sequencing. (D) Weighted UniFrac, Bray‒Curtis, and Canberra beta diversity analysis of input and stool microbiota (left to right). (E) GF mice received either FMT oral gavage inoculation with either WT *B.theta* or mut *B.theta* (del prop) for 3 weeks (1 gavage/week) and then subjected to lung IR injury. Levels of inflammatory markers in left lungs and plasma as measured by qPCR (top left two panels) and ELISA (remaining panels). (F) *S. pneumoniae* intranasal (IN) infection of ND, FF and FR diet-fed mice with body temperature (top left) and oxygen saturation/SpO2 (top right). *p*-values represent the significance values for the comparisons of the FR diet group against both the FF and ND groups. BAL IL-6 levels, BAL bacterial load, BAL total protein, and BAL total cells were also measured (bottom left to right). *p*-values: *< 0.05; ** < 0.01; *** < 0.001. (G) Regulation of IL-1β and IL-18 pre- and post-lung IR injury comparing the normal diet (ND) to the FF to FR (2 wk) diets. This figure includes collated data from multiple experiments using the 3 different diets + /– lung IR injury. *p-*values: *< 0.05; **< 0.01; ***< 0.001. FR: fiber rich; FF: fiber free; ND: normal diet; RQ: relative quantification; GF: germ-free; CFU: colony-forming units; FMT: fecal microbiota transplant; BAL: bronchoalveolar lavage; IR: ischemia reperfusion.

**Figure:**
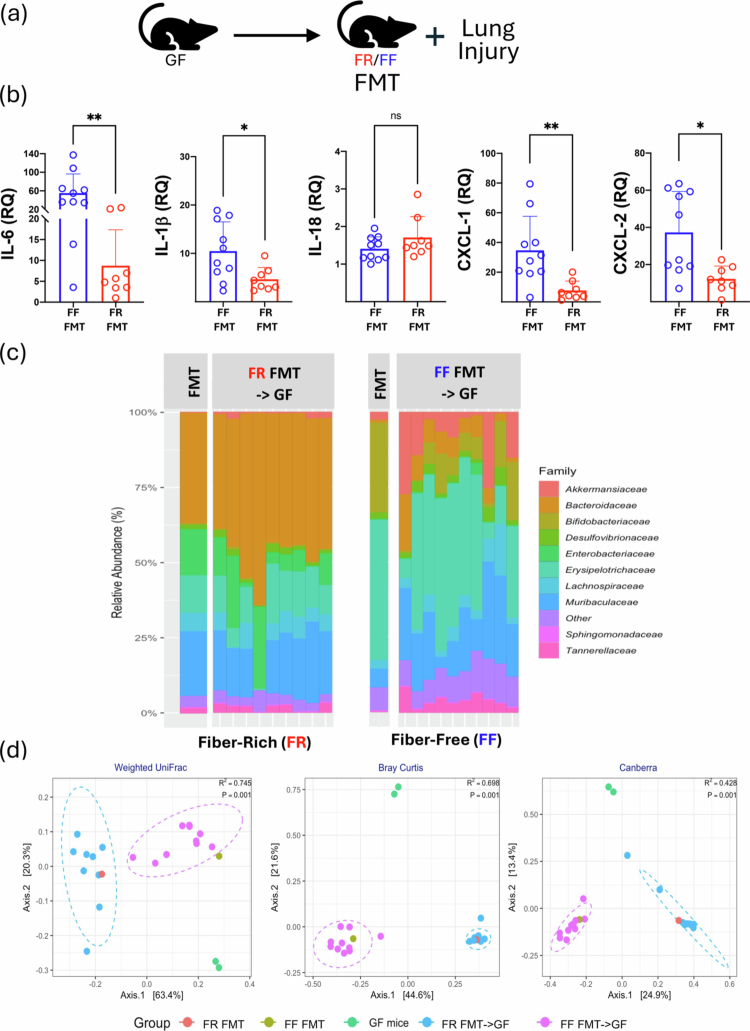

**Figure:**
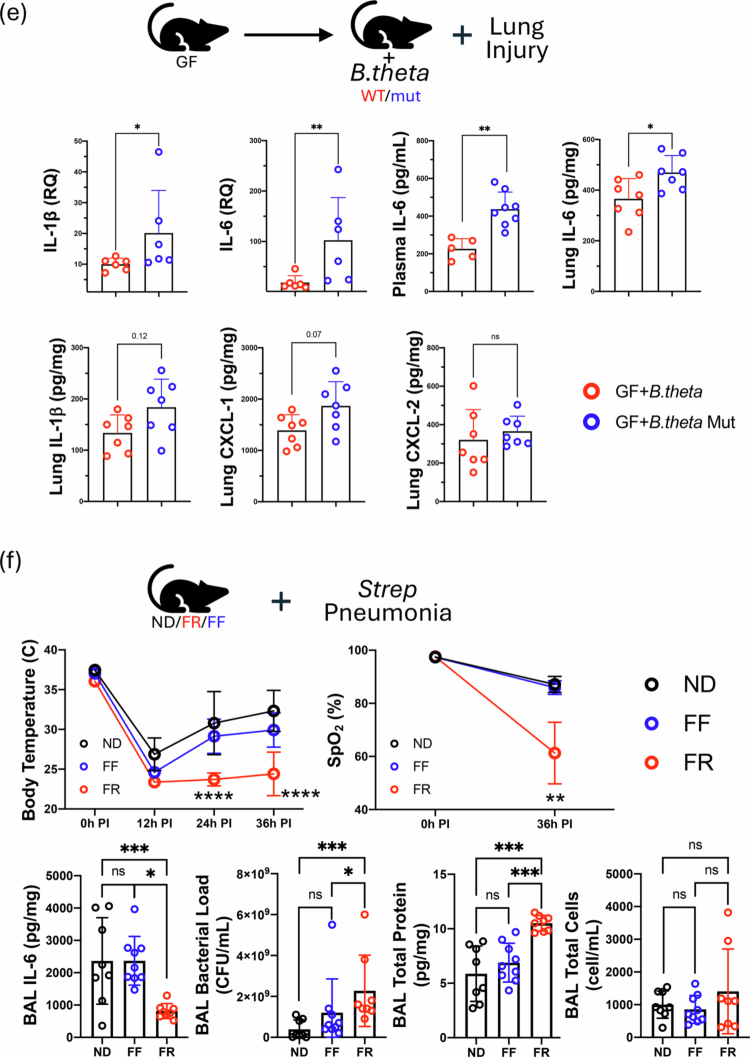

**Figure:**
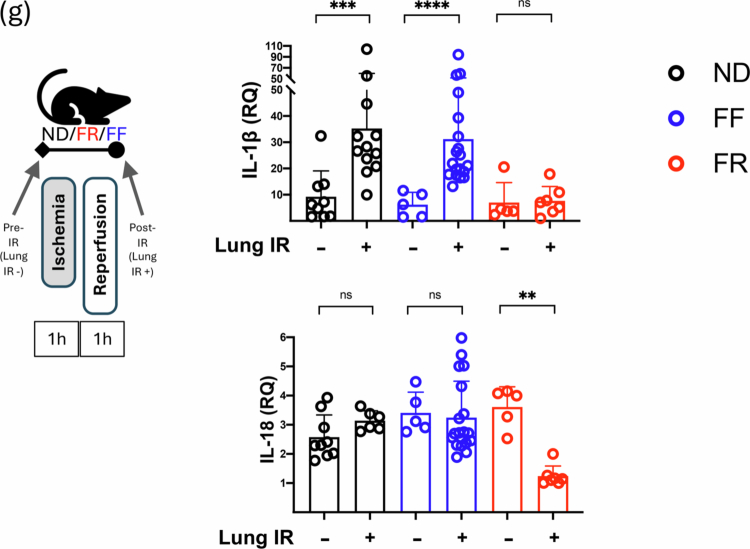

We confirmed the importance of propionate production in controlling lung IR injury by inoculating GF mice with a commonly prevalent propionate-producing gut microbe from the *Bacteriodes* genus, namely *B.thetaiotomicron* (aka *B. theta*)*.* We used either WT *B. theta* or mutant *B.theta* that are unable to produce propionate^35^ (strain information in Table S3). The mice colonized with the mutant *B. theta* had elevated lung injury markers post-IR injury (Figure 4e and data not shown) consistent with the hypothesis that the presence of propionate controls or reduces lung injury responses.

### FR diet influences bacterial pneumonia outcomes and associated lung damage

To investigate whether FR diets could positively or negatively affect host defense against lung infection, we used a bacterial pneumonia model (10^8^ CFU *S. pneumoniae* IN infection without any antibiotics) in mice after they were fed 2 weeks of FR, FF diets with a normal diet (ND) as a control. We observed that the FR group had significantly lower body temperature and oxygen saturation levels 36 h after pneumococcal infection, but there was no significant change in body weights post infection (Figure 4f, top and data not shown). Further analysis of these mice, revealed that FR mice had reduced BAL IL-6 levels, elevated BAL bacterial loads and increased BAL total protein without any difference in BAL cell number or composition (Figure 4f, bottom left to right and data not shown), or in other words, the FR mice had the combination of the least controlled infection and worst lung injury compared to FF and ND mice.

### Reprogramming of lung injury responses by FR diets does not occur at baseline via modulation of IL-1β and IL-18 resting levels

IL-1β and IL-18 are key early cytokines for the generation of lung injury under different contexts, including as we have reported – IL-1β specifically for lung IR injury,^18^^,^^21^ we delved deeper into the regulation of the mRNA levels of these two cytokines before and after IR injury (pre-IR and post-IR). We initially predicted that FR diets would reduce the levels of resting IL-1β mRNA within the lung prior to injury. Instead, we observed the opposite – baseline levels of IL-1β and IL-18 were essentially identical in uninjured ND, FF and FR mice. Strikingly, the mRNA levels of these cytokines were distinctly altered in the IR-exposed mice only 2 h only post-injury, with the expected IL-1β mRNA induction observed in the ND and FF groups but not in the FR group (Figure 4g, top). IL-18, on the other hand, was equally surprisingly suppressed 2 h post-injury in the FR group compared to the FF and ND groups (Figure 4d, right). Separately, our observations that the ND and FF groups had similar post-injury responses were supported by earlier observations that their other injury responses and microbiome profiles were more similar to each other than to the FR group (Figure 2f, left and Figure 2c, right).

### SCFAs regulate inflammatory responses and metabolic programming in alveolar macrophage in vitro

If the combination of a high-fiber diet, the production of SCFAs and the transit of SCFAs to the lungs result in the immunometabolic reprogramming of AMs from the M1 to M2 phenotypes, we wanted to examine whether this reprogramming could also occur *in vitro* in the presence of C3. We stimulated AMs (MH-S cell line) with LPS alone or in the presence of increasing C3 concentrations and observed the clear change from an M1 to M2 phenotype with inflammatory IL-6 levels decreasing while IL-10 levels increased (Figure 5a). GM-CSF levels were induced by LPS but remained elevated even in the presence or absence of C3 (data not shown).

We next examined whether SCFAs might affect the LPS-dependent induction of IL-1β and IL-18 mRNA, similar to what we observed after lung injury in vivo with FR diets. We observed that C3 (and C4) was able to blunt the induction of IL-1β and IL-18 (Figure 5b). Using an *in vitro* nutritional IR model, we also observed SCFA affecting the inflammatory IR responses in AMs, with C2, C3 and C4 able to blunt the secretion of IL-1β and C3 able to do the same for IL-18 (Figure 5c).

Figure 5.SCFAs can reprogram alveolar macrophage immune responses to LPS and IR *in vitro*. (A) Effect of co-treating with increasing amounts of C3 (0.2, 1, and 5  mM) on LPS (100  ng/mL) inflammatory responses in MH-S alveolar macrophages *in vitro*. IL-6, IL-10 and GM-CSF levels were measured by ELISA at 24 h (IL-6) and 48 h (IL-10). (B) The alveolar macrophage cell line (MH-S) was pre-treated with different doses of C2, C3, and C4 (0, 0.3, and 3 mM) for 4 h and then challenged overnight with LPS (200 ng/mL). IL-1β and IL-18 mRNA levels were measured by qPCR. (C) The alveolar macrophage cell line (MH-S) was pre-treated with a range of C2, C3, and C4 concentrations (0, 0.03, 0.1, 0.3, 1, and 3  mM) and then primed with LPS (200  ng/mL) in the presence of the same type and dose of SCFA prior to *in vitro* IR. IL-1β and IL-18 levels measured after 1 h reperfusion by ELISA. *p*-values: *< 0.05; **< 0.01; ***< 0.001. AM: alveolar macrophage; LPS: lipopolysaccharide; C3: propionate; C2: acetate; C4: butyrate; RQ: relative quantification; IR: ischemia reperfusion.

**Figure:**
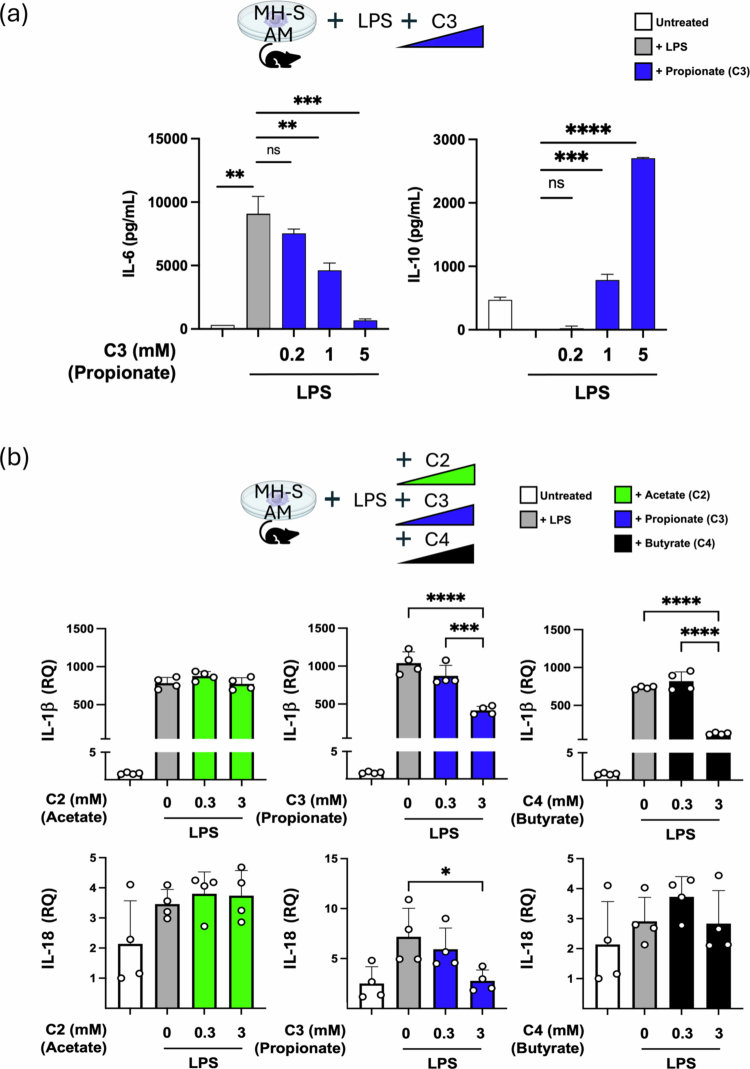

**Figure:**
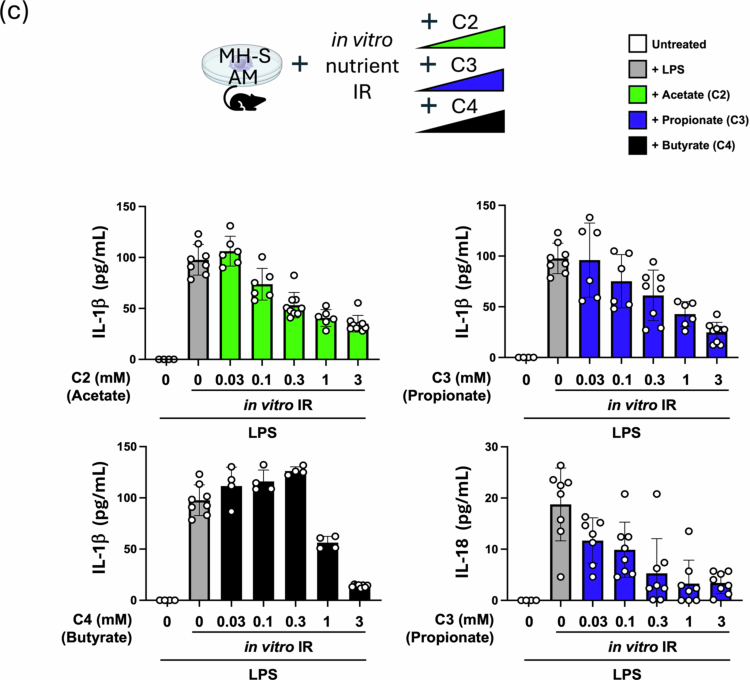

### SCFAs reprogram AMs via metabolic shifts and nutrient intake versus chromatin accessibility changes and FFAR signaling

SCFAs are thought to act on immune and non-immune cells via three distinct (and potentially overlapping) mechanisms, as shown in Figure 6a: they can signal via GPCRs (with FFAR2 and FFAR3 being 2 primary SCFA receptors); they can serve as an energy source and thereby affect cellular metabolism; and they can act as HDAC inhibitors by modifying inflammatory and other gene loci. To better understand the mechanistic basis for how SCFAs might affect AM immunometabolism and the shift from the M1 phenotype to the M2 phenotype, we performed experiments to test the contribution of each of these 3 mechanisms.

Figure 6.Propionate (C3) specifically alters AM immunometabolic programming independent of FFAR signaling and chromatin remodeling. (A) Three possible mechanisms for SCFAs affecting AM immunometabolic programming. Alveolar macrophage image from Biorender^TM^_._ (B) WT, FFAR2 and FFAR3KO BAL AMs response to *ex vivo* nutritional IR in the presence of 3  mM C3. IL-1β and IL-18 levels were measured 1 h after *ex vivo* reperfusion by ELISA. (C) ATACseq of lung cell populations after 2 weeks of FR and FF diet. Transcription start-site (TSS) enrichment comparison between the two groups (left); AM population differences in chromatin accessible regions of the genome by chromosome number (right). The blue line represents *p* = 0.1, and the red line represents *p* = 0.01. (D) t-SNE comparison of the calculated frequency of lung cell populations in FR and FF diet mice (right). Significantly different frequencies of cell clusters are denoted with an asterisk (*). (E) MH-S cells were pre-treated with C3 (1  mM) or medium for 24  h, followed by exposure to LPS (10 ng/mL) with or without co-treatment with C3 (1  mM) for 24  h. IL-6 levels in the supernatant were measured by ELISA. (F) Propionate (10  mg/kg) was administered IP 1.5 h prior to the onset of *in vivo* lung IR injury. Following IR injury, IL-6 levels in circulating plasma were measured by ELISA. (G) The oxygen consumption rate (OCR) as a measure of oxidative phosphorylation was performed on precision cut lung slices (PCLS) in the presence of 100  ng/mL LPS and indicated C3 concentrations (0.1 and 5  mM) for 18–24 h assessed using the Seahorse Mito Stress Test. The line graph illustrates dynamic changes in OCR. (H) Bar graphs represent key metabolic parameters, including basal respiration, maximal respiration, spare respiratory capacity, and ATP-coupled respiration for PCLS treated with LPS and C3. (I) Seahorse metabolic measurements on alveolar macrophages (MH-S cell line) and (J) alveolar type 2 cells (MLE-12 cell line) treated *in vitro* with LPS (10  ng/mL) and indicated C3 concentrations (0.1 and 5  mM) for 18–24 h. *p-*values are represented as follows in the figures: *< 0.05; **< 0.01; ***< 0.001; ****< 0.0001. All experiments were repeated at least twice and representative data are shown. FFAR: free fatty acid receptor; HDAC: histone deacetylase; WT: wild-type; KO: knockout; C3: propionate; IR: ischemia reperfusion; FR: fiber rich; FF: fiber free; ATAC: Assay for Transposase-Accessible Chromatin; TSS: transcriptional start site; bp: base pairs; AM: alveolar macrophage; LPS: lipopolysaccharide; ND: normal diet; PCLS: precision-cut lung slices; OCR: oxygen consumption rate; ATP: adenosine triphosphate; AT2: alveolar type 2 epithelial cells.

**Figure:**
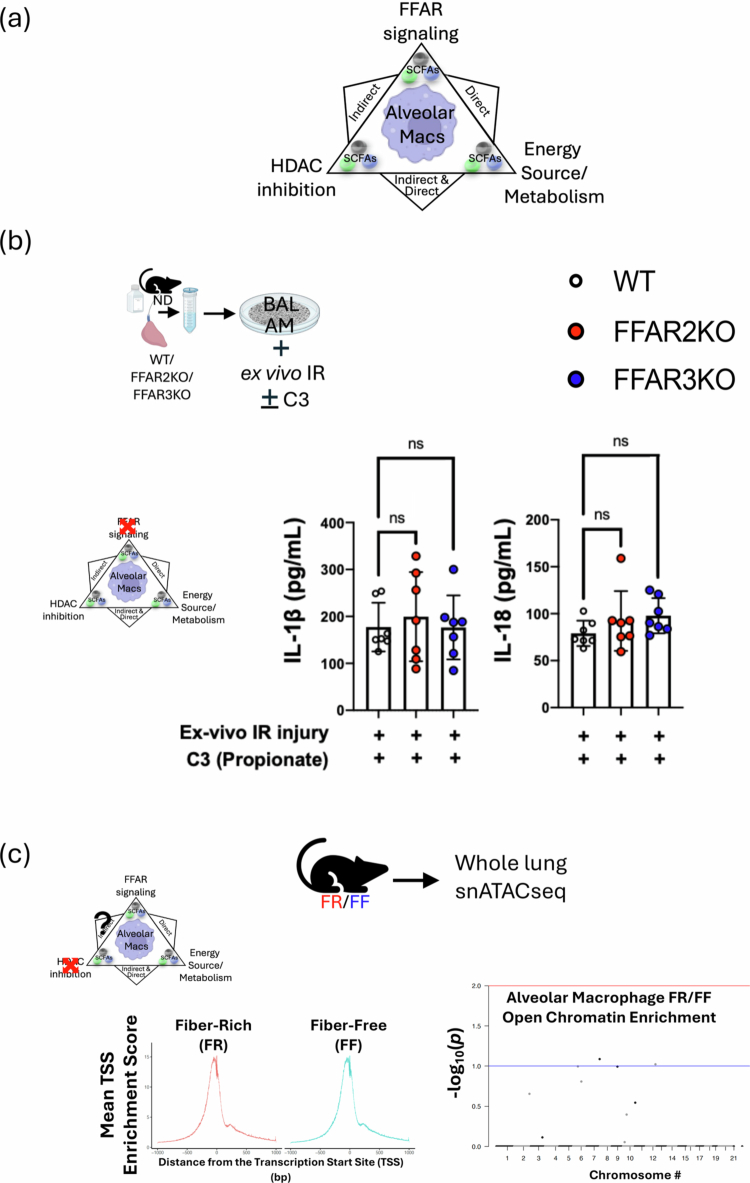

**Figure:**
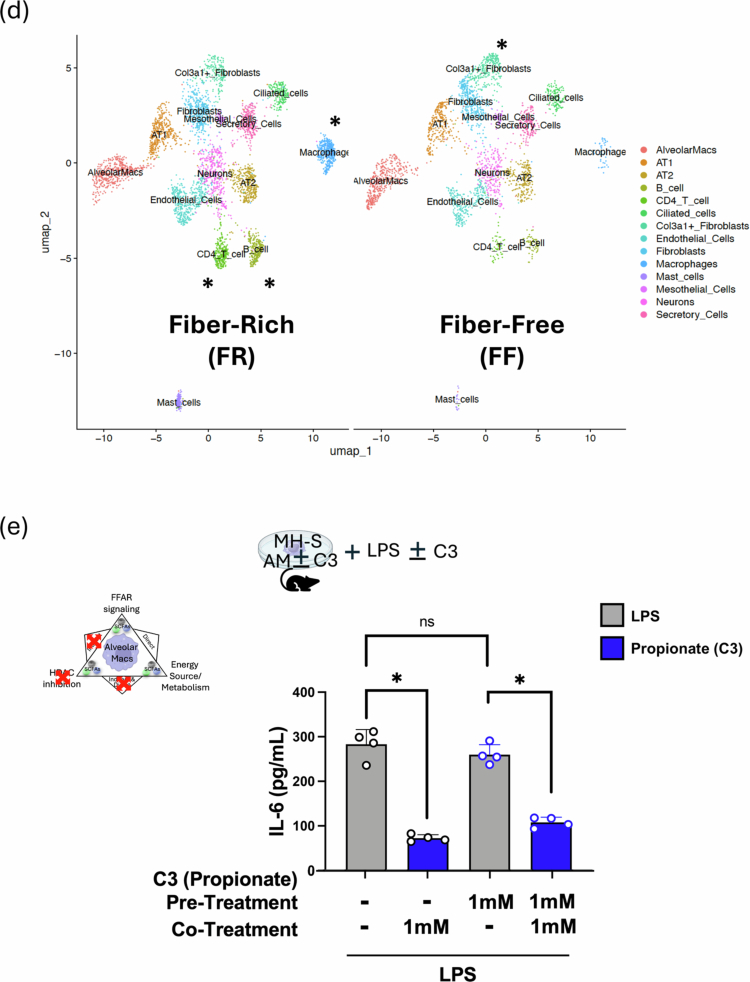

**Figure:**
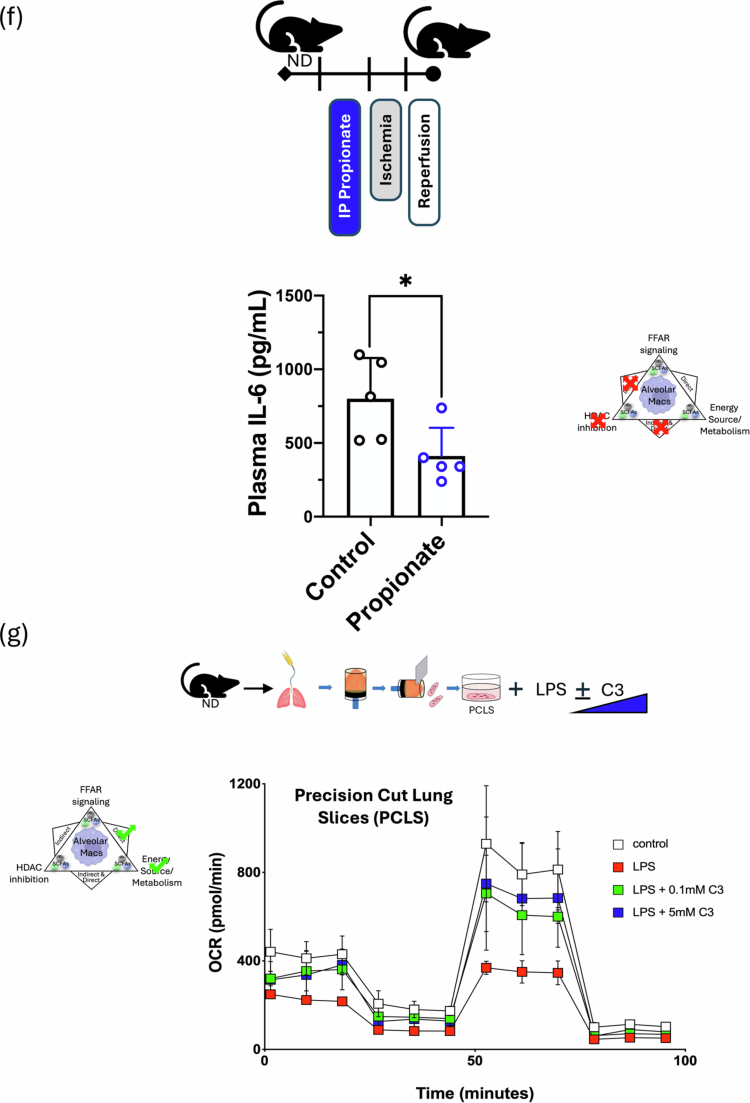

**Figure:**
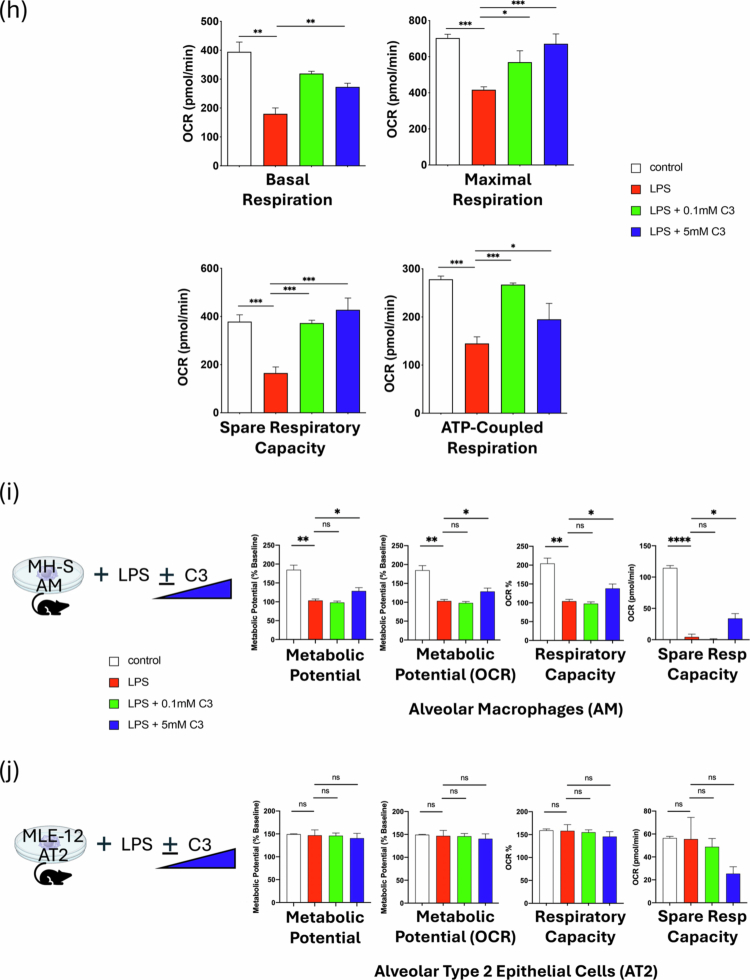

First, we examined the effects of FFAR2 and FFAR3 by collecting BAL AMs from WT, FFAR2KO and FFAR3KO mice and subjecting them to *ex vivo* IR injury in the presence of C3. As shown in Figure 6b, the inability of AMs to signal through FFAR2 and FFAR3 had no effect on their secreted levels of IL-1β and IL-18 in the presence of high-dose propionate (3 mM C3), suggesting that the C3-influenced inflammatory phenotype was not mediated by these receptors.

Next, we performed single nuclei (sn) ATACseq on whole lung tissue from mice (*n* = 4) exposed to FR and FF diets for 2 weeks. We observed expected changes in the gut microbiota (Figure S5), but we failed to detect any major differences in transcriptional start site frequencies (TSS) (Figure 6c, left) or any major genes of interest in different open versus closed chromatin states between the FR and FF groups in all lung cell types (Figure S6) and specifically in AMs, our cell population of interest (Figure 6c, right). We also did not observe any major changes in the lung populations (Figure 6d) between the FR and FF groups; however, there were statistically significant differences in the frequencies of non-AM macrophages (5x increase in FR), CD4^+^ T cells (3x increase in FR), B cells (3x increase in FR) and Col3a + fibroblasts (3x decrease in FR) that reached statistical significance (Table S4). Even among these cell populations, there were no differences in the chromatin accessibility of the IL-1β and IL-18 loci and few, if any, significantly different chromatin accessibility loci in general (Table S4E,F).

### Pre-treatment with propionate (C3) alone did not attenuate LPS-induced inflammatory responses in alveolar macrophages in vitro

To determine when the presence of SCFAs during lung injury was essential for regulating pulmonary inflammatory responses, the MH-S AM cell line was pre-treated with C3 (0 or 1  mM) for 24 h, then exposed to LPS (10 ng/ml) with or without co-treatment with C3 (1 mM) for an additional 24 h. Co-treatment with C3 during LPS exposure significantly reduced IL-6 levels in the supernatants, as determined by ELISA, whereas pre-treatment with C3 alone did not produce this effect (Figure 6e), suggesting that C3 presence was required during the injury period and that this mechanism was unlikely to involve chromatin remodeling via HDACi.

### Rapid effect of systemic propionate on lung IR injury responses in vivo.

To further test the ability of propionate to influence injury responses just by its presence during the injury period, we administered propionate IP immediately before (~1.5 h) to induce lung IR injury *in vivo*. In this context, we were able to observe a clear and significant reduction in lung injury responses (Figure 6f). This result argues against SCFA and fiber diet reprogramming of the bone marrow being required for the effects observed in attenuating lung injury. Similarly, it argues against changes in resident lung cell populations, as well as longer-term reprogramming of resident lung immune cells being required for the ability of propionate to affect lung injury responses.

### Propionate metabolically reprograms lung tissue and AMs in the context of LPS injury.

Our earlier published work showed that *in vitro* exposure of AM cell line (MH-S) to LPS skewed AMs towards glycolysis and this was reversed back to OX-PHOS by the presence of propionate (C3).^21^ To examine these effects in lung tissue *in situ*, we investigated whether C3 could affect the metabolism of lung tissue using *ex vivo* in precision cut lung slices (PCLS). LPS exposure in PCLS shifted metabolism from baseline OX-PHOS to favor glycolysis and the presence of C3 was able to reverse this shift back towards OX-PHOS (Figure 6g). C3 was able to partially or fully restore basal and maximal respiration, ATP-coupled respiration, and spare respiratory capacity in PCLS (Figure 6h). To delve deeper into which cell type might be driving the PCLS metabolic responses, we compared these effects on AM vs. AT2 cells *in vitro*. PCLS metabolism in the presence of C3 was similar to Ams (Figure 6i). In contrast, AT2 cells (MLE-12 cell line) were much less metabolically active and were not significantly altered metabolically under the same conditions (Figure 6j). Overall, the effects of C3 on lung tissue most closely mirrored those observed with isolated AMs.

## Discussion

The main conclusions of the present study are that gut microbial metabolites reprogram alveolar macrophages downstream of dietary fiber intervention and alter inflammatory and host defense lung immune responses. These conclusions are based on the following experimental evidence. First, we tested the effects of a FR diet on alveolar macrophages (AMs) and lung injury responses and compared these effects against a FF diet. Mice fed the FR diet exhibited distinct gut microbiota profiles and increased production of SCFAs secondary to fiber fermentation. Notably, AMs from FR-fed mice showed transcriptional changes, suggesting a shift toward an anti-inflammatory M2 phenotype with enriched transcription of genes involved in oxidative phosphorylation, suggesting metabolic reprogramming. In terms of lung injury, mice on the FR diet experienced significantly reduced lung IR injury as evidenced by lower levels of inflammatory cytokines such as IL-6, IL-1β, and IL-18, particularly after two weeks on the diet. This reduction in cytokines was attributed to the modulatory effects of SCFAs produced from the FR diet, suggesting a protective role against lung injury. Further multi-omic analyses, integrating 16S rRNA gene sequencing data, metabolomic and transcriptomic data, revealed correlations between SCFAs, gut microbiota profiles, and lung inflammatory markers, supporting the role of the gut‒lung axis in regulating lung injury. Furthermore, FMT from FR diet-fed mice to GF mice replicated the reduced inflammatory response, underscoring the role of gut microbiota in mediating diet-induced changes in lung inflammation. However, extreme fiber diets impaired host defense against bacterial pneumonia, suggesting a complex balance between dietary fiber, gut microbiota, and immune responses in the lung.

Our previous work has shown that the lung immunity can be influenced by the composition of the gut microbiome and that LPS and SCFAs can be transferred from the gut to the lung.^21^^,^^62^ Furthermore, our *in vitro* studies have shown that propionate can either prime or suppress alveolar macrophage inflammatory responses depending on the levels present and that enrichment of propionate-producing bacteria correlated with reduced lung IR inflammation.^27^ The importance of lung IR inflammation was also demonstrated by the fact that disruption of NLRP3 inflammasome-mediated IL-1β production or sensing was associated with worse bacterial clearance as part of a superimposed pneumonia.^18^ Others have also demonstrated the importance of the gut microbiota in fighting lung infections^63^^,^^64^ [and reviewed in,^65^] and high pectin fiber (as well as SCFAs directly) has been observed to alter lung adaptive immunity and bone remodeling.^9^^,^^24^

Recent work has reported an alteration in type 2 inflammation following high-fiber diet with worse allergic lung responses and improved parasite clearance in mice.^52^ This paper implicated ILC2s, IL-33, cholic acid, and the farnesoid X receptor (FXR) as driving the eosinophilia observed in their system. Interestingly, this group used inulin as their fiber source, but did not find significant elevations in fecal propionate and butyrate levels in their study. Other reports have shown that different fiber sources (inulin vs. pectin vs. psyllium) may have different effects on gut immunity with some fiber sources being beneficial and others being detrimental depending on the injury model.^66^ We used pectin as the fiber source based on reports showing strong effects on lung adaptive immune responses and bone pathophysiology^9^^,^^24^ and clearly observed increased SCFA levels in feces, portal blood and circulating plasma. Our work has also revealed some important differences in the regulation of IL-1β and IL-18, with only IL-1β being dependent on the presence of microbiota (also reported by others,^67^), while both IL-1β and IL-18 in the lung appear to be modulated by the presence of C2, C3, C7, and possibly other metabolites. Our focus on alveolar macrophages as the key cell regulating lung immune and metabolic tone is largely based on our previous work^21^ but is also supported by work from other groups focusing on alveolar macrophages in IR-induced lung injury,^68-70^ and the gut microbiota programming of alveolar macrophages in the context of respiratory virus infection.^71^ Metabolic reprogramming of alveolar macrophages and epithelia has also been observed both locally by pathogens and through TLR4 engagement.^72^^,^^73^

Our study begins to fill important gaps in our understanding of how the gut microbiome influences lung injury responses through the gut‒lung axis. Our findings that specific bacterial taxa can regulate the lung immunometabolic tone through the SCFAs they produce have important implications for human health, disease treatment, and prevention strategies. We can now begin to explore the effects of specific bacteria or consortia that alter lung injury responses downstream of metabolic reprogramming. Moreover, the ability of high-fiber diets to shape the gut microbiome, compared to fecal microbiome transplantation (FMT) in the clinic, makes dietary interventions very attractive as potentially powerful and simple tools to modulate lung injury responses, especially in the context of elective surgery. The direct administration of SCFAs to alter lung immunity and other organ responses has already been demonstrated by us and others,^9^^,^^27^ and the broad therapeutic applicability of SCFAs as supplements or for the ingestion of SCFA-rich foods needs to be further explored. Overall, compared to FMT and direct administration of SCFA (via pure compounds or foods rich in them), the use of dietary fiber to enrich the gut microbiome for bacteria capable of fiber fermentation may be holistic, simpler, and perhaps preferable. Specific diets (not just based on fiber) could be envisioned to shape organ health, resilience, and immunity in both health, prior to injury, and in disease.

Reducing inflammatory responses after sterile lung IR injury may be beneficial in clinical scenarios such as organ transplantation, reperfusion after pulmonary embolism, or even hypovolemic trauma. However, much remains to be learned about the effects of a high-fiber diet and reduced lung immunometabolic tone on other common lung injuries and diseases, including pneumonia, asthma, and pulmonary fibrosis. Other groups have reported increased viral and bacterial killing by macrophages in the presence of specific SCFAs.^74^^,^^75^ Our results suggest that extremely high-fiber diets decrease the ability of lung AMs and other cells to fight infection and subsequently cause worsened lung damage. Our previous work has also shown that suppressing inflammation through the interruption of inflammatory signaling pathways also worsened pneumonia caused by *E. coli* and *S. aureus* lung infections.^18^ While it is possible that strong suppression of inflammation is detrimental in the presence of an untreated infection, our *in vitro* data show that propionate does not indiscriminately suppress all immune responses, and perhaps at the appropriate balanced levels, may instead create an overall healthy state for alveolar macrophages and the lung as a whole.

As discussed earlier, a limitation of our study is the use of a single source of fiber (pectin). Additionally, other metabolites in addition to SCFAs could also be important, such as lactate, primary and secondary bile acids, tryptophan derivatives, lipids, vitamins, etc. It is plausible that SCFAs could interact with any of the other classes of gut- and diet-derived metabolites and factors to influence lung immunometabolic tone. These interactions need to be investigated in a systematic and unbiased manner. Our future work will further investigate gut microbiome metabolic pathways enriched with high-fiber diets and hopefully identify other metabolites that might be important in mediating the effects of dietary fiber. Our work cannot rule out the importance of enriched non-AM cells in FR mouse lungs or the possibility, however, of rapid changes in chromatin accessibility occurring within 2 h of lung injury initiation. Additionally, how lung-resident cells utilize SCFAs as energy sources in the context of injury to direct their immunometabolic programming away from pro-inflammatory responses requires further detailed study.

The main conclusions of this study are that a high-fiber diet leads to beneficial reprogramming of alveolar macrophages towards an anti-inflammatory state and reduces sterile lung injury via the production of SCFAs, although it may impair defense against bacterial infections, highlighting the complex interplay between diet, the gut microbiota, and lung health. Mechanistically, we hypothesize that gut-derived SCFAs that are transmitted to the lungs can provide metabolic feedstock that immunometabolically reprograms AMs towards an M2 phenotype, by favoring oxidative phosphorylation over glycolysis, especially in the context of lung injury. Specifically, for the field of sterile lung injury, these findings have broad implications for the use of dietary interventions prior to general surgery, lung transplantation, and other anticipated events involving lung injury. Further understanding of specific bacteria that interact with and manipulate lung immunometabolic tone may allow the identification of resilient patient populations as well as at-risk populations that may benefit from targeted dietary interventions (prebiotics), FMT with lung-protective taxa or combined synbiotic approaches. The use of specific metabolites to directly manipulate the system may also have a place in emergency situations where short-term manipulation to enhance or suppress lung immune responses may be in the best interest of the patient. Overall, the use of dietary interventions to holistically modulate lung function and immunometabolic tone may lead to novel therapeutic approaches.

## Supplementary Material

Supplementary material GM Supplementary figures revision v2.0_DM.pdf

## Data Availability

The authors confirm that the data supporting the findings of this study are available within the article and its supplementary materials. Raw data were generated at UCSF Microbiome and Genomics cores and University of Michigan Metabolomics core are available from the corresponding author [AP] on request.

## Department/Institution to which the work is attributed

Department of Anesthesia and Perioperative Care, University of California San Francisco and San Francisco General Hospital, San Francisco, California.

## Meetings at which portions of this work have been presented

American Society of Anesthesiologists (ASA) Annual meeting, October 2021, San Diego; American Thoracic Society (ATS) Annual meeting, May 2023, Washington, DC; Cell Symposia: Infection biology in the age of the microbiome, June 2023; Inserm Workshop 272: Les microbiotes – de l’exploration à l’application thérapeutique, May 2023; Lung Development, Injury and Repair GRC, August 2023.

## Abbreviations

SCFA: short-chain fatty acid
MCFA: medium-chain fatty acid
BCFA: branch-chain fatty acid
C2: acetate
C3: propionate
C4: butyrate
iC4: isobutyrate
2-me-C4: 2-methyl butyrate
C5: valerate
iC5: isovalerate
C6: caproate
C7: heptanoate
IR: ischemia reperfusion
FR: fiber-rich
FF: fiber-free
GF: germ-free
SPF: specific pathogen free
FMT: fecal microbiota transplant
