# Supplementary material for: Gut microbiome-derived propionate reprograms alveolar macrophages metabolically and regulates lung injury responses in mice
Source: Gut Microbes. 2025 Dec 30;18(1):2606486. doi: 10.1080/19490976.2025.2606486 (PMC12758369; doi:10.1080/19490976.2025.2606486)
Supplement: Supplementary material — GM Supplementary figures revision v2.0_DM.pdf [file KGMI_A_2606486_SM7576.pdf]

Figure S1A

Composition of diets used in this study

Product #	Normal diet		Fiber free (FF) diet		Fiber rich (FR) diet	
	PicoLab Rodent Diet 20 (5058)		Modified AIN-93G/No Cellulose(5GCX)		Mod TestDiet® 57W5 w/ 30% Pectin, Blue (5Z6R)	
	gm%	kcal%	gm%	kcal%	gm%	kcal%
**Protein**	21.8	23.217	18.3	17.900	18.0	26.900
**Fat (ether extract)**	9.0	21.566	7.1	15.600	7.0	23.600
**Carbohydrates**	51.8	55.217	68.2	66.600	33.2	49.600
**Total fiber**	**2.4**	0	**0.0**	0	**35.0**	0
**Kcal/gm**		100		100		100
**Energy(kcal/g)**	4.63		4.10		2.68	

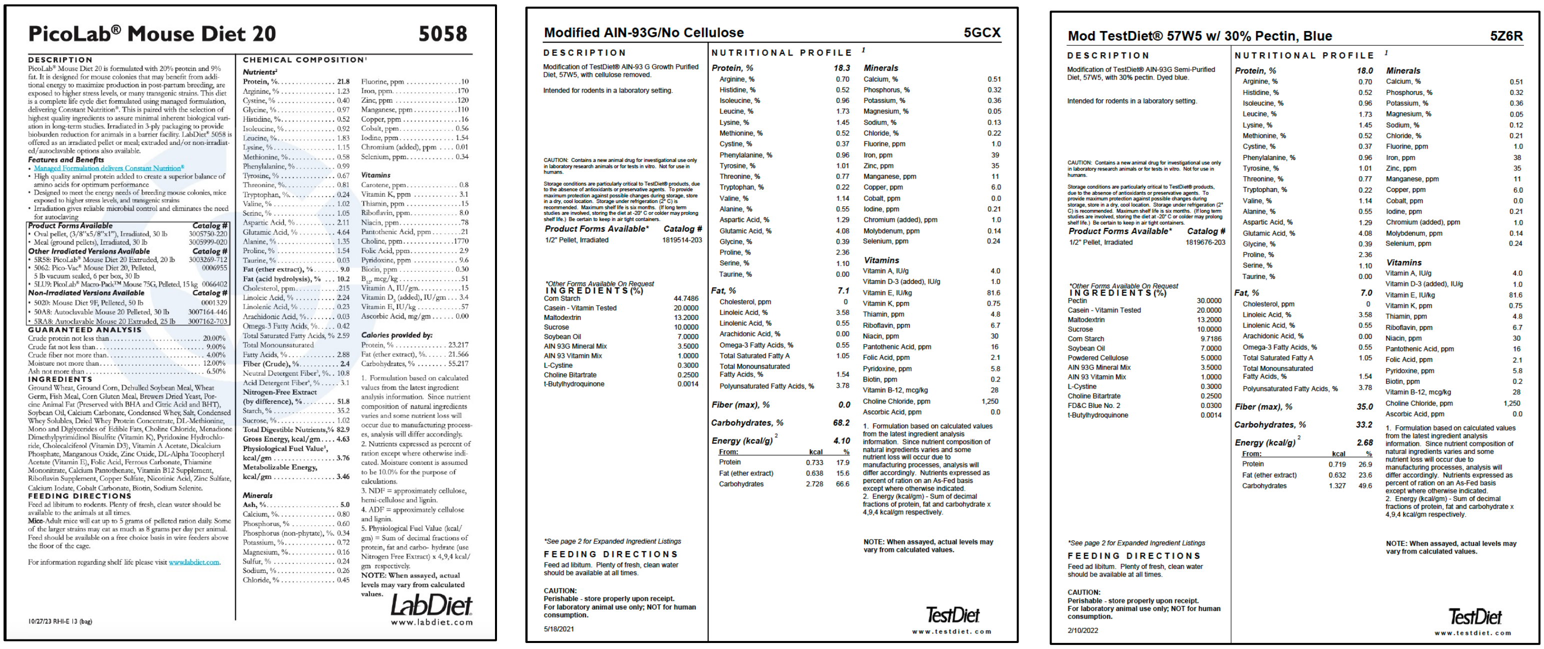

Figure S1B

FF Diet FR Diet

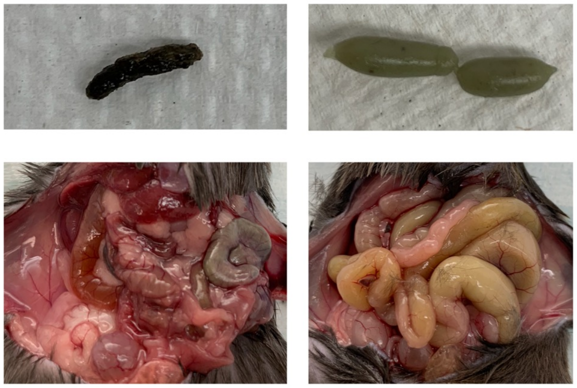

Figure S1C

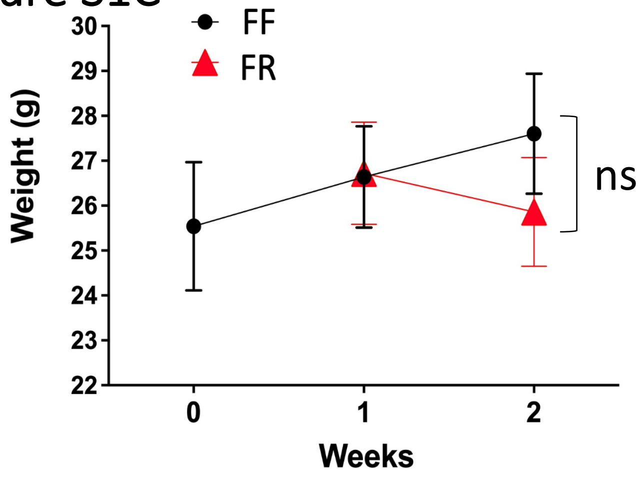

Supplemental Figure S1: A. Composition of diets used in study (fiber levels highlighted in bold). B. Representative image of stool and colon morphology in FF vs FR groups. C. weight changes over diet course (n=10 (FF) and 10 (FR); ns by 2-tailed nonparametric Mann–Whitney analysis).

Figure S2A1 (cont'd)

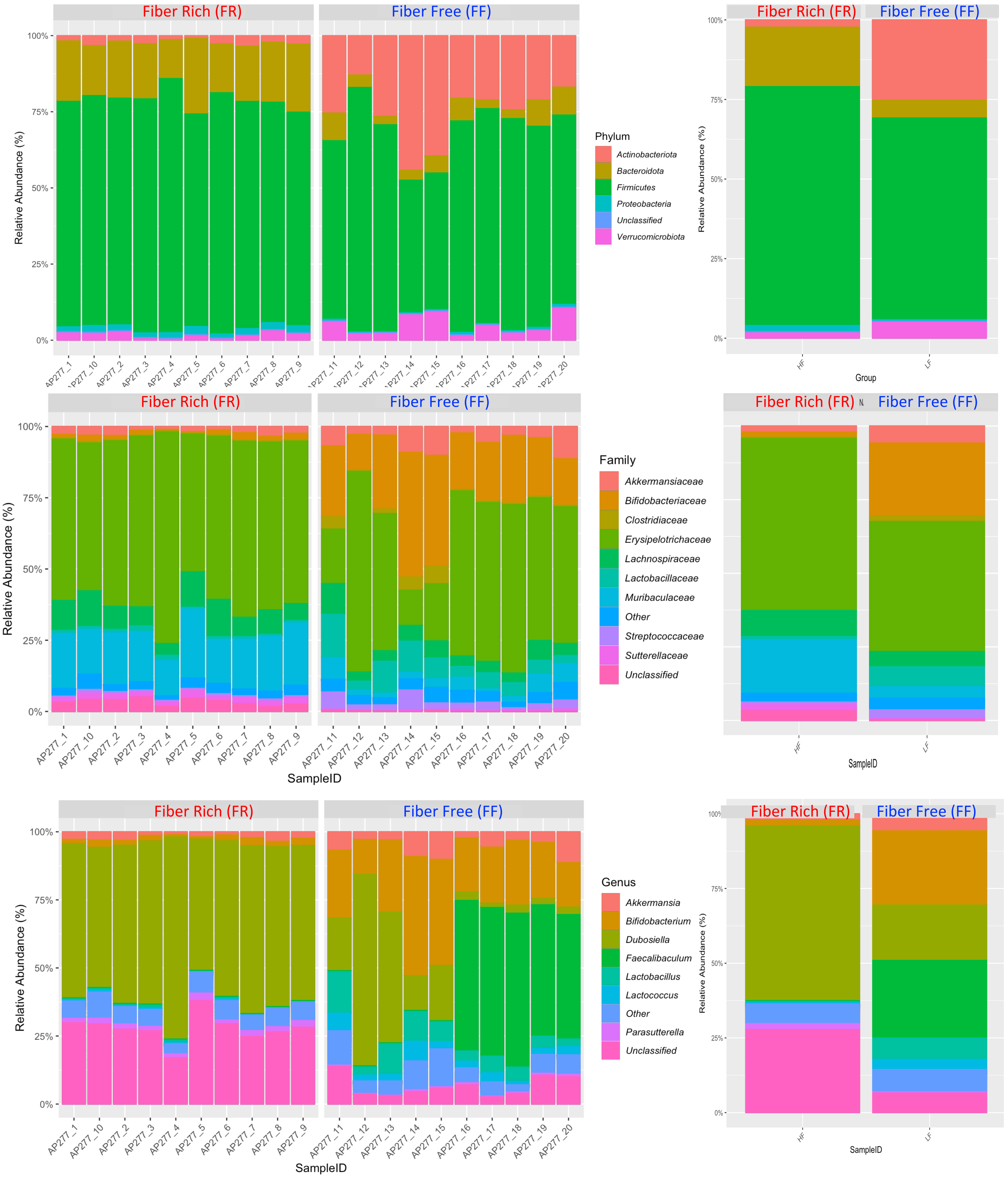

Supplemental Figure S2A1: 16S sequencing of FR and FF mice. Phylum, Family and Genus level comparison and statistical analyses – left: individual mice and right: averaged for each group.

Figure S2A2 (cont'd)

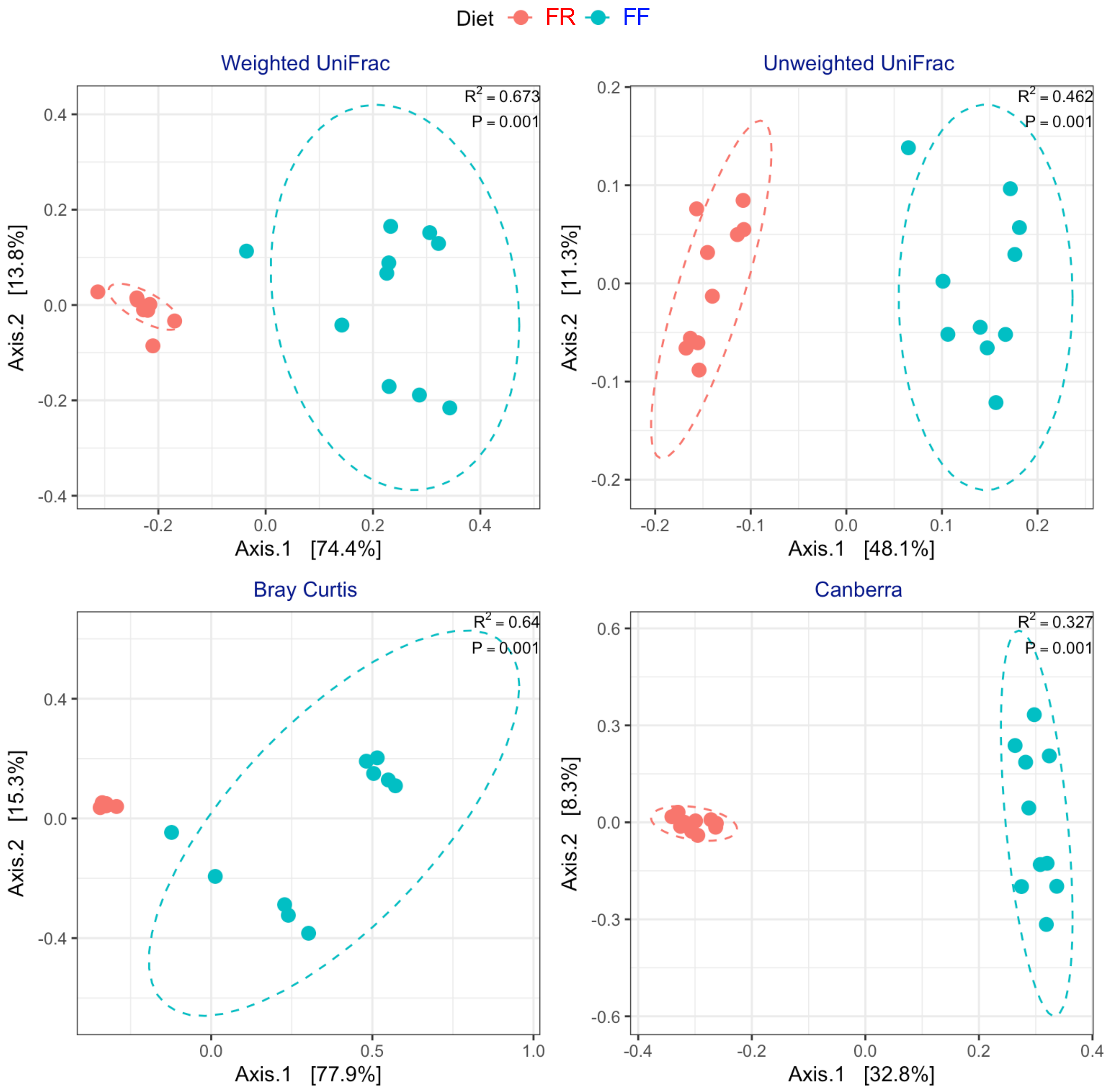

Supplemental Figure S2A2: 16S sequencing of FR and FF mice. Beta Diversity analyses and Taxonomic Abundance Table.

Figure S2A (cont'd)

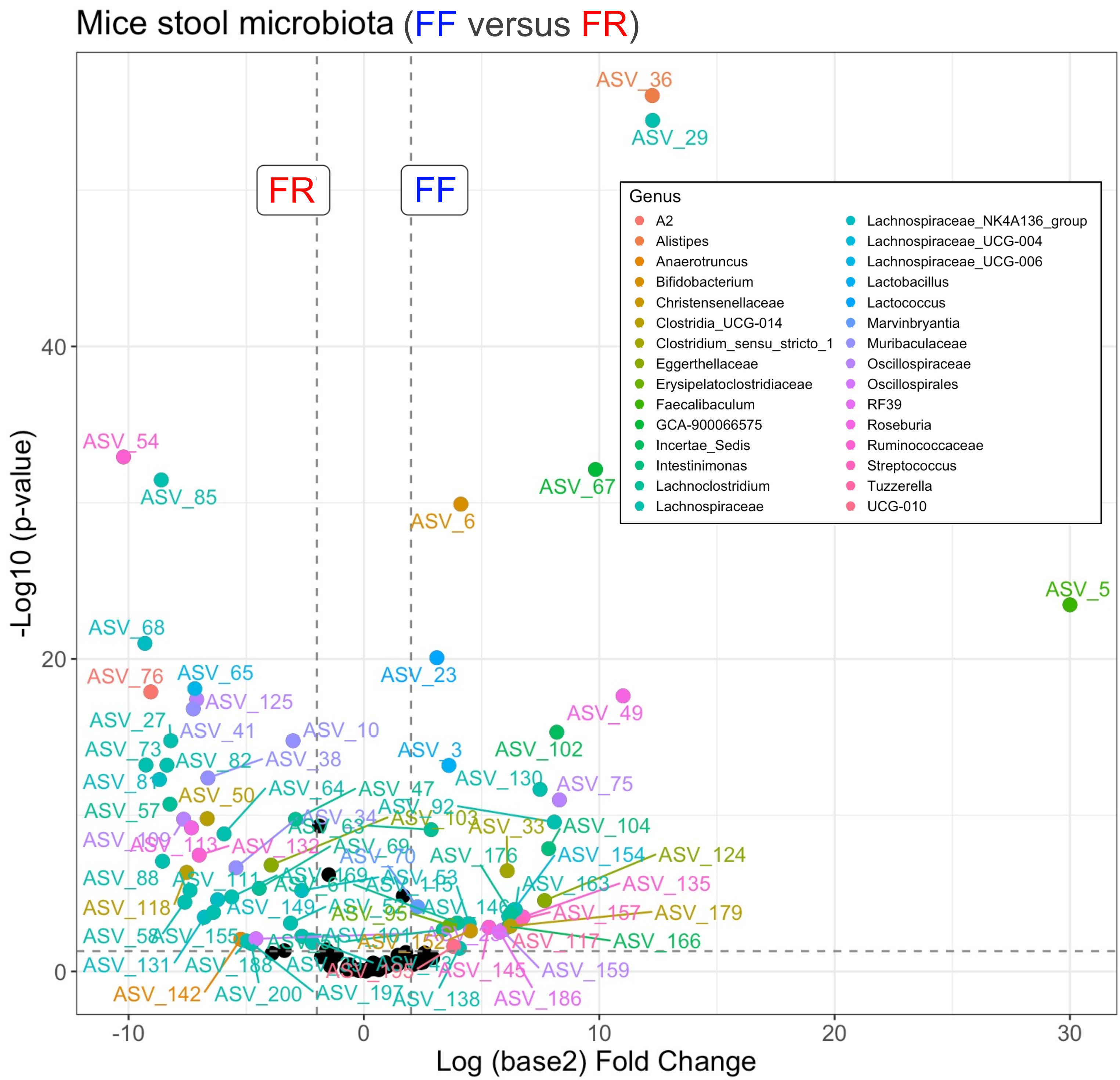

Supplemental Figure S2A3: 16S sequencing of FR and FF mice. Differential taxa analysis and heatmap showing abundance distribution of identified significant ASVs (FF vs. FR). The ASV/taxa that are significantly (padj < 0.05 & log2FC >±2) different between groups (if any) are labeled in the volcano plot. Additionally, taxa that are significantly different between groups (if any) are also shown in the inserted readout table.

Figure S2A (cont'd)

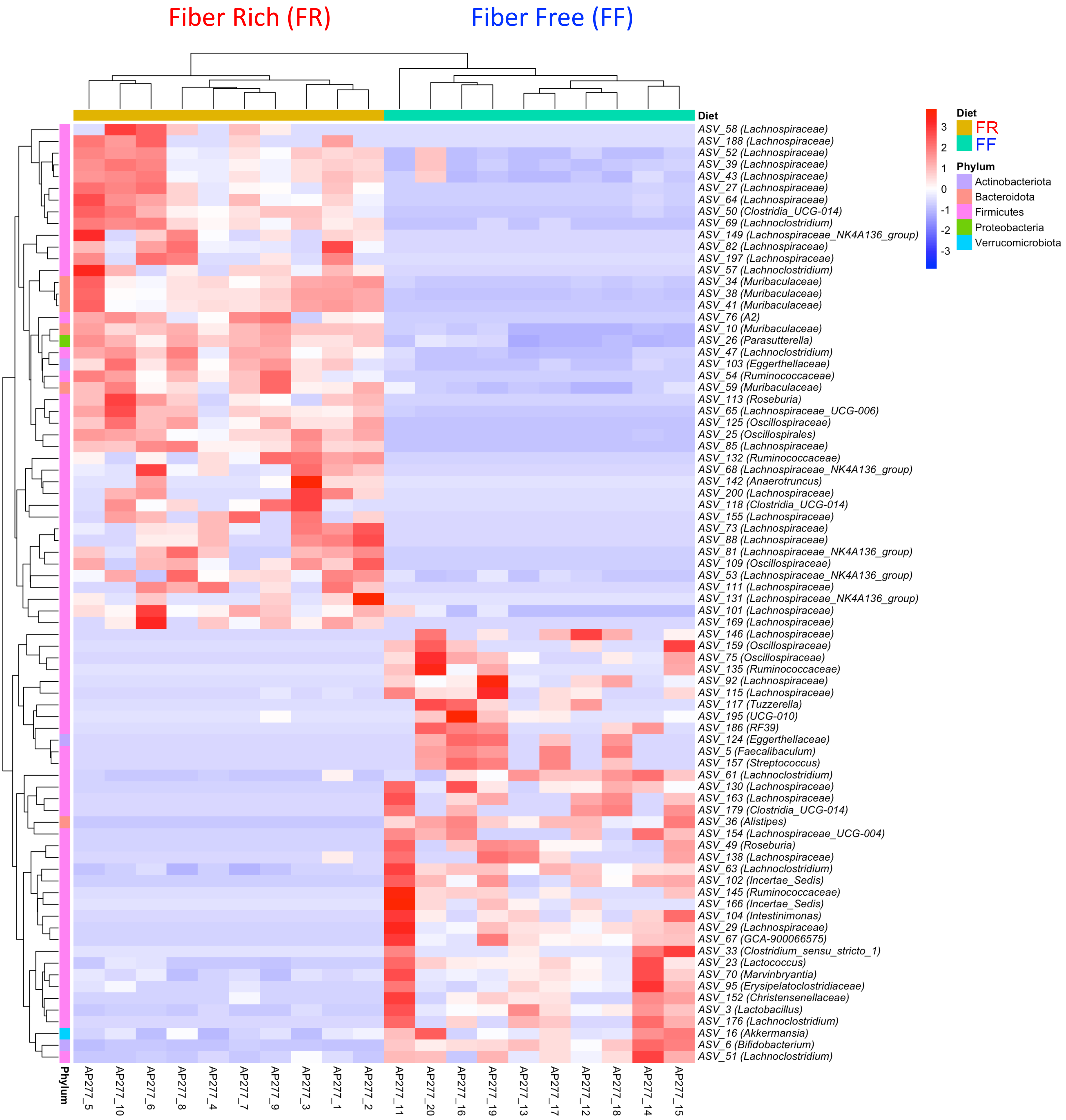

Supplemental Figure S2A: 16S sequencing of FR and FF mice. Differential taxa analysis and heatmap showing abundance distribution of identified significant ASVs (FF vs. FR). The ASV/taxa that are significantly (padj < 0.05 & log2FC >±2) different between groups (if any) are labeled in the volcano plot.

Figure S2B

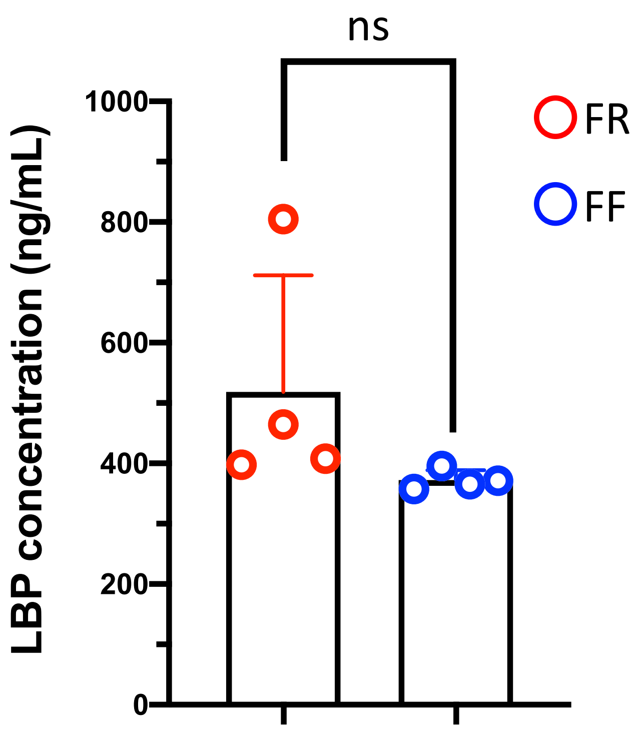

Supplemental Figure S2: No difference in Gut leakiness between FR and FF mice. The LBP concentrations in systemic plasma collected from mice subjected to fiber-rich diet (FR), or fiber-free diet (FF).

Supplemental Table S1

MSigDB: OX-PHOS genes enriched in FR/FF diet mice BAL bulk RNAseq:
COX7B;ACADVL;ACAA2;COX4I1;ETFA;PHB2;TOMM22;NNT;UQCRFS1;ACADM;DLAT;IDH3A;BCKDHA;CPT1A;ECH1;SDHC;SDHD;ATP1B1;COX6B1;POR;NDUFS8;UQCRC1;SUCLG1;VDAC1;SLC25A5;SLC25A4;NDUFB8;ECHS1;RETSAT;COX15;NDUFB5;TIMM13;ETFDH;DLST;UQCR11;ACAT1;PRDX3;LDHA;CYB5R3;POLR2F;SLC25A20;NDUFV2;NDUFV1;ATP6V1C1;NDUFA9;NDUFA5;MDH2;NDUFA4;IDH3G;GOT2;NDUFA1;IMMT;COX6C;CS;GLUD1;ALDH6A1;UQCRQ

KEGG: OX-PHOS genes genes enriched in FR/FF diet mice BAL bulk RNAseq:
NDUFA13;COX7B;NDUFB8;NDUFA11;COX15;NDUFB5;COX4I1;ATP5A1;ATP5K;ATP5C1;ATP5J;UQCR11;ATP5H;ATP5G3;ATP5O;ATP5G1;ATP5B;ATP5E;UQCRFS1;NDUFV3;NDUFV2;NDUFV1;ATP6V1C1;LHPP;NDUFA9;NDUFA5;NDUFA4;NDUFA1;SDHC;SDHD;COX6C;COX6B1;NDUFS8;UQCRQ;UQCRC1;ATP6V0D2

Top 15 GSEA (all three databases – KEGG, MSigDB, GO): adjusted $p<0.05$ pathways enriched in FR/FF diet mice BAL bulk RNAseq – OX-PHOS pathways highlighted in yellow

Enrichment Category	Term	Overlap	P value	Adjusted P value	Odds Ratio	Combined Score	Genes
GO_Biological_Process_2021	cotranslational protein targeting to membrane (GO:0006613)	40/94	1.4790619756545 3E-13	6.91461473618491E-10	5.403086419753 09	159.619049484342	RPL5;RPL30;TRAM1;RPL3;RPL32;RPL10;RPL34;RPL12;RPL36A;RPL10A;RPL8;RPS4X;RPL7A;SEC61A1;RPS16;RPS15A;RPS19;SRP72;RPS18;RPL36;RPL14;RPS3;RPLP2;SSR1;RPL15;RPS27A;SIL1;RPL17;RPS13;RPS12;SEC63;RPL19;RPL21;SSR2;RPS6;RPL35A;RPSA;RPS25;RPL27;RPL29
GO_Biological_Process_2021	SRP-dependent cotranslational protein targeting to membrane (GO:0006614)	37/90	4.2435955600739 4E-12	9.91940462167283E-09	5.086087359354 27	133.182301017855	RPL5;RPL30;RPL3;RPL32;RPL10;RPL34;RPL12;RPL36A;RPL10A;RPL8;RPS4X;RPL7A;SEC61A1;RPS16;RPS15A;RPS19;SRP72;RPS18;RPL36;RPL14;RPS3;RPLP2;SEC61B;RPL15;RPS27A;RPL17;RPS13;RPS12;SEC63;RPL19;RPL21;RPS6;RPL35A;RPSA;RPS25;RPL27;RPL29
MSigDB_Hallmark_2020	Oxidative Phosphorylation	57/200	4.1390814848022 8E-10	2.06954074240114E-08	2.913319580126 13	62.9433679383596	COX7B;ACADVL;ACAA2;COX4I1;ETFA;PHB2;TOMM22;NNT;UQCRFS1;ACADM;DLAT;IDH3A;BCKDHA;CPT1A;ECH1;SDHC;SDHD;ATP1B1;COX6B1;POR;NDUFS8;UQCRC1;SUCLG1;VDAC1;SLC25A5;SLC25A4;NDUFB8;ECHS1;RETSAT;COX15;NDUFB5;TIMM13;ETFDH;DLST;UQCR11;ACAT1;PRDX3;LDHA;CYB5R3;POLR2F;SLC25A20;NDUFV2;NDUFV1;ATP6V1C1;NDUFA9;NDUFA5;MDH2;NDUFA4;IDH3G;GOT2;NDUFA1;IMMT;COX6C;CS;GLUD1;ALDH6A1;UQCRQ
KEGG_2019_Mouse	Huntington disease	57/192	7.2418830638037 E-11	2.17256491914111E-08	3.087378188091 57	72.0858186853772	NDUFA13;COX7B;NDUFA11;COX4I1;ATP5C1;ATP5G3;AP2A2;ATP5G1;DNALI1;CREB3L1;UQCRFS1;AP2M1;GPX1;SDHC;SDHD;COX6B1;CREB3;NDUFS8;PLCB4;UQCRC1;VDAC1;SLC25A5;SLC25A4;PLCB2;CREB5;DNAH3;NDUFB8;DNAH1;DCTN2;NDUFB5;DNAH5;ATP5A1;DNAH6;ITPR1;ATP5J;UQCR11;ATP5H;ATP5O;ATP5B;ATP5E;POLR2D;AP2S1;POLR2F;NDUFV3;NDUFV2;POLR2I;NDUFV1;NDUFA9;CREBBP;DNAH11;DNAH10;NDUFA5;NDUFA4;NDUFA1;COX6C;UQCRQ;DNAL1
GO_Biological_Process_2021	protein targeting to ER (GO:0045047)	39/103	2.3572307570716 8E-11	3.67335126310337E-08	4.440493544356 52	108.663088842526	RPL5;RPL30;RPL3;RPL32;RPL10;RPL34;RPL12;RPL36A;RPL10A;RPL8;RPS4X;RPL7A;SEC61A1;RPS16;RPS15A;RPS19;SRP72;RPS18;RPL36;RPL14;RPS3;RPLP2;RPL15;RPS27A;RPL17;RPS13;RPS12;SEC63;RPL19;RPL21;RPS6;RPL35A;RPSA;RPS25;SPCS2;SPCS1;CHMP4B;RPL27;RPL29
MSigDB_Hallmark_2020	Adipogenesis	53/200	2.6234451408989 6E-08	6.55861285224739E-07	2.630145145615 85	45.9123195720788	COX7B;SLC27A1;ACAA2;SCP2;ALDH2;ME1;ACADM;DLAT;ACADS;IDH3A;BCKDHA;ECH1;ELOVL6;ATP1B3;SDHC;POR;TST;COL4A1;ACOX1;GPD2;CAT;UQCRC1;CMPK1;SUCLG1;CD302;ECHS1;CD151;RETSAT;AK2;UQCR11;SLC1A5;LTC4S;ADIPOR2;TOB1;C3;PRDX3;SAMM50;DHRS7B;SLC19A1;ABCA1;ESRRA;NDUFA5;MDH2;IDH3G;IMMT;CS;QDPR;DHRS7;PFKL;REEP5;BCL6;UQCRQ;LPCAT3
GO_Biological_Process_2021	cytoplasmic translation (GO:0002181)	34/93	1.3078226185122 5E-09	1.5285176853862E-06	4.191738168702 54	85.7415943134057	EIF4A1;RPL5;RPL30;RPL3;RPL32;RPL10;RPL34;RPL12;RPL36A;RPL10A;RPL8;RPS4X;RPL7A;RPS16;RPS15A;RPS19;RPS18;RPL36;RPL14;RPS3;RPL15;RPS27A;RPL17;RPS13;RPS12;RPL19;RPL21;RPS6;RPL35A;RPSA;RPS25;EIF3I;RPL27;RPL29
GO_Biological_Process_2021	nuclear-transcribed mRNA catabolic process, nonsense-mediated decay (GO:0000184)	38/113	2.2564527139732 2E-09	2.10978328756496E-06	3.688204274215 93	73.4301991832372	RPL5;RPL30;SMG1;RPL3;RPL32;RPL10;RPL34;RPL12;EIF4A3;RPL36A;RPL10A;RPL8;RPS4X;RPL7A;RPS16;RPS15A;RPS19;PPP2R1A;RPS18;MAGOH;RPL36;RPL14;RPS3;RPLP2;RPL15;RPS27A;RPL17;RPS13;RPS12;RPL19;RPL21;RPS6;PYM1;RPL35A;RPSA;RPS25;RPL27;RPL29
KEGG_2019_Mouse	Alzheimer disease	46/175	2.7519439735386 5E-07	4.12791596030797E-05	2.596368181435 5	39.2201875512072	APP;NDUFA13;COX7B;NDUFB8;NDUFA11;NDUFB5;COX4I1;ATP5A1;ITPR1;ATP5C1;ATP5J;UQCR11;CALML4;ATP5H;ATP5G3;ATP5O;ATP5G1;APH1A;ATP5B;PPP3CA;NCSTN;PPP3R1;APH1C;ATP5E;UQCRFS1;NDUFV3;NDUFV2;NDUFV1;NDUFA9;NDUFA5;BAD;NDUFA4;NDUFA1;SDHC;SDHD;COX6C;COX6B1;NDUFS8;PLCB4;UQCRQ;UQCRC1;CALM3;CALM1;CALM2;PLCB2;GAPDH
GO_Biological_Process_2021	endoplasmic reticulum to Golgi vesicle-mediated transport (GO:0006888)	48/185	2.3375232593349 3E-07	0.00018213202062318	2.552010350804 38	38.9666555355965	DCTN6;PGAP1;TMED10;ATL3;DCTN2;SAR1B;PDCD6;PROS1;GOSR2;RAB1B;DCTN3;TEX261;IER3IP1;USE1;LMAN1;ACTR1A;CAPZB;TMED3;BCAP29;TMED2;TMED1;TMED7;YKT6;CTSC;TMED5;CD55;TMED4;TRAPPC3;SEC13;TMED9;F10;TRAPPC5;DYNLL2;COPZ1;CNIH1;VAMP7;TBC1D20;GOLGB1;GORASP1;COL7A1;CAPZA2;COPG1;ERGIC3;GAS6;ACTR10;CNIH4;COPE;BET1
KEGG_2019_Mouse	Parkinson disease	38/144	2.6152463927148 2E-06	0.000227341459189512	2.604951848302 52	33.4844476412916	NDUFA13;COX7B;NDUFB8;NDUFA11;NDUFB5;LRRK2;COX4I1;ATP5A1;ATP5C1;ATP5J;UQCR11;ATP5H;ATP5G3;ATP5O;ATP5G1;UBE2J1;GNAI2;ATP5B;ATP5E;UQCRFS1;NDUFV3;PRKACA;NDUFV2;NDUFV1;NDUFA9;NDUFA5;NDUFA4;NDUFA1;SDHC;SDHD;COX6C;COX6B1;NDUFS8;UQCRQ;UQCRC1;VDAC1;SLC25A5;SLC25A4
KEGG_2019_Mouse	Oxidative phosphorylation	36/134	3.0312194558601 6E-06	0.000227341459189512	2.668307922170 53	33.9049761777764	NDUFA13;COX7B;NDUFB8;NDUFA11;COX15;NDUFB5;COX4I1;ATP5A1;ATP5K;ATP5C1;ATP5J;UQCR11;ATP5H;ATP5G3;ATP5O;ATP5G1;ATP5B;ATP5E;UQCRFS1;NDUFV3;NDUFV2;NDUFV1;ATP6V1C1;LHPP;NDUFA9;NDUFA5;NDUFA4;NDUFA1;SDHC;SDHD;COX6C;COX6B1;NDUFS8;UQCRQ;UQCRC1;ATP6V0D2
KEGG_2019_Mouse	Focal adhesion	47/199	5.3486576473304 4E-06	0.000320919458839826	2.249367673257 53	27.3043205025979	ITGB1;ROCK1;SHC1;ITGB4;ITGA2B;PIK3R3;ILK;ACTB;MYL12A;EGFR;THBS3;PPP1CB;RAP1B;CDC42;RAP1A;CCND1;AKT2;CHAD;RAC2;AKT1;RAC3;FLNA;FLNB;PIP5K1C;RAC1;VASP;VAV3;PPP1R12A;VWF;LAMB3;PRKCB;CAV2;BAD;LAMB2;CAV1;FN1;PRKCA;BRAF;PARVA;IGF1;RHOA;PPP1CA;PARVG;COL4A1;ITGA10;RAF1;MET
MSigDB_Hallmark_2020	Fatty Acid Metabolism	38/158	2.6584855850025 E-05	0.00044308093083375	2.299195115181 79	24.2224087190292	GCDH;ACADVL;ACAA2;ECHS1;RETSAT;MAOA;ECI2;ETFDH;DLST;HSD17B11;LTC4S;ADIPOR2;LDHA;UROD;ME1;ACADM;ACSS1;ACADS;S100A10;CBR1;CPT1A;HSDL2;MDH2;IDH3G;ECH1;FMO1;SDHC;SDHD;ALDH3A2;ACOX1;GPD2;FASN;EHHADH;APEX1;GPD1;ALDH1A1;SUCLG1;SERINC1
MSigDB_Hallmark_2020	PI3K/AKT/mTOR Signaling	28/105	4.2258629305675 E-05	0.000528232866320937	2.635760590984 47	26.5465951659097	ARF1;RALB;PRKAA2;YWHAB;PRKAG1;SLA;PIK3R3;NOD1;IL2RG;EGFR;PPP2R1B;ARHGDIA;PRKAR2A;CFL1;AKT1;RAC1;AP2M1;ACTR3;VAV3;PLA2G12A;PRKCB;STAT2;PPP1CA;PIKFYVE;PIN1;RAF1;SQSTM1;MYD88

Supplemental Table S1: Gene-set Enrichment Analyses (GSEA) for FR/FF diet mice BAL bulk RNAseq (pre-injury). OX-PHOS genes enriched are listed for the MSigDB and KEGG databases (above two boxes). All gene sets enriched ($p<0.05$) listed in table below with the two OX-PHOS entries highlighted in yellow.

Figure S3A1

(FF versus ND (t=0))

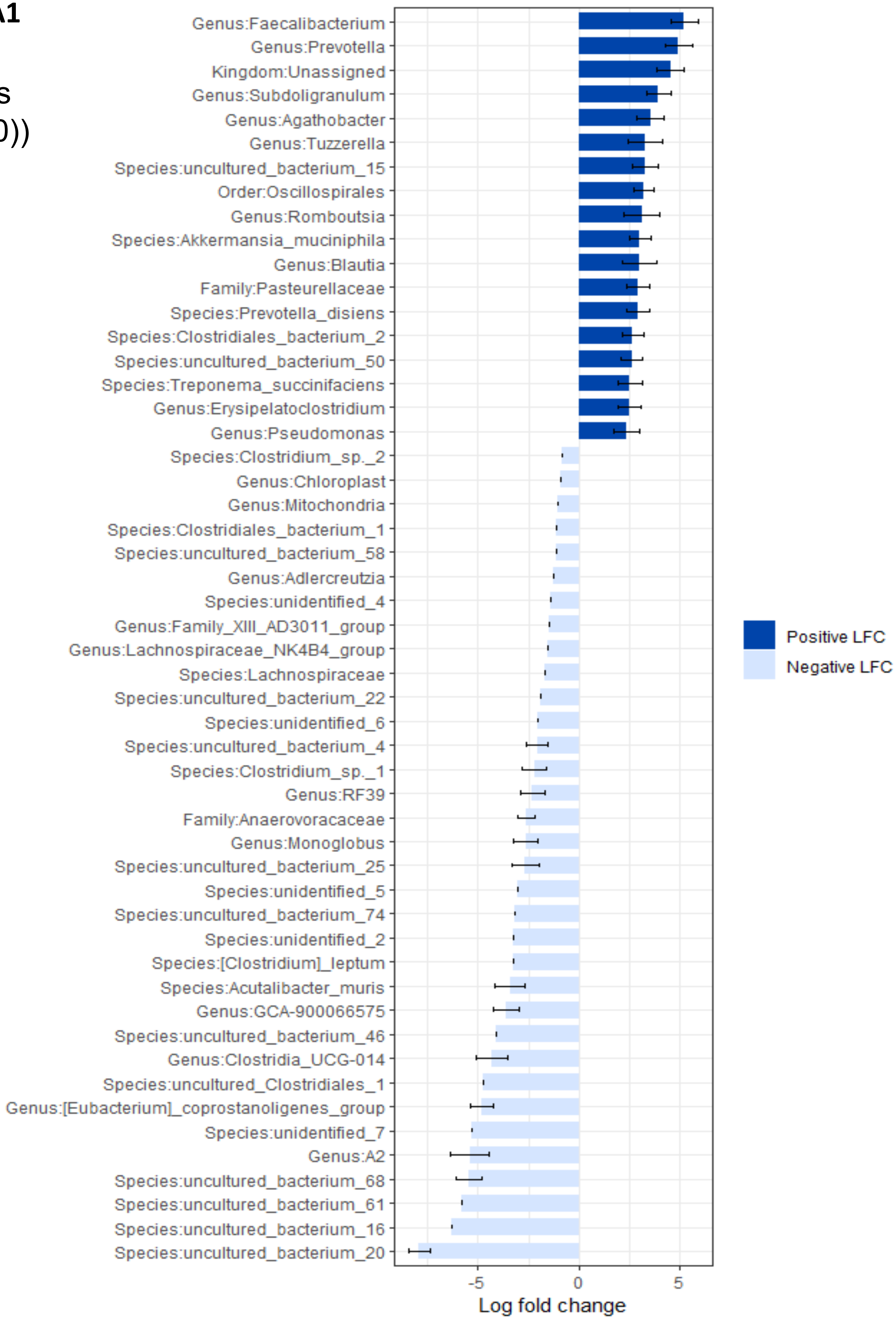

Figure S3: Complete comparison of taxa enriched in FF group vs baseline t=0 (ND) group.

Figure S3A2

(FR 1wk versus ND (t=0))

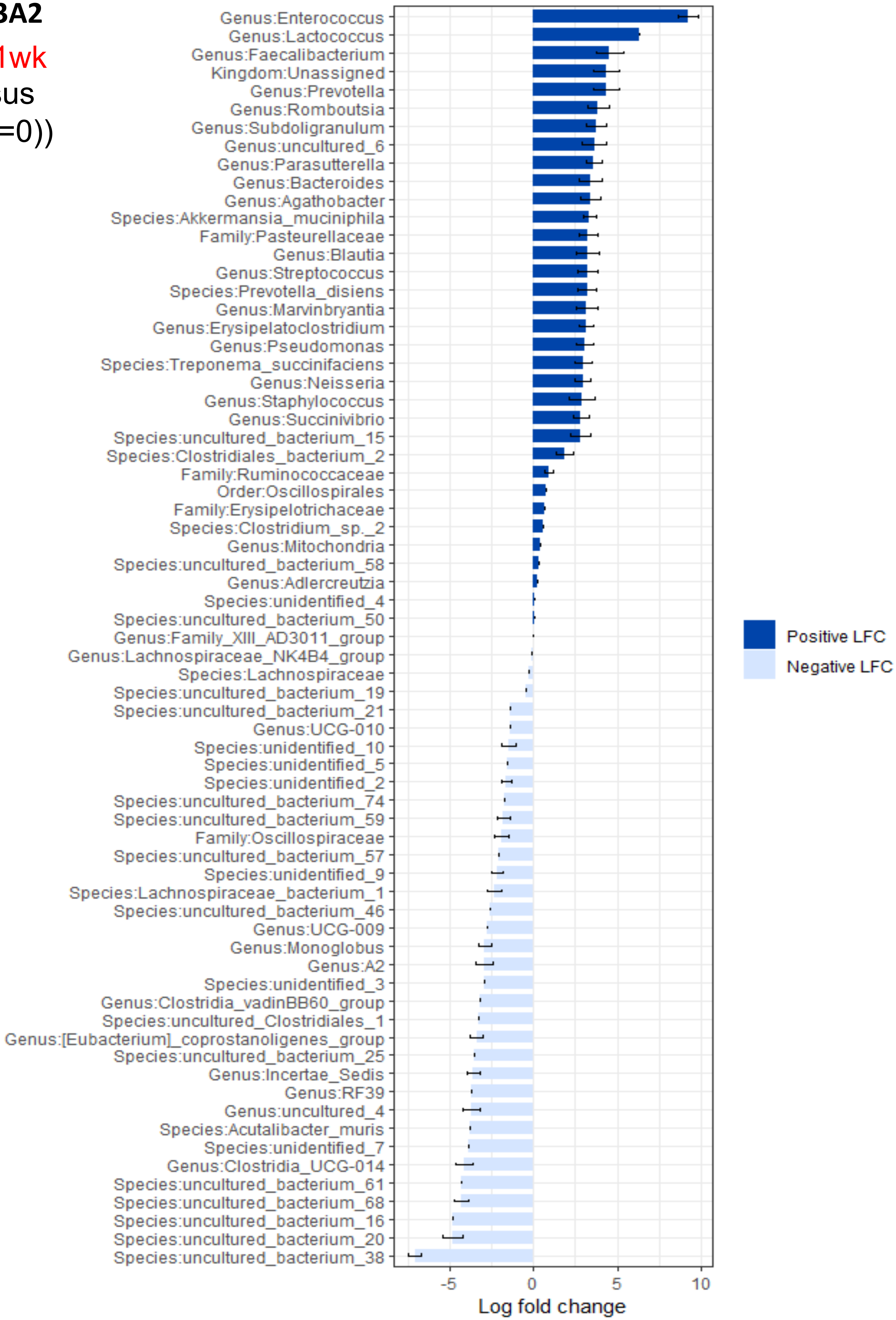

Figure S3: Complete comparison of taxa enriched in FR 1wk group vs baseline t=0 (ND) group.

Figure S3A3

(FR 2wk versus ND (t=0))

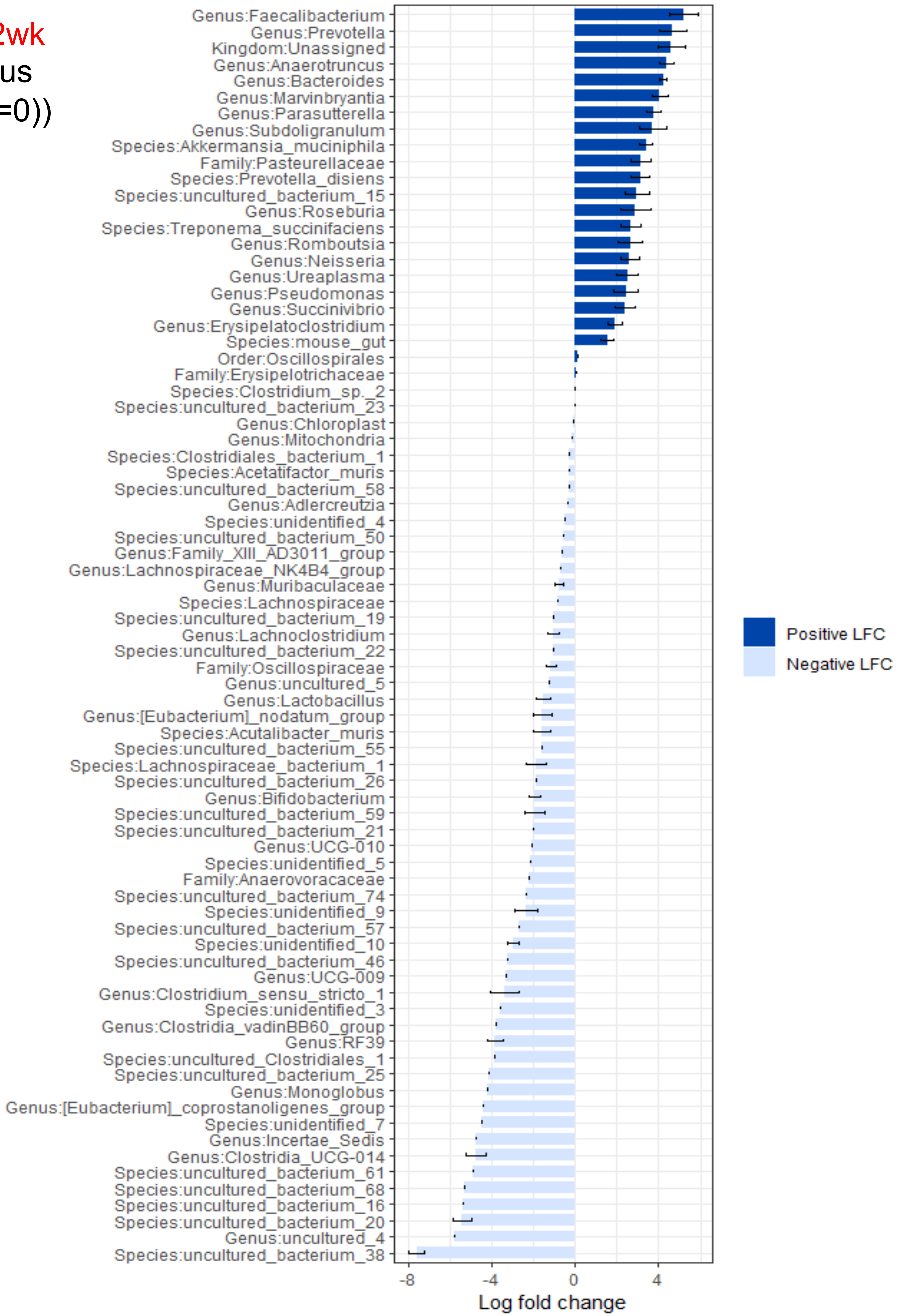

Figure S3: Complete comparison of taxa enriched in FR 2wks group vs baseline t=0 (ND) group.

Figure S3B

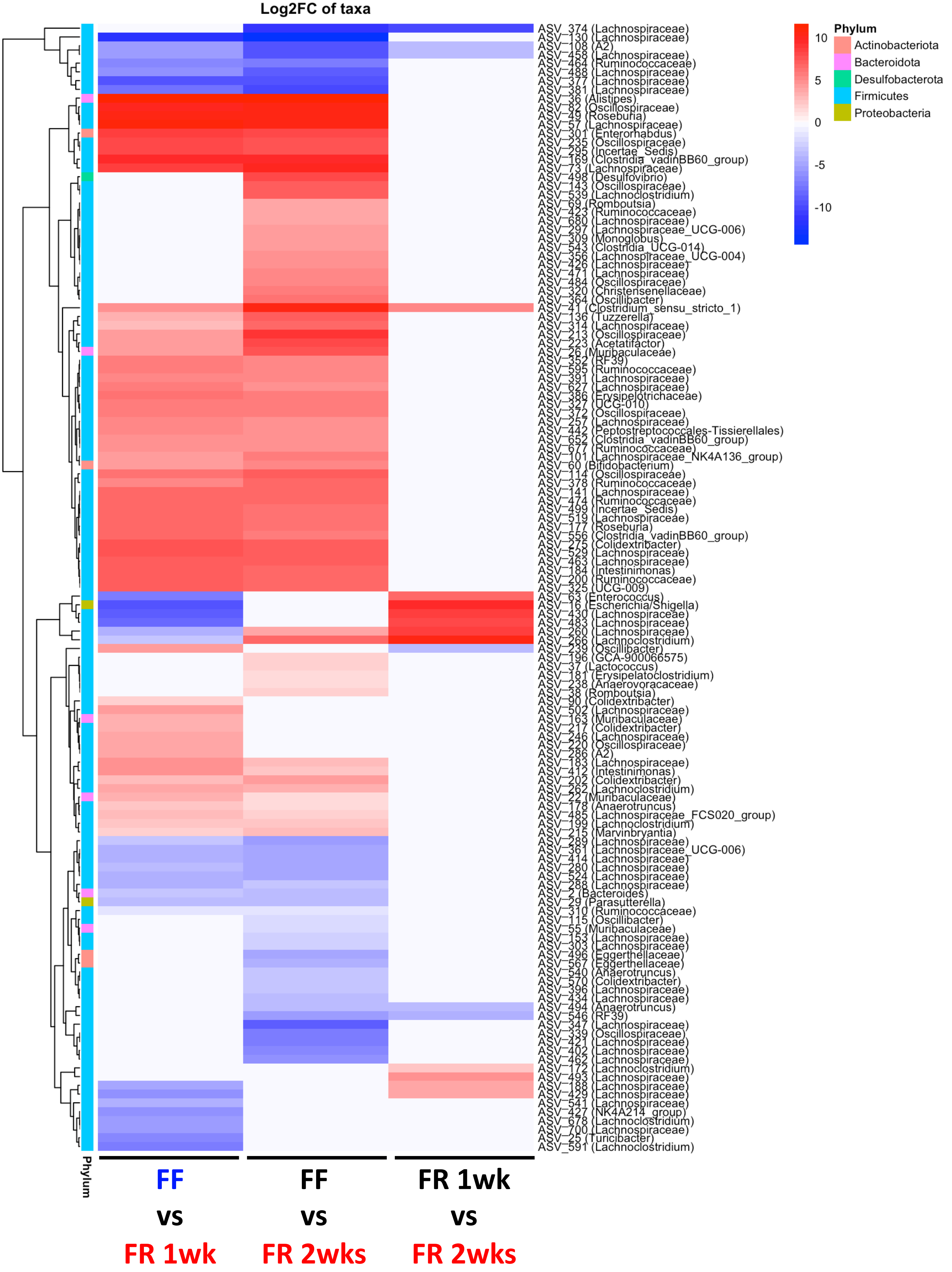

Figure S3: Comparison of taxa enriched in each group vs the other 2 groups.

Figure S3C1

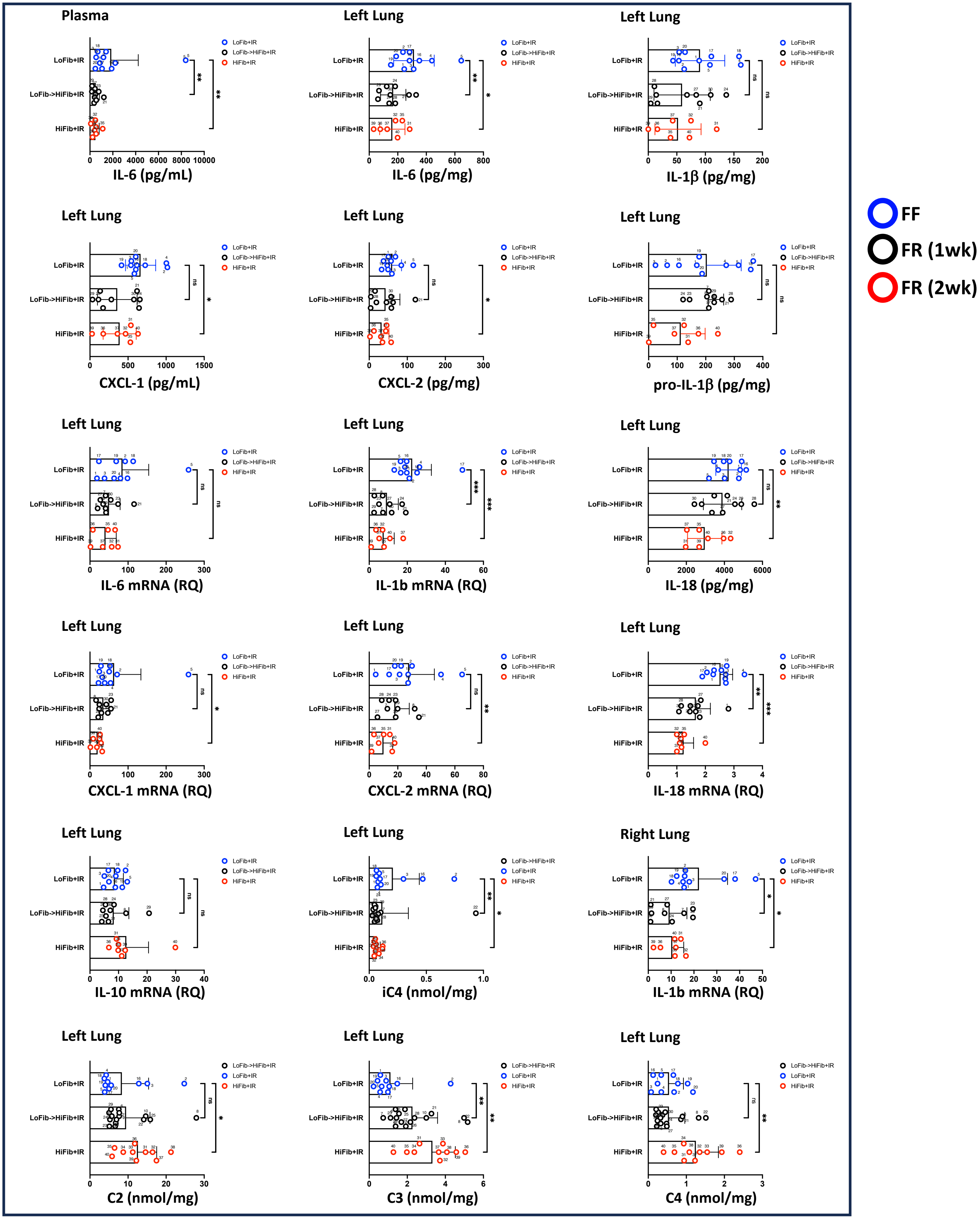

Figure S3C: Summary of all immune tone and lung injury measurements for all 3 groups. Comprehensive data from all measurements conducted including all three groups: FF, FR (1 week) and FR (2 weeks) with individual mouse #s annotated. Plasma and lung lung injury measurements and left and right lung inflammation marker measurements (A) in mice after lung IR injury. SCFA measurements in stool and portal blood (B) and plasma and Right Lung (C). n=8-15 mice in each group depending on the specific transcriptomic/proteomic/metabolite measurement.

Figure S3C2

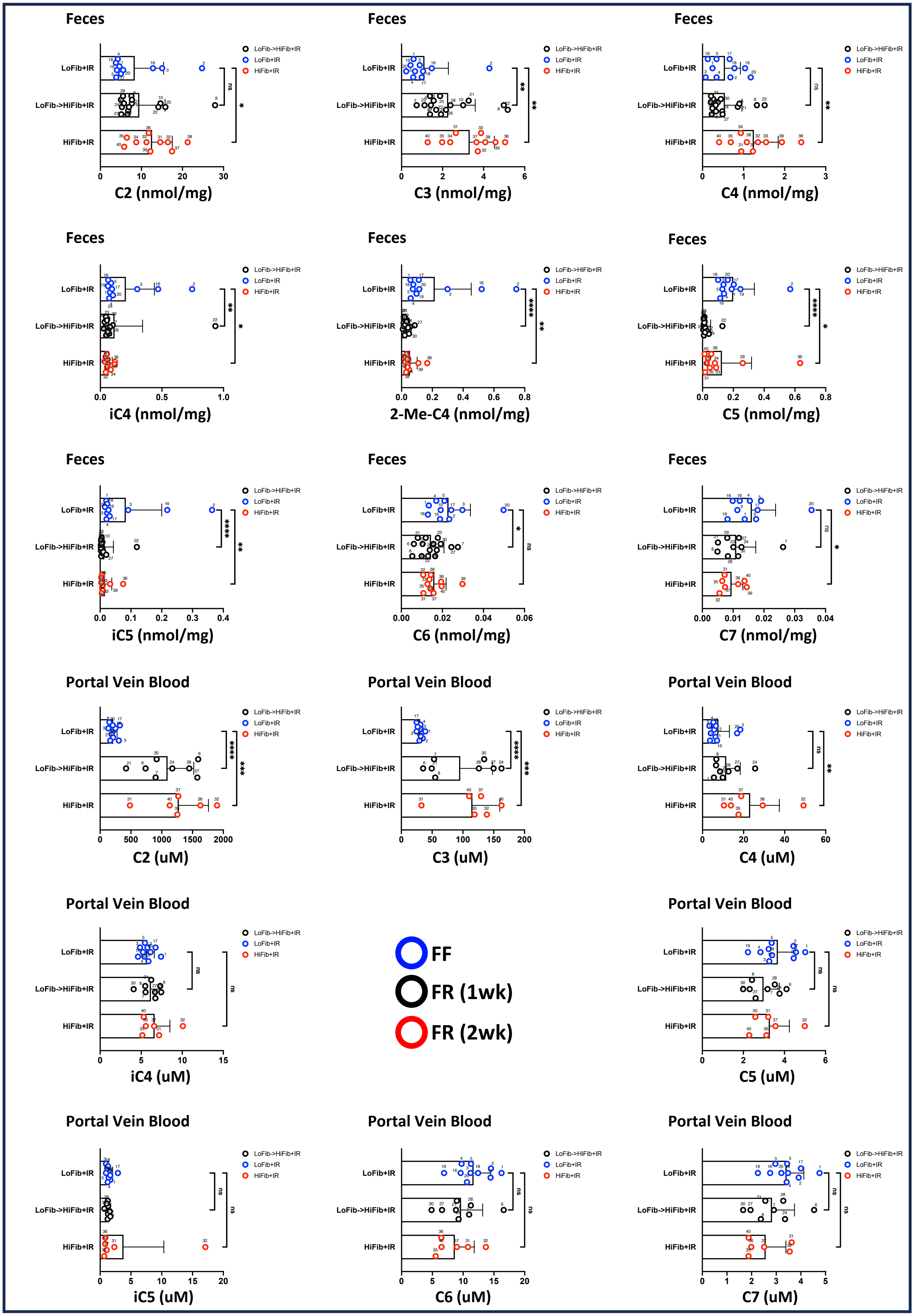

Figure S3: Summary of all immune tone and lung injury measurements for all 3 groups. Comprehensive data from all measurements conducted including all three groups: FF, FR (1 week) and FR (2 weeks) with individual mouse #s annotated. Plasma and lung lung injury measurements and left and right lung inflammation marker measurements (A) in mice after lung IR injury. SCFA measurements in stool and portal blood (B) and plasma and Right Lung (C). n=8-15 mice in each group depending on the specific transcriptomic/proteomic/metabolite measurement.

Figure S3C3

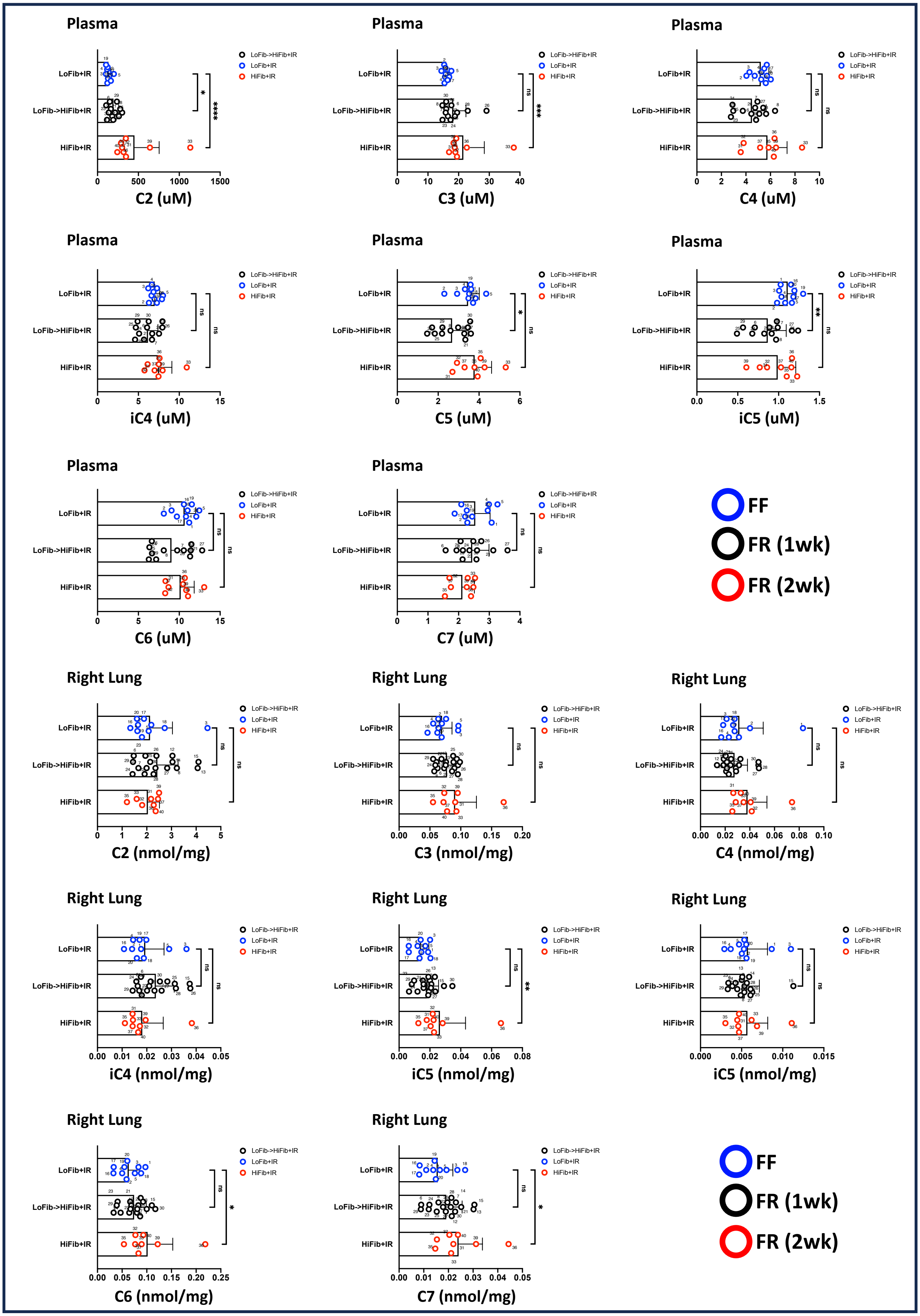

Figure S3: Summary of all immune tone and lung injury measurements for all 3 groups. Comprehensive data from all measurements conducted including all three groups: FF, FR (1 week) and FR (2 weeks) with individual mouse #s annotated. Plasma and lung lung injury measurements and left and right lung inflammation marker measurements (A) in mice after lung IR injury. SCFA measurements in stool and portal blood (B) and plasma and Right Lung (C). n=8-15 mice in each group depending on the specific transcriptomic/proteomic/metabolite measurement.

Supplemental Table S2

Correlation Table (SILVA)

Symbol	Actinobacteriota	Bacteria_Total	Bacteroidota	Firmicutes	F/B SILVA	OTU_Count	Proteobacteria	Verrucomicrobiota	Caproate (F)	Heptanoate (F)	Propionate (F)	CXCL1 ELISA (LL)	IL-1b ELISA (LL)	IL-1b qPCR (LL)	IL-1B qPCR (RL)	IL-18 qPCR (LL)
“Actinobacteriota*”	0.986***	−0.379*	−0.694***	0.57***	0.805***		−0.376*					0.587**	0.396*	0.49*		0.475*
“Bacteroidota*”	−0.728***		0.901***	−0.928***	−0.866***		0.598***	−0.415*	−0.429*	−0.468**	0.394*	−0.621***	−0.442*	−0.646***	−0.536**	−0.753***
“Firmicutes*”	0.729***		−0.872***	0.942***	0.95***		−0.485**		0.375*	0.451**	−0.358*	0.612**	0.441*	0.518**	0.462*	0.716***
“Proteobacteria*”	−0.405*		0.636***	−0.521**	−0.511**		0.969***	−0.523**	−0.412*			−0.609**	−0.458*	−0.7***		−0.452*
“Verrucomicrobiota*”		0.371*				0.479**	−0.521**	0.987***	0.339*					0.471*		
FECES																
Acetate											0.731***					
Propionate				−0.448**		−0.418*				−0.496**	1***		−0.4*		−0.446*	−0.672***
Butyrate											0.741***					−0.427*
Isobutyrate											0.339*					
2-methylbutyrate	0.377*		−0.441*	0.488**	0.42*		−0.375*					0.404*				
Valerate	0.439*		−0.467**	0.384*	0.408*				0.349*							
Isovalerate	0.382*		−0.386*	0.416*	0.386*		−0.345*					0.407*				
Caproate			−0.344*	0.417*	0.356*				1***	0.886***					0.395*	
Heptanoate			−0.34*	0.502**	0.411*				0.886***	1***	−0.496**				0.549**	0.573**
LEFT LUNG																
CXCL1 ELISA	0.562**		−0.571**	0.531**	0.658***		−0.569**					1***	0.777***	0.645***		0.403*
CXCL1 qPCR				0.402*											0.584**	
CXCL2 ELISA							−0.508**					0.75***	0.682***	0.437*		
CXCL2 qPCR			−0.398*	0.477*	0.487*							0.452*	0.419*			0.526**
IL-1b ELISA	0.413*			0.391*	0.481*		−0.418*				−0.4*	0.777***	1***	0.593**		
IL-1b qPCR	0.573**		−0.607**	0.537**	0.497*		−0.648***	0.464*				0.645***	0.593**		0.746***	0.431*
IL-6 ELISA	0.405*		−0.461*	0.558**	0.445*		−0.414*				−0.476*	0.564**	0.594**	0.437*	0.657***	0.469*
IL-6 qPCR				0.471*								0.416*	0.428*		0.491*	
IL-10 qPCR																
IL-18 ELISA																
IL-18 qPCR	0.488*		−0.584**	0.765***	0.68***	0.457*				0.573**	−0.672***	0.403*		0.431*	0.398*	1***
pro-IL-1b ELISA													0.41*	0.402*		

Supplemental Table S2: Correlation table (all Groups) and including 16S data. Summary of individual correlation coefficients between bacteria phyla, stool SCFAs, immune tone and lung injury markers. n=26-27 mice.

Figure S4

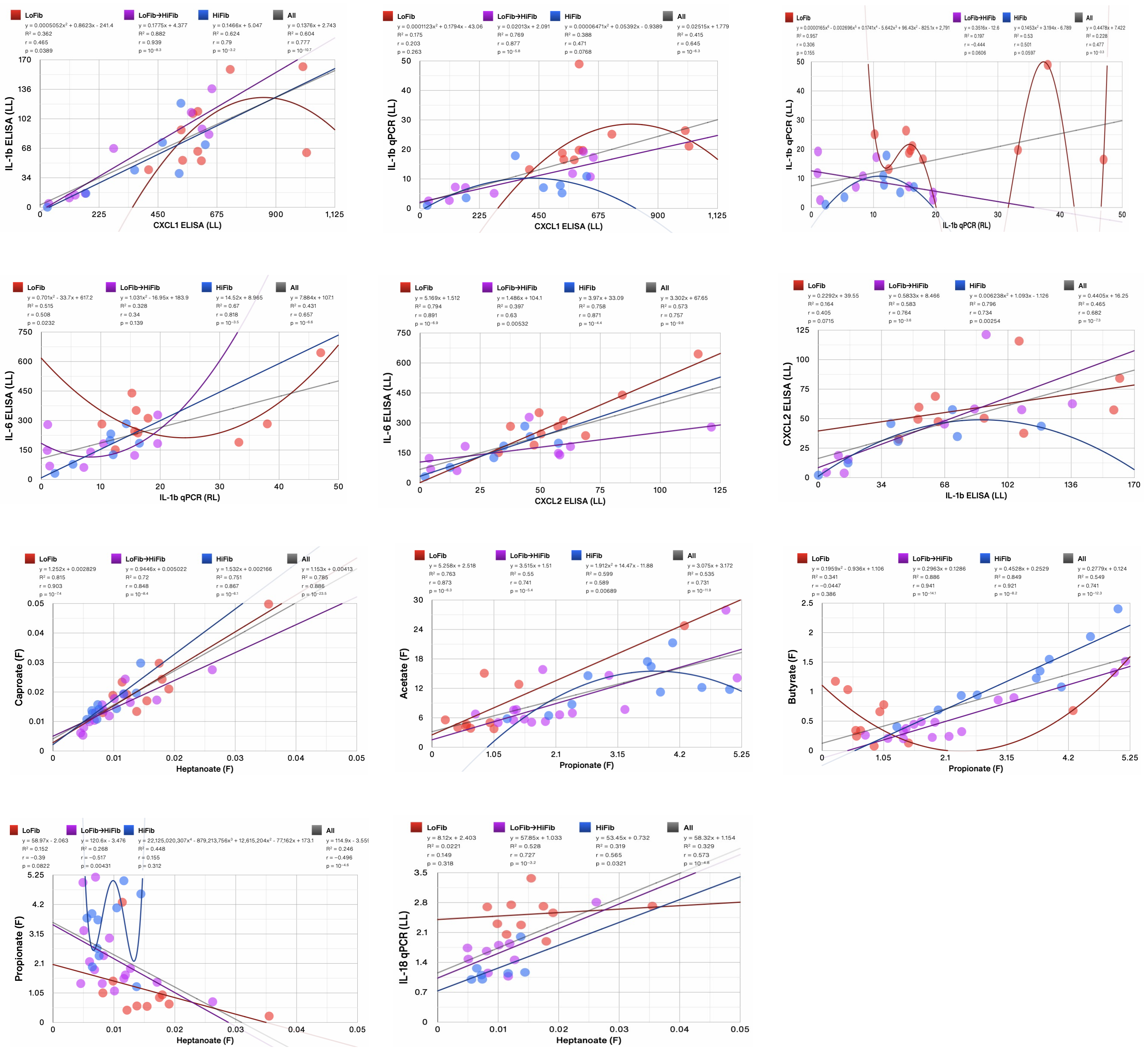

Figure S4: Correlations between immune tone and lung injury markers, SCFAs, and bacterial taxa (all Groups). Significant positive correlations between cytokines/chemokines, between left and right lung IL-1b levels (row 1 and 2), and between metabolites (row 3) and negative correlation between heptanoate and propionate (row 4). n=26-27 mice as shown on each graph.

Supplemental Table S3

Relation	Strain	Genotype	Phenotype/Role
**Parent strain**	VPI-5482	Wild-type, intact genome	Standard reference strain; fully functional; sensitive to 2-FdU. NCBI Reference Genome: NC_004663
**Genetic mutant parent strain**	DeltaTDK	*tdk* gene (BT_4729 Thymidine Kinase Gene) deleted	Gene ID: BT_4729 in VPI-5482 genome Resistant to 2-FdU; used as a platform for genetic engineering; serves as the "wild-type" in experimental contexts.
**Propionate mutant strain**	Delta1686-89	*tdk* gene deleted, BT_1686-89 deleted	Propionate metabolism deficient; used to study SCFA metabolism and its effects on microbial and host physiology.

Supplemental Table S3: *B.theta* strain information

Figure S5

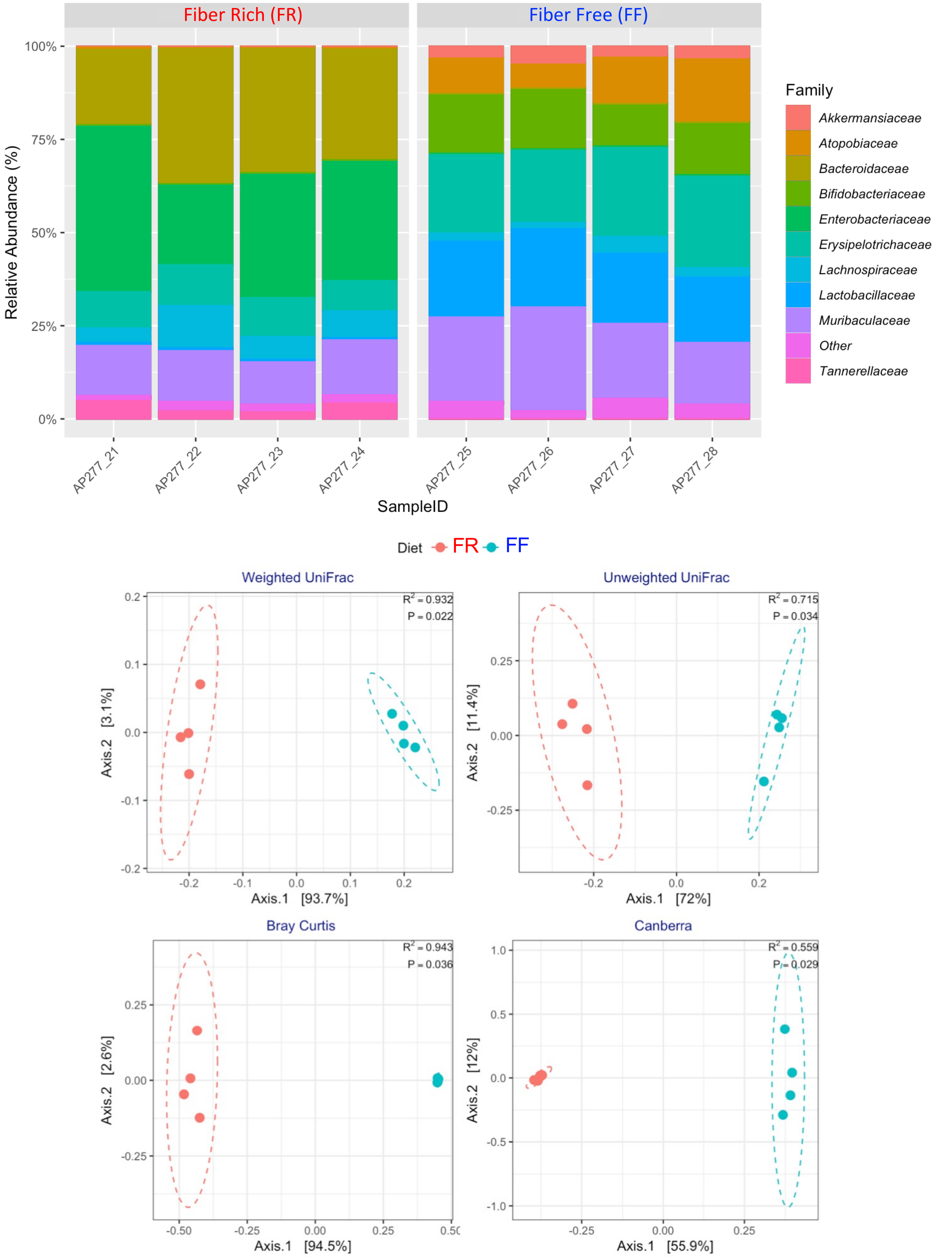

Figure S5: 16S rRNA sequence analysis of FR and FF mice gut microbiota from snATACseq experiment. Family level comparison between n=4 mice gut microbiota in each group.

Figure S6

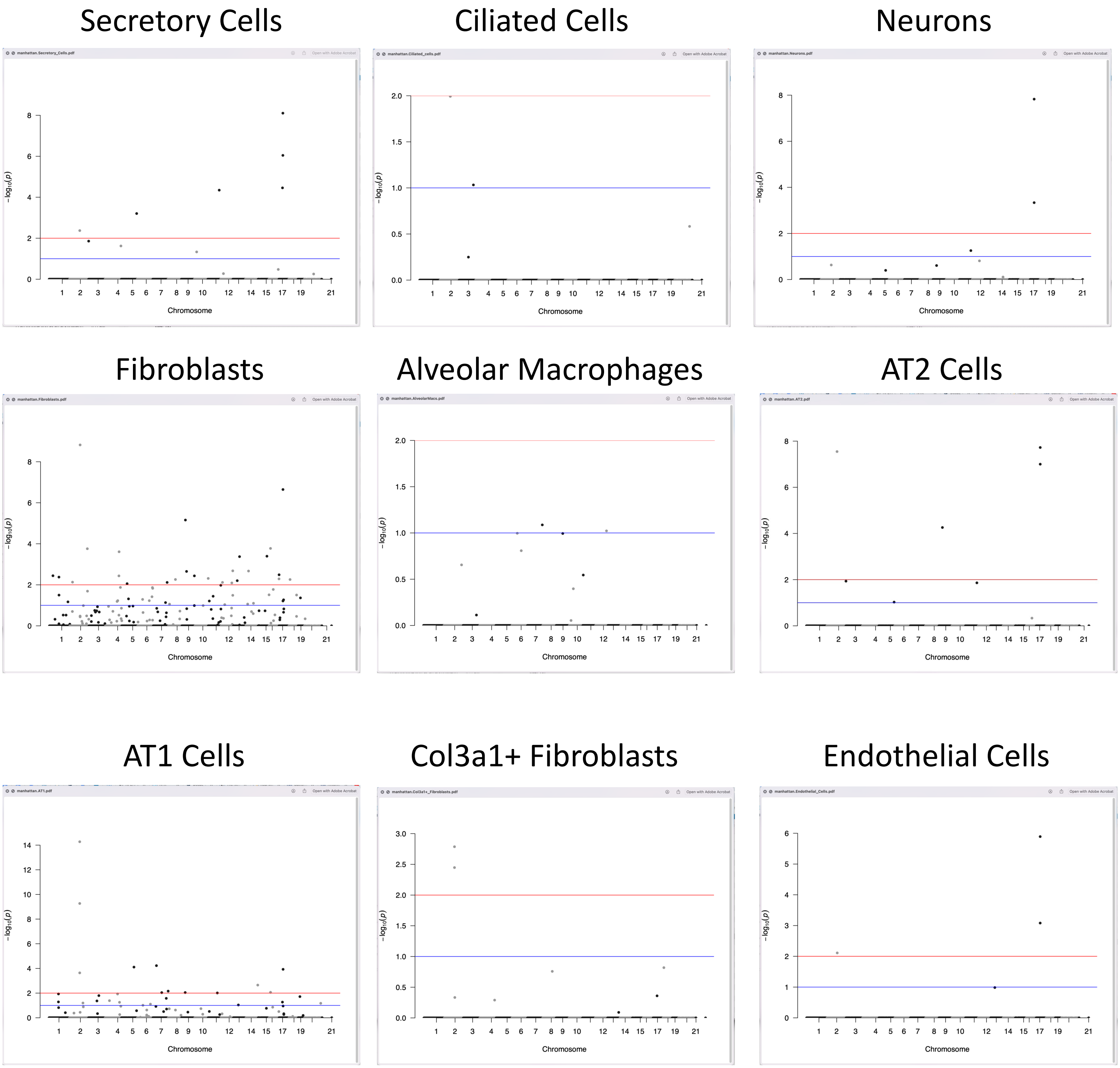

Figure S6: Manhattan Plots for Chromatin Accessibility loci from snATACseq.

Supplemental Table S4

Table S4A

BaselineProp.clusters	BaselineProp.Freq	PropMean.HighFiber	PropMean.LowFiber	PropRatio	Tstatistic	P.Value	FDR
Macrophages	0.0641549402330156	0.0799086280096742	0.0151448576652441	5.27628781834355	5.07910491440752	2.25293885393785e-06	3.15411439551299e-05
CD4_T_cell	0.052050234528673	0.059440250737777	0.0185911074675084	3.19724098425338	3.43411094617065	0.000925663644030091	0.00647964550821064
B_cell	0.0494779845665002	0.0585447798984303	0.0214024589587154	2.73542306570293	3.17298942313917	0.00210818430297654	0.00791508806846228
Col3a1+_Fibroblasts	0.0680889695869269	0.0421565480396635	0.109397686892566	0.38535136561949	-3.15008691582251	0.00226145373384637	0.00791508806846228
Alveolar Macs	0.127099409895597	0.100771353106679	0.170008543833376	0.592742875354807	-1.8168421948071	0.0728078201794595	0.203861896502486
Mast_cells	0.0220910879104252	0.0259456327949061	0.0070893501465127	3.65980410879676	1.96859318259878	0.0961911342133788	0.224445979831217
Fibroblasts	0.115297321833863	0.0931846963794225	0.148313211538281	0.628296666311285	-1.37751201140774	0.172013279238302	0.344026558476604
AT1	0.0969889544560448	0.0876001099811132	0.113472416566582	0.771994751074262	-0.715401940669935	0.476344551623919	0.753011270496573
Mesothelial_Cells	0.0140717203812982	0.0119536196483763	0.0149230486592283	0.801017266735526	-0.697239063789537	0.487578477408955	0.753011270496573
Neurons	0.0832198517173551	0.105061403468425	0.0806240255146501	1.30310292493811	0.595114674872549	0.553366677731809	0.753011270496573
Endothelial_Cells	0.10425177787865	0.121397443925048	0.0991189326849993	1.224765447292	0.538506358827145	0.591651712533022	0.753011270496573
Ciliated_cells	0.0484188228173703	0.0494491605878084	0.0498935863467694	0.99109252728653	0.401130232533909	0.690789910656814	0.781324434701315
AT2	0.0868512634286579	0.0950587383572102	0.0829293404880106	1.1462618392697	0.352273379254484	0.725515546508364	0.781324434701315
Secretory_Cells	0.0679376607656226	0.0695276350654654	0.0690914332375567	1.00631339961365	-0.0285153688864745	0.97731874977729	0.97731874977729

Table S4B

cellType	Significant Gene Count
Endothelial_Cells	2
Col3a1+_Fibroblasts	3
CD4_T_cell	1
AT1	13
AT2	4
Alveolar_Macs	1
B_cell	0
AlveolarMacs	8
Fibroblasts	34
Neurons	2
Macrophages	0
Ciliated_cells	2
Mast_cells	0
Secretory_Cells	1
Mesothelial_Cells	1

Table S4C

cellType	Significant Gene Count
Endothelial_Cells	3
Col3a1+_Fibroblasts	2
CD4_T_cell	0
AT1	27
AT2	7
Alveolar_Macs	0
B_cell	0
AlveolarMacs	3
Fibroblasts	53
Neurons	3
Macrophages	0
Ciliated_cells	2
Mast_cells	0
Secretory_Cells	9
Mesothelial_Cells	0

Table S4: Cell Populations from digested lungs of FR and FF mice (pre-injury) from snATACseq analysis. (A) Cell types that were significantly different are highlighted in yellow. (B) Differentially expressed genes with adjusted p values <0.1 for each cell type identified. (C) Number of Differentially sequenced ATAC chromatin segments with adjusted p values <0.1

Supplemental Table S4D

Ciliated Cells	p_val	avg_log2FC	pct.1	pct.2	p_val_adj
Gm10800	8.45E-08	0.56792722	0.973	0.917	0.00184337
Sfi1	3.15E-06	0.53869866	0.968	0.917	0.06864342
Smim20	4.61E-06	-1.037833	0.293	0.621	0.10066109

Endothelial Cells	p_val	avg_log2FC	pct.1	pct.2	p_val_adj
Gm10800	4.14E-08	0.37904698	0.986	0.974	0.00090343
Prl8a9	1.76E-06	-2.2512669	0.034	0.136	0.0383567

Endothelial Cells	p_val	avg_log2FC	pct.1	pct.2	p_val_adj
Gm10800	4.24E-10	0.6181366	0.955	0.89	9.25E-06

Macrophages	p_val	avg_log2FC	pct.1	pct.2	p_val_adj
Slc5a8	6.36E-05	3.11274674	0.284	0.05	1

Neurons	p_val	avg_log2FC	pct.1	pct.2	p_val_adj
Gm10800	3.58E-07	0.44388716	0.983	0.98	0.00780578
Ftsj3	3.81E-06	-1.044998	0.224	0.436	0.08324653

CD4 T Cells	p_val	avg_log2FC	pct.1	pct.2	p_val_adj
Mterf4	1.51E-06	-1.8794499	0.113	0.481	0.03294199

B Cells	p_val	avg_log2FC	pct.1	pct.2	p_val_adj
Gm16506	6.92E-05	-2.2721327	0.049	0.226	1

Ciliated Cells	p_val	avg_log2FC	pct.1	pct.2	p_val_adj
Gm10800	8.45E-08	0.56792722	0.973	0.917	0.00184337
Sfi1	3.15E-06	0.53869866	0.968	0.917	0.06864342
Smim20	4.61E-06	-1.037833	0.293	0.621	0.10066109

Mast Cells	p_val	avg_log2FC	pct.1	pct.2	p_val_adj
Gm773	4.01E-05	-3.4859614	0.054	0.438	0.87582308

Mesothelial Cells	p_val	avg_log2FC	pct.1	pct.2	p_val_adj
Oasl1	2.73E-06	-3.7321952	0.038	0.415	0.05961844

Supplemental Table S4D: Differentially expressed genes with adjusted p values <0.1 or top hit for each cell type identified

Supplemental Table S4D (cont'd)

AT1	p_val	avg_log2FC	pct.1	pct.2	p_val_adj
Gm10800	3.37E-22	1.09882049	0.891	0.747	7.35E-18
Gm10801	3.78E-09	0.53812844	0.894	0.795	8.25E-05
Fah	9.66E-08	-0.7829363	0.413	0.634	0.00210871
Drg1	2.50E-07	0.51154178	0.894	0.872	0.00545063
Sfi1	2.93E-07	0.5009006	0.886	0.839	0.00639399
Gm21738	4.13E-07	1.00793447	0.481	0.388	0.00900328
Hrct1	6.61E-07	-1.016074	0.188	0.385	0.01442993
Rsph1	1.41E-06	-1.1631479	0.201	0.377	0.03085256
Tor3a	1.88E-06	-0.5698206	0.522	0.729	0.04106224
Gramd2	2.43E-06	-0.4901285	0.69	0.828	0.05292233
Gm10840	2.56E-06	-0.9740725	0.201	0.392	0.05580245
Pip4k2a	2.56E-06	0.79724203	0.606	0.549	0.05591921
Rrp36	3.98E-06	-1.1500449	0.101	0.271	0.08684494

AT2	p_val	avg_log2FC	pct.1	pct.2	p_val_adj
Gm10800	1.58E-15	0.6378608	0.977	0.977	3.44E-11
Drg1	5.15E-08	0.45680282	0.963	0.904	0.00112404
Sfi1	1.19E-06	0.41123116	0.938	0.922	0.0258644
Gm21738	2.45E-06	0.60591827	0.761	0.74	0.05340354

AlveolarMacs	p_val	avg_log2FC	pct.1	pct.2	p_val_adj
Skint6	8.15E-08	0.92074443	0.737	0.593	0.00177925
Adamtsl1	5.23E-07	0.80258909	0.758	0.657	0.01141027
Myo5b	1.75E-06	1.02808589	0.716	0.61	0.03827214
Il12rb2	1.90E-06	-0.7073646	0.395	0.663	0.04156586
Fam49a	2.42E-06	-0.5545763	0.658	0.837	0.05290664
Rtn4	2.53E-06	-0.4956005	0.689	0.86	0.05528988
Calcoco1	3.67E-06	-0.890162	0.274	0.513	0.08006858
Phlda1	3.98E-06	-0.6907483	0.305	0.573	0.08692424

Col1a1+ Fibroblasts	p_val	avg_log2FC	pct.1	pct.2	p_val_adj
Gm10800	1.22E-08	0.91004407	0.87	0.721	0.00026657
Tspan9	1.58E-07	-0.5363486	0.903	0.962	0.00345675
Lrfn5	1.26E-06	0.97046371	0.676	0.442	0.02758949

Supplemental Table S4D: Differentially expressed genes with adjusted p values <0.1 or top hit for each cell type identified

Supplemental Table S4D (cont'd)

Fibroblasts	p_val	avg_log2FC	pct.1	pct.2	p_val_adj
Gm10800	2.06E-13	0.66117124	0.917	0.764	4.49E-09
Slc22a13	1.85E-09	-1.4430269	0.134	0.31	4.04E-05
Slc29a1	2.42E-09	-0.9072987	0.368	0.586	5.28E-05
Slc7a10	8.06E-09	-0.8362386	0.438	0.63	0.00017597
Mta1	1.11E-08	-0.8220345	0.401	0.597	0.0002429
Serpina3n	1.69E-08	-1.2832704	0.096	0.268	0.00036836
Crip2	4.18E-08	-0.8646236	0.32	0.518	0.00091196
Siah2	8.91E-08	-0.7102475	0.322	0.54	0.00194405
Hif3a	1.45E-07	-0.5839225	0.471	0.688	0.00316101
Pdzrn4	1.64E-07	0.71374901	0.788	0.655	0.00358156
Gas1	3.14E-07	-0.9599364	0.217	0.405	0.00684282
Rnase10	3.59E-07	-0.8020289	0.254	0.458	0.00782372
Pex5l	4.81E-07	0.78047252	0.66	0.54	0.01048769
Tmem241	4.97E-07	-0.5346199	0.657	0.784	0.01083553
Tmigd1	5.44E-07	-0.9546125	0.164	0.334	0.01186338
Nxph1	8.33E-07	0.51962801	0.7	0.603	0.01817245
Zbtb16	8.35E-07	-0.4788074	0.854	0.882	0.01822505
Mroh2a	9.30E-07	0.66963062	0.751	0.652	0.02029348
Ddc	1.06E-06	-0.5300688	0.592	0.751	0.0232049
Htra4	1.12E-06	-0.7712884	0.181	0.367	0.02451405
Chchd5	1.25E-06	-0.7351588	0.207	0.416	0.02717745
Lrp3	1.40E-06	-0.9774273	0.285	0.441	0.03057343
Sfi1	1.43E-06	0.49682509	0.821	0.751	0.03111153
Styx	1.61E-06	-0.8896195	0.224	0.411	0.03523635
Hs3st5	1.76E-06	0.78275767	0.668	0.584	0.03833655
Dync1i1	2.14E-06	0.71669438	0.723	0.597	0.04667499
Gm21738	2.26E-06	0.64317718	0.574	0.455	0.0494238
Nat6	2.46E-06	-0.9733163	0.202	0.356	0.05359857
Vat1l	2.46E-06	0.88020805	0.539	0.427	0.05378393
Tmem52	2.54E-06	-1.5744588	0.076	0.203	0.05546009
Kcnh2	3.10E-06	-0.4894116	0.554	0.737	0.06756673
Il21r	3.38E-06	-0.8366558	0.36	0.534	0.07375502
Grhl2	3.61E-06	0.98604855	0.481	0.373	0.07868866
Cd2	4.52E-06	-0.8615392	0.204	0.378	0.09855076

Supplemental Table S4D: Differentially expressed genes with adjusted p values <0.1 or top hit for each cell type identified

Supplemental Table S4E

Endothelial Cells	p_val	avg_log2FC	pct.1	pct.2	p_val_adj
chr17-39842818-39846951	5.48E-12	0.58190718	0.95	0.897	1.28E-06
chr17-39847439-39848940	3.54E-09	0.62101982	0.765	0.688	0.00082965
chr2-98666906-98667526	3.31E-08	0.29469385	0.974	0.967	0.00775961

Neurons	p_val	avg_log2FC	pct.1	pct.2	p_val_adj
chr17-39842818-39846951	6.34E-14	0.51260263	0.989	0.995	1.49E-08
chr17-39847439-39848940	1.97E-09	0.48588032	0.937	0.891	0.00046234
chr11-109010949-109012578	2.37E-07	0.56263328	0.75	0.678	0.05560453

CD4 T Cells	p_val	avg_log2FC	pct.1	pct.2	p_val_adj
chr3-128255365-128256293	1.39E-06	-12.024677	0	0.115	0.3268215

B Cells	p_val	avg_log2FC	pct.1	pct.2	p_val_adj
chr1-119482661-119483807	9.88E-07	-11.487034	0	0.113	0.23163936

**Ciliated Cells**	**p_val**	**avg_log2FC**	**pct.1**	**pct.2**	**p_val_adj**
chr2-98666906-98667526	4.34E-08	0.54858768	0.952	0.879	0.01015909
chr3-138843002-138844292	3.96E-07	-10.903807	0	0.106	0.09282599

Mesothelial Cells	p_val	avg_log2FC	pct.1	pct.2	p_val_adj
chr5-111674930-111676918	2.79E-06	-12.756341	0	0.293	0.65479161

Macrophages	p_val	avg_log2FC	pct.1	pct.2	p_val_adj
chr8-46496841-46498230	5.62E-06	-2.267465	0.07	0.375	1

Supplemental Table S4E: Number of Differentially sequenced ATAC chromatin segments with adjusted p values <0.1 or top hit only.

Supplemental Table S4E (cont'd)

AT1	p_val	avg_log2FC	pct.1	pct.2	p_val_adj
chr2-98666906-98667526	2.26E-20	0.96543322	0.845	0.681	5.31E-15
chr2-98666067-98666807	2.29E-15	0.53987352	0.976	0.923	5.37E-10
chr7-35112559-35114545	2.54E-10	-2.5062086	0.03	0.187	5.94E-05
chr5-119650936-119652216	3.33E-10	-2.1182959	0.046	0.216	7.81E-05
chr17-39842818-39846951	5.04E-10	0.77406254	0.902	0.74	0.00011804
chr2-98662103-98663137	9.77E-10	0.50079758	0.894	0.788	0.00022904
chr14-118000919-118002398	9.56E-09	-1.8512331	0.054	0.22	0.00224074
chr7-144597913-144598951	2.95E-08	-2.9525561	0.014	0.121	0.00691628
chr16-17795058-17797913	3.67E-08	-1.4551232	0.098	0.278	0.00858965
chr9-35304729-35306073	3.85E-08	0.79712037	0.579	0.447	0.00902947
chr7-84605208-84607160	3.91E-08	-0.7892221	0.315	0.553	0.00915183
chr11-86960701-86963361	4.15E-08	-1.11973	0.16	0.385	0.00972927
chr1-92437443-92439004	4.99E-08	-1.1244352	0.158	0.377	0.01169841
chr4-119462406-119463945	5.05E-08	-1.8688671	0.049	0.201	0.01183479
chr3-101109495-101111093	6.82E-08	-1.9172725	0.043	0.194	0.01599044
chr19-17836678-17838545	8.23E-08	-1.9721057	0.043	0.183	0.0192865
chr7-126921886-126923365	1.14E-07	-1.3815584	0.095	0.278	0.02670116
chr4-43725714-43731824	1.75E-07	-0.7993511	0.299	0.52	0.04106232
chr3-83067422-83069137	1.84E-07	-1.0906466	0.155	0.355	0.04319636
chr1-92030144-92031247	2.21E-07	-1.5804839	0.068	0.223	0.05179198
chr17-33828289-33830049	2.30E-07	-1.8775898	0.043	0.179	0.05386551
chr4-137695090-137696707	2.38E-07	-1.8088032	0.049	0.19	0.05576943
chr2-128698063-128699549	2.71E-07	-1.101312	0.144	0.374	0.06362335
chrX-157597366-157599144	2.87E-07	-1.883634	0.043	0.176	0.06715582
chr6-125330464-125332226	3.34E-07	-1.6577818	0.06	0.205	0.07834497
chr13-48851292-48852723	3.91E-07	-1.5835026	0.065	0.216	0.09164402
chr6-55256066-55257393	4.13E-07	-1.0084715	0.168	0.385	0.09683537

AT2	p_val	avg_log2FC	pct.1	pct.2	p_val_adj
chr17-39842818-39846951	8.07E-14	0.70923746	0.963	0.922	1.89E-08
chr2-98666906-98667526	1.21E-13	0.48451339	0.969	0.968	2.84E-08
chr17-39847439-39848940	4.26E-13	0.76239297	0.854	0.799	9.98E-08
chr9-35304729-35306073	2.36E-10	0.56723633	0.854	0.753	5.52E-05
chr3-5860222-5861130	4.97E-08	0.84456667	0.53	0.425	0.01164381
chr11-109010949-109012578	5.86E-08	0.75047731	0.611	0.498	0.01374344
chr5-146260501-146261808	4.01E-07	0.87104963	0.479	0.361	0.09397559

Mast Cells	p_val	avg_log2FC	pct.1	pct.2	p_val_adj
chr14-61396648-61397641	1.11E-06	-5.7195666	0.008	0.375	0.25928079

Supplemental Table S4E: Number of Differentially sequenced ATAC chromatin segments with adjusted p values <0.1 or top hit only.

Supplemental Table S4E (cont'd)

AlveolarMacs	p_val	avg_log2FC	pct.1	pct.2	p_val_adj
JH584304.1-58407-60889	3.36E-07	-0.2989984	0.963	0.983	0.07871267
chr7-140873926-140875278	3.49E-07	-2.0317749	0.047	0.213	0.08182869
chr12-104815037-104816057	4.05E-07	-1.4184601	0.111	0.313	0.09502105

Col1a1+ Fibroblasts	p_val	avg_log2FC	pct.1	pct.2	p_val_adj
chr2-98666906-98667526	6.97E-09	0.82419074	0.849	0.653	0.00163362
chr2-98666067-98666807	1.52E-08	0.70022639	0.876	0.774	0.0035694

AT1	p_val	avg_log2FC	pct.1	pct.2	p_val_adj
chr17-39842818-39846951	3.37E-14	0.84839027	0.966	0.929	7.90E-09
chr17-39847439-39848940	3.86E-12	0.87780018	0.82	0.797	9.04E-07
chr17-36230622-36232240	1.49E-10	1.12543015	0.577	0.401	3.49E-05
chr11-109010949-109012578	1.93E-10	1.10506994	0.573	0.44	4.52E-05
chr5-146260501-146261808	2.66E-09	1.13509194	0.532	0.335	0.00062431
chr2-98666906-98667526	1.81E-08	0.54475496	0.91	0.863	0.00423892
chr3-5860222-5861130	5.92E-08	0.99365203	0.524	0.368	0.01387226
chr4-151609967-151611196	1.02E-07	-4.8186827	0.004	0.104	0.02384669
chr10-24835626-24836919	1.99E-07	-3.909238	0.007	0.115	0.04654712

Supplemental Table S4E: Number of Differentially sequenced ATAC chromatin segments with adjusted p values <0.1 or top hit only.

Supplemental Table S4E (cont'd)

Fibroblasts	p_val	avg_log2FC	pct.1	pct.2	p_val_adj
chr2-98666906-98667526	6.46E-15	0.6544296	0.882	0.723	1.51E-09
chr17-39842818-39846951	9.62E-13	0.96049451	0.798	0.652	2.26E-07
chr9-35304729-35306073	2.96E-11	0.72114329	0.685	0.526	6.93E-06
chr16-18339396-18340758	7.18E-10	-1.4383208	0.101	0.285	0.0001682
chr2-166280424-166281354	7.43E-10	-2.5397972	0.025	0.142	0.00017412
chr4-130389017-130390308	1.05E-09	-2.1614836	0.035	0.173	0.00024521
chr15-84697143-84698304	1.72E-09	-1.8820886	0.048	0.2	0.00040384
chr13-60522503-60524050	1.81E-09	-1.9524571	0.043	0.192	0.00042336
chr12-113131725-113134303	8.88E-09	-0.988717	0.204	0.414	0.00208003
chr14-32826747-32829394	9.09E-09	-0.925907	0.217	0.436	0.00213026
chr9-45917025-45918322	9.49E-09	-1.1114441	0.154	0.348	0.00222321
chr17-3463299-3464769	1.38E-08	-2.6217386	0.018	0.123	0.00323765
chr1-36570556-36573310	1.54E-08	-0.9353775	0.209	0.433	0.00360979
chr9-119197140-119198278	1.56E-08	-1.8950709	0.045	0.173	0.00365622
chr4-128733165-128734825	1.56E-08	-0.8805968	0.237	0.46	0.00366232
chr1-92672567-92674446	1.79E-08	-1.1828997	0.131	0.312	0.0042056
chr10-127599857-127601158	2.08E-08	-1.1040331	0.151	0.34	0.00487844
chr16-97751024-97752673	2.19E-08	-1.2722634	0.108	0.288	0.0051389
chr8-68167800-68170293	2.34E-08	-1.8830046	0.045	0.17	0.00548507
chr18-14330710-14332423	2.35E-08	-0.6106242	0.403	0.633	0.00551828
chr13-38878394-38879702	2.68E-08	-2.0670119	0.033	0.153	0.00628408
chr2-27554068-27555069	3.16E-08	-2.90454	0.013	0.104	0.00740638
chr12-16869042-16869871	3.25E-08	-1.9744577	0.035	0.162	0.00762315
chr7-132447956-132449346	3.26E-08	-1.7764666	0.048	0.184	0.00764832
chr12-104406096-104407842	3.46E-08	-1.6508124	0.058	0.2	0.00809702
chr5-52523792-52525289	3.76E-08	-1.6225951	0.058	0.205	0.0088012
chr11-119430796-119432353	4.49E-08	-1.5097686	0.073	0.219	0.01052154
chr4-142756055-142757352	5.71E-08	-1.3241422	0.093	0.26	0.01338909
chr6-140999670-141001649	5.79E-08	-1.5015459	0.076	0.216	0.01356334
chr6-143367185-143368449	6.33E-08	-2.1082531	0.03	0.142	0.01484058
chr16-43945316-43946432	7.18E-08	-1.6994142	0.053	0.184	0.0168255
chr2-167843829-167845408	8.70E-08	-1.1064008	0.136	0.315	0.02038655
chr16-4652399-4653728	1.31E-07	-1.6486865	0.055	0.186	0.03071163
chr18-75502291-75504436	1.34E-07	-0.7477088	0.29	0.501	0.03142737
chr1-93209735-93211429	1.35E-07	-1.2014265	0.108	0.288	0.03170083
chr12-113141948-113143681	1.39E-07	-1.0014795	0.171	0.353	0.03246702
chr11-47705004-47706220	1.54E-07	-1.7350843	0.048	0.173	0.03605931
chr6-136906020-136907874	1.63E-07	-1.137594	0.128	0.29	0.03813241
chr19-24307293-24308784	1.85E-07	-1.945982	0.035	0.151	0.0433634
chr5-65456482-65459036	2.07E-07	-1.144343	0.121	0.288	0.04841435
chr6-49095822-49097613	2.19E-07	-0.6784696	0.312	0.542	0.05133466
chr17-45583460-45586366	2.30E-07	-1.0635095	0.149	0.321	0.05378701
chr4-117846805-117847842	2.52E-07	-1.7491943	0.043	0.167	0.05909187
chr6-111584373-111585533	2.52E-07	-0.8674691	0.212	0.389	0.05916818
chr17-39847439-39848940	2.61E-07	0.89125903	0.494	0.389	0.06124624
chr11-11856425-11857560	2.63E-07	-1.2572616	0.098	0.249	0.0615866
chr4-111624212-111625660	2.76E-07	-1.5419556	0.06	0.192	0.06463287
chr1-177014257-177015266	2.89E-07	-1.7883126	0.043	0.159	0.06775598
chr7-117493916-117495761	3.13E-07	-0.9985542	0.159	0.334	0.07345963
chr14-79034135-79035904	3.39E-07	-1.9332915	0.035	0.142	0.0793698
chr14-24747413-24748971	3.70E-07	-1.1649663	0.116	0.271	0.08664045
chr8-12788226-12789435	3.72E-07	-1.4125725	0.073	0.211	0.08728403
chr12-38136093-38137358	3.93E-07	-1.0957756	0.134	0.285	0.09216294

Supplemental Table S4E: Number of Differentially sequenced ATAC chromatin segments with adjusted p values <0.1 or top hit only.

Supplemental Table S4F

	p_val	avg_log2FC	pct.1	pct.2	p_val_adj
Il1b	0.21248498	0.38126016	0.102	0.095	1
Il18	0.19556494	0.67364294	0.057	0.05	1

	p_val	avg_log2FC	pct.1	pct.2	p_val_adj
**il1b chr2-129,206,490–129,213,059**					
chr2-129206405-129207502	0.00010375	-0.5754807	0.028	0.047	1
chr2-129209899-129210819	0.0100254	-0.4468081	0.023	0.034	1
chr2-129207932-129208895	0.07132926	-0.3216594	0.018	0.025	1
chr2-129212226-129213333	0.01171048	-0.3707286	0.024	0.035	1
**il18 chr9-50,466,127 and 50,493,140**					
chr9-50467079-50468445	0.03935128	-0.3111103	0.029	0.039	1
chr9-50470068-50471506	0.01072068	-0.2808366	0.043	0.058	1
chr9-50478300-50480249	1.99E-08	-0.59697	0.069	0.109	0.00466045
chr9-50482277-50483455	0.00016391	-0.536613	0.032	0.051	1
chr9-50490313-50492497	3.35E-05	-0.4877965	0.041	0.065	1

Supplemental Table S4F: IL-1b and IL-18 ATAC seq differences between FR and FF lungs (all cell types combined, pseudobulk analysis)
